# Νovel Polylactic Acid/Tetraethyl Citrate Self-Healable Active Packaging Films Applied to Pork Fillets’ Shelf-Life Extension

**DOI:** 10.3390/polym16081130

**Published:** 2024-04-17

**Authors:** Vassilios K. Karabagias, Aris E. Giannakas, Nikolaos D. Andritsos, Dimitrios Moschovas, Andreas Karydis-Messinis, Areti Leontiou, Apostolos Avgeropoulos, Nikolaos E. Zafeiropoulos, Charalampos Proestos, Constantinos E. Salmas

**Affiliations:** 1 Department of Food Science and Technology, University of Patras, 30100 Agrinio, Greece; vkarampagias@upatras.gr (V.K.K.); nandritsos@upatras.gr (N.D.A.); aleontiu@upatras.gr (A.L.); 2 Department of Material Science and Engineering, University of Ioannina, 45110 Ioannina, Greece; dmoschov@uoi.gr (D.M.); karydis.and@gmail.com (A.K.-M.); aavger@uoi.gr (A.A.); nzafirop@uoi.gr (N.E.Z.); 3 Laboratory of Food Chemistry, Department of Chemistry, National and Kapodistrian University of Athens Zografou, 15771 Athens, Greece; harpro@chem.uoa.gr

**Keywords:** polylactide acid, tetraethyl citrate, self-healing, active packaging, mechanical properties, water/oxygen barrier properties, antioxidant activity, antibacterial activity, pork fillets shelf life

## Abstract

Nowadays, increased food safety and decreased food waste are two of the major global interests. Self-healable active packaging materials are an attractive option to achieve such targets. This property is critical for the hygiene and the consumption appropriateness of the food. Polylactic acid is a very promising polymeric matrix that potentially could replace the widely used low-density polyethylene due to its biobased origin and its easy biodegradable nature. The main drawback of this polymeric matrix is its brittle, fragile nature. On the other hand, tetraethyl citrate is a biobased approved food additive which became an attractive option as a plasticizer for industries seeking alternative materials to replace the traditional petrochemically derived compounds. A novel biobased film exhibiting self-healing behavior suitable for food-active packaging was developed during this study. Polylactic acid’s brittleness was reduced drastically by incorporating tetraethyl citrate, and a random cut on the original self-repairing film was fully healed after 120 s. The optimum concentration of tetraethyl citrate in the polylactic acid was around 15% *v*/*w* with a water/oxygen barrier close to the relevant of polylactic acid and low migration. According to the EC_50_ parameter, the antioxidant activity was 300% higher than the relevant of pure polylactic acid, while according to the thiobarbituric acid and heme iron parameters, the film resisted lipid oxidation and deterioration. Finally, the total viable count parameter indicates the strong antimicrobial activity of this sample.

## 1. Introduction

Today, the global trends of sustainability and the circular economy require the use of new biobased materials or materials derived from food by-products and/or the processing of agricultural waste [1,2,3,4]. In the field of Food Science and Technology, this trend has pushed researchers to the need to use new biodegradable and environmentally friendly food packaging films [5,6,7]. The most widely used plastic products are packaging films, the waste of which has caused almost irreversible environmental damage and is a very serious issue that demands solutions worldwide [8,9,10]. As reported by European Bioplastic, the total bioplastic production capacity is expected to increase from about 2.11 million tons in 2019 to about 2.43 million tons in 2024 [11,12]. Among the biobased and biodegradable polymers, polylactic acid (PLA) has the greatest potential to be used as a raw material for food packaging films and, thus, to replace conventional plastics derived from fossil fuels [12,13,14,15,16]. PLA comes from renewable sources such as corn starch, tapioca roots, starch, or sugar cane, which are common agricultural wastes [12,17,18,19]. Compared to petroleum-based polymers, PLA requires 25–55% less energy to be produced and releases 15–60% less carbon dioxide [12,17,18,19], while its barrier properties are similar to those of polyethylene-terephthalate (PET). However, PLA’s poor viscoelastic behavior and extremely low melting strength are inhibiting factors for film packaging production due to the need for low-cost packaging and the widely used hot blow molding process.

To increase flexibility, PLA is mixed with plasticizers, but these must maintain PLA’s environmentally friendly profile [20]. Recent studies have shown that the most promising plasticizers that could be combined with PLA in food packaging applications are epoxidized soybean oil, cardanol, and citrate esters such as triethyl citrate (TEC) and acetyl tributyl citrate (ATBC) [21,22]. Epoxidized soybean oil is derived from soybeans and has recently been shown to increase the elongation at break, lower the glass transition temperature, and increase the mobility of PLA chains [21,23]. Cardanol is derived from cashew shells and has recently shown good miscibility at low contents with PLA and significantly reduced the glass transition temperature [24]. It also enhanced the crystallization ability of PLA, exhibiting good plasticization performance [25,26,27]. Citrate esters such as triethyl citrate (TEC) and acetyl tributyl citrate (ATBC) are biobased compounds that have recently been shown to have great potential to be used as plasticizers of PLA to improve its thermomechanical properties and greatly enhance its flexibility [25,28,29]. TEC is a citric acid ester that is used as a food additive (E number E1505) to stabilize foams (especially as a whipping aid for egg whites), as a flavoring agent in foods, and as a solvent and surface-active agent [30]. It is a colorless, odorless liquid that is also used in pharmaceutical coatings and plastics [30].

Maiza et al. [26] used TEC and ATBC as plasticizers for PLA-based films. Both TEC and ATBC were blended with PLA with the melt extrusion process at 5%, 10%, 15%, 20%, and 30% by weight. The plasticization effect of both TEC and ATBC with PLA was analyzed using differential scanning calorimetry (DSC), dynamic mechanical analysis (DMA), X-ray diffraction (XRD), and Fourier transform infrared (FTIR) spectroscopy. It was found that the glass transition temperature (T_g_) and melting temperature (T_m_) of the obtained composites decreased as the amount of both plasticizers increased [26]. Additionally, the presence of TEC or ATBC tended to increase the crystallinity of PLA [26]. In a similar recent report [28], the plasticization effect of TEC and ATBC with PLA was studied with DSC, DMA, XRD, and FTIR measurements, and the authors concluded that the higher the molecular weight of citrate used, the greater the plasticizing effect on PLA [28]. In this study, the transparency of the obtained PLA/TEC and PLA/ATBC composite was also studied with UV-vis measurements, and no effect on the color change of the obtained films was observed [28]. Finally, the migration of both TEC and ATBC as a function of the used heating temperature was studied, which showed that the loss of both plasticizers resulted in cracks and color changes in the obtained composite films [28].

Herein, PLA and PLA/TECx composite films were obtained by applying the melt extrusion method, varying the % *v*/*w* TEC content (5, 10, 15, 20, 25, and 30 mL in 100 g of PLA), and characterizing the films physiochemically with XRD, FTIR, and scanning electron microscopy (SEM) measurements. The plasticization effect of TEC in the obtained PLA/TECx composite films was evaluated with tensile and dynamic thermomechanical properties measurements. The dynamic thermomechanical analysis was carried out using a dynamic mechanical analyzer (DMA). Packaging properties such as the TEC migration test, self-healing properties, water/oxygen barrier properties, antioxidant activity with 2,2-diphenyl-1-picrylhydrazyl (DPPH) assay, and antibacterial activity against *Salmonella Typhimurium* and *Staphylococcus aureus* were investigated. After evaluating all the packaging properties studied, the PLA/TECx composite film with the greatest potential for use as a packaging film was applied to pork fillets as an active wrapping film, and their shelf life was evaluated with lipid oxidation determination using the thiobarbituric acid reactive substances (TBARS) method, the determination of heme iron contents, total viable count values (TVC), and sensory analysis.

The specific innovative points of the current study are summarized as follows: (i) PLA/TEC composite films have been developed and studied in the past, but the self-healing properties, water/vapor barrier properties, antioxidant activity properties, and antibacterial properties for such PLA/TEC composite films are studied and reported for the first time; and (ii) the obtained antioxidant properties and antibacterial properties of such PLA/TEC composite films led us to apply for the first time such PLA/TEC films to fresh pork fillet active packaging films to extend the fresh pork fillets’ shelf life.

## 2. Materials and Methods

### 2.1. Chemicals and Reagents

Polylactic acid (PLA), Ingeo™ Biopolymer 3052D, was purchased from Nature Works LLC (Minnetonka, MN, USA). Triethyl citrate (TEC) was purchased from Alfa Aesar GmbH and Co KG, (Karlsruhe, Germany). Methanol absolute for analysis, acetate buffer (CH_3_COONa·3H_2_O), trichloroacetic acid, and 2-thiobarbituric acid were purchased from Merck. 2,2-diphenyl-1-picrylhydrazyl (DPPH) was purchased from Sigma-Aldrich (Darmstadt, Germany). Acetone of analytical grade was purchased from Fisher Scientific (Bishop Meadow Road, Loughborough, UK).

### 2.2. Experimental Design—Preparation of Extruded PLA/TEC Composite Pellets

All PLA/TEC composite pellets were developed using a Mini Lab twin-screw extruder (Haake Mini Lab II, Thermo Scientific, ANTISEL, S.A., Athens, Greece) where the operating speed and temperature were 120 rpm and 180 °C, respectively.

PLA/TEC composite blends were developed to investigate the role of % *v*/*w* TEC content in the obtained PLA/TEC composites. For each composite of this first group, 4 g of PLA were extruded with 0.2, 0.4, 0.6, 0.8, 1.0, and 1.2 mL of TEC, respectively, and the obtained blends were labeled as PLA and PLA/TECx, where x = 0.2, 0.4, 0.6, 0.8, 1.0, and 1.2 mL of additional TEC in 4 g of PLA. The sample names, PLA and TEC contents, operating temperature, and operating speed of the twin extruder used for the development of all PLA/TECx composite blends are listed in Table 1 for comparison.

### 2.3. Films Formation

All PLA/TEC_x_ composite pellets obtained after extrusion and the pure PLA pellets were thermomechanically converted into films using a hydraulic press with heated plates and thermal pressure. Approximately 1.0 g of pellets and a constant pressure of 0.5 MPa, at 170 °C, were used to obtain films with an average diameter of 11 cm and a thickness of 0.05–0.11 mm.

### 2.4. Physicochemical Characterization of PLA/TECx Composite Films

All the obtained PLA/TECx composite films and the PLA films were physiochemically characterized using X-ray powder diffraction analysis (XRD), Fourier transform infrared spectroscopy (FTIR), scanning electron microscopy (SEM), tensile properties measurements, and dynamic mechanical analysis (DMA). All XRD measurements were conducted using a Brüker XRD D8 Advance diffractometer (Brüker, Analytical Instruments, S.A., Athens, Greece). FTIR analysis measurements were carried out with an FT/IR-6000 JASCO Fourier transform spectrometer (JASCO, Interlab, S.A., Athens, Greece).

Scanning electron microscopy (SEM) images (cross-section) were obtained using a JEOL JSM-6510 LV SEM Microscope (JEOL Ltd., Tokyo, Japan) equipped with an X–Act EDS-detector by Oxford Instruments, Abingdon, Oxfordshire, UK (an acceleration voltage of 20 kV was applied). Prior to the SEM analysis, the samples of PLA and plasticized PLA formulations were coated under vacuum conditions with a gold/palladium (Au/Pd) thin layer (4–8 nm) in a sputtering machine (SC7620, Quorum Technologies, Lewes, UK) to enhance their electrical conductivity.

Measurements of tensile properties were performed according to the ASTM D638 method reported elsewhere [30,31,32] using a Simantzü AX-G 5kNt instrument (Simantzü. Asteriadis, SA, Athens, Greece). A dynamic mechanical analyzer (DMA Q800, TA Instruments, New Castle, DE, USA) in membrane voltage mode was used for DMA. The methodology used for all physicochemical characterization measures was similar to that used in recent reports [30,31]. Measurements of tensile properties were performed according to the ASTM D638 method using a Simantzü AX-G 5kNt instrument (Simantzü. Asteriadis, S.A., Athens, Greece). A dynamic mechanical analyzer (DMA Q800, TA Instruments) in film tension mode was employed for DMA. The methodology used for all physicochemical characterization measurements was similar to that used in recent reports [31,32].

### 2.5. TEC Migration Test

TEC migration from all obtained PLA/TECx composite films was performed according to the gravimetrical method described by Maiza et al. [28] with some modifications. Briefly, the films (three samples from each film) were placed in a ventilated oven at an isothermal temperature of 72 °C for 1 h. The temperature of 72 °C and the residence time of 1 h were chosen to simulate the most common pasteurization process conditions commonly used in the food industry [33]. All films were weighed before (*m*_0_) and after (*m_t_*) 1 h at 72 °C. Before the final weighing, the surface of the migrated plasticizer was gently cleaned. The weight loss of the plasticizer was calculated as the total weight loss of the samples during the heating time (*t*) and was calculated according to Equation (1):

(1) $$\%\;weight\;loss=\frac{m_{0}-m_{t\;}}{m_{0}}\times 100$$

### 2.6. Characterization of Active Packaging Properties of Obtained PLA/TEC Composite Films

#### 2.6.1. Water/Oxygen Barrier Properties

The water vapor transmission rate (WVTR) values of all obtained PLA/TECx composite films, as well as the PLA films (three samples each), were determined with a handmade apparatus according to the ASTM E96/E 96M-05 method reported elsewhere [34]. From the obtained WVTR values, the water vapor diffusion coefficient values (D_wv_) were calculated according to the methodology described in detail recently [34]. For PLA and each PLA/TECx composite film, four to seven different samples were measured, and the mean values were calculated. The oxygen transmission rate (OTR) values of all obtained PLA/TEC composite films and the pure PLA film were measured using an oxygen permeation analyzer (O.P.A., 8001, Systech Illinois Instruments Co., Johnsburg, IL, USA) according to the ASTM D 3985 method (23 °C and 0% RH) reported elsewhere [34]. The obtained OTR values were transformed into oxygen diffusion coefficient (P_O2_) values using the methodology described in detail in a recent report [35]. For PLA and each PLA/TECx composite film, at least three different samples were measured, and the mean values were calculated.

#### 2.6.2. In Vitro Antioxidant Activity Determination of the Obtained PLA/TECx Composite Films

##### Preparation of [DPPH^•^] Free Radical Standard Solutions

A methanolic [DPPH^•^] free radical standard solution was prepared according to the methodology described by Mishra et al. [36,37]. Briefly, 0.0212 g of [DPPH^•^] free radical was dissolved in 250 mL of methanol to obtain a solution with a molarity of 2.16 mM (mmol/L). Next, the flask was subjected to vortex mixing under dark conditions. The resultant [DPPH^•^] free radical methanolic solution exhibited a deep purple color and had a neutral pH of 7.02 ± 0.01. To ensure stability, the solution was refrigerated under dark conditions for 2 h before use. Once the free radical was stable, appropriate dilutions were carried out to establish the calibration curve.

##### Preparation of [DPPH^•^] Free Radical Calibration Curve

A calibration curve of concentration versus absorbance of [DPPH^•^] was prepared as follows: the 2.16 mM (mmol/L) methanolic solution of [DPPH^•^] was diluted by adding appropriate volumes of methanol to obtain a solution with a concentration range from 0 to 50 mg/L. The resulting solutions were vortexed and left in the dark, and the absorbance was measured with a SHIMADZU UV-1280 UV/VIS Spectrometer (Simantzü. Asteriadis, S.A., Athens, Greece) at 517 nm. The calibration curve of absorbance (y) versus concentration (x) of [DPPH^•^] free radical was expressed by the following equation:

y = 0.0388x + 0.015; R^2^ = 0.9994 (2)

A standard solution of low concentration [DPPH^•^] (0–50 mg/L) was used, basically, for two reasons: (a) for the obtained values of absorbance (0.0001–1.942 absorbance units) to obey the law of Beer–Lambert and (b) for the lesser stabilization time and complete dilution of the free radical in the methanolic matrix.

#### 2.6.3. Determination of EC_50_ (Concentration Required to Obtain 50% Antioxidant Effect) Antioxidant Activity of PLA/TECx Films

To determine the effective concentration (EC_50_) of the obtained PLA/TECx films and the PLA film, 10, 20, 30, 40, and 50 mg of each film were placed, in triplicates (*n* = 3), in dark vials. After that, volumes of 3 mL of methanol solution of [DPPH^•^] free radical (0.024 mg/mL, 6.11 × 10^−5^ mol/L) and 2 mL of acetate buffer 100 mM (pH = 7.10) were added in a vial, and the absorbance of the [DPPH^•^] free radical was measured at t = 0 (A_0_ = 0.737). Subsequently, to each sample, 3 mL of [DPPH^•^] free radical methanolic solution and 2 mL of acetate buffer were added. The absorbance of the reaction mixture was measured at 517 nm after 24 h. The % inhibition of [DPPH^•^] was calculated using the following equation:

(3) $${\%\;\mathrm{scavenged}\;\mathrm{DPPH}}^{•}\;\mathrm{at}\;\mathrm{steady}\;\mathrm{state}\;=\frac{A_{0}^{517}-A_{\mathrm{sample}}^{517}}{A_{0}^{517}}\times 100$$

Next, the calculated values of % antioxidant (% inhibition of [DPPH^•^]) activity of films were plotted as a function of film quantity used, and the linear equation from the obtained plot was calculated. From the calculated linear equations (see Figures S1–S7) for each sample, the EC_50_ value was calculated.

#### 2.6.4. Antibacterial Activity of Obtained PLA/TEC Composite Films

The antimicrobial activity of the obtained PLA and PLA/TECx composite films was tested against two major foodborne pathogenic bacteria of different Gram strains, namely the Gram-positive *Staphylococcus aureus* (NCTC 6571) and the Gram-negative *Salmonella enterica* subspecies *enterica* serovar *Typhimurium* (NCTC 12023). The bacteria were supplied as microbiological certified reference materials in the form of *easi*-tab™ pellets (*S. aureus*) by LGC Standards Proficiency Testing (Chamberhall Green Bury, Lancashire, UK) and in the form of disc-shaped Vitroids™ (*S. Typhimurium*) by Supelco^®^ Analytical Products, a subsidiary of Merck (Darmstadt, Germany).

The experimental procedure that followed was based on the agar diffusion method described by Giannakas et al. [35], with some slight modifications of the original protocol given by Barmpaki et al. [38]. Briefly, a final concentration of approximately 1.5 × 10^8^ CFU/mL (i.e., 0.5 McFarland standard) per bacterial strain was achieved in 3 mL of 0.85% peptone salt solution or maximum recovery diluent (MRD; Merck). The suspended bacteria in MRD were inoculated on Mueller–Hinton (MH) agar (Oxoid, Basingstoke, UK) plates with the help of sterile cotton applicators (Jiangsu Kangjin Medical Instrument Co., Taizhou, China) and samples of the PLA/TEC composite films with a diameter of 6 mm were placed onto the surface of MH agar. Then, incubation of the inoculated MH agar plates seeded with the sample composite films took place at 37 °C for 18–24 h. The diameters of inhibition zones in the contact area of the films and around them, if applicable, were measured using a Vernier caliper with 0.1 mm accuracy. The experimental procedure was repeated twice, and films were measured in triplicate in each repetition.

### 2.7. Packaging Test of Fresh Pork Fillets Wrapped with PLA and PLA/TEC0.6 Films

#### 2.7.1. Packaging Preservation Test of Pork Meat Fillets

Pork meat fillets were supplied by a local meat processing factory, Aifantis, and transferred immediately to the laboratory. Pork fillets, portions of 80–90 g each, were packaged aseptically on the paper that Aifantis Company uses for commercial packaging (control sample) and between two films 11 cm in diameter, of PLA and PLA/TEC0.6. Afterwards, the PLA and PLA/TEC0.6 treatments were folded inside the paper, without the inner membrane, that Aifantis Company uses for packaging. Samples for 2, 4, and 6 days of preservation were prepared for all the tested packaging systems. After packaging, the fillets were placed in a preservation chamber at 4 ± 1 °C and measured for lipid oxidation, heme iron content, total viable count, and the sensory analysis test.

#### 2.7.2. Lipid Oxidation of Pork Fillets with Thiobarbituric Acid Reactive Substances

The thiobarbituric acid reactive substances (TBARS) values of the packaged pork fillets were determined using the method of Tarladgis et al. [39] with some modifications. In brief, 2 g of meat sample was placed in a vial along with 5 mL of 10% (*w*/*v*) trichloroacetic acid (TCA) solution. The mixture was then vortexed for 5 min, and then 5 mL 0.02 M aqueous solution of 2-thiobarbituric acid was added to it and vortexed again for 5 min. The mixture was left in the dark for 18–24 h for color development. The resultant supernatant was pipetted off and centrifuged, and the absorbance (D) was measured against the blank sample, 5 mL of 10% (*w*/*v*) TCA solution, and 5 mL of 0.02 M aqueous solution, at λ = 538 nm using glass cuvettes of 1 cm.

TBARS was expressed as mg of malondialdehyde (MDA)/kg of the sample according to the following equation:

TBARS (mg/kg) = 3.6 × D(4)

#### 2.7.3. Heme Iron Content

The heme iron content of the packaged fresh pork fillets was determined according to the method reported by Clark et al. [40], with some modifications. In particular, 4 g of pork fillets were homogenized with a mixer (Vicko S.A, Athens, Greece) in 18 mL of acidified acetone. Then, the solution was left to stand at 25 °C, protected from light, for 1 h. Afterward, the solution was filtered, and the absorbance was measured at 640 nm using a UV-vis spectrophotometer (SHIMADZU UV-1280). The amount of heme iron in the pork fillets was calculated according to the following equation:

Heme iron (μg/g) = A_640_ × 680 × 0.0882 (5)

where A_640_ is the absorbance measured at 640 nm, and 680 and 0.0882 are constant values in the equation. Heme iron content analyses were conducted every 2 days up to 6 days of storage at 4 ± 1 °C.

#### 2.7.4. Total Viable Count (TVC) of Pork Fillets

TVC was monitored with respect to storage time at 4 ± 1 °C (0, 2, 4, and 6 days). Ten grams of pork fillets were removed aseptically from each packaging system and transferred to a stomacher bag (Seward Medical, Worthing, West Sussex, UK), containing 90 mL of sterile buffered peptone water (ΒPW, NCM0015A, Heywood, UK; 0.1 g/100 mL of distilled water) and homogenized using a stomacher (LAB Blender 400, Seward Medical, UK) for 90 s at room temperature. For the microbial enumeration, 0.1 mL of serial dilutions (1:10 diluents, buffered peptone water) of pork meat homogenates was spread on the surface of plate count agar (PCA, NCM0010A, Heywood, UK). TVC was determined after incubation for 2 days at 30 °C [41].

#### 2.7.5. Sensory Analysis of Pork Fillets

The sensory properties of pork meat were scaled from 0 (for the least liked sample) to 5 (most liked sample) points by seven experienced members of the Food Science and Technology Department. On each sampling day, color, odor, and cohesion were evaluated [42].

### 2.8. Statistical Analysis

All data acquired from the water vapor/oxygen barrier and mechanical properties measurements, along with antioxidant activity, thiobarbituric acid reactive substances, heme iron content, total viable count, and sensory analysis scores, were subjected to statistical analysis to identify any statistical differences. Three different species of each kind of film were measured. The commonly used ANOVA method could not be employed in this case because such a low number of measurement repetitions (i.e., three) could not provide accurate normality and homogeneity test results. This could impose uncertainty on the results of the investigation of mean values of statistical difference. Thus, a non-parametric statistical procedure should be used for such analysis. Among the famous Kruskal–Wallis and Mood’s median test methods, the second was chosen because some extreme outliers existed between the experimental points, and this method is more robust to the outliers’ treatment than the first one. Overall runs as well as multiple comparison tests between all combinations of sample couples were carried out. For all groups of experimental measurements, the statistical analysis interpretation was carried out assuming a significance level of *p* < 0.05, and using the SPSS software (v. 28.0, IBM, Armonk, NY, USA).

## 3. Results

### 3.1. Self-Healing Properties

**Figure 1:**
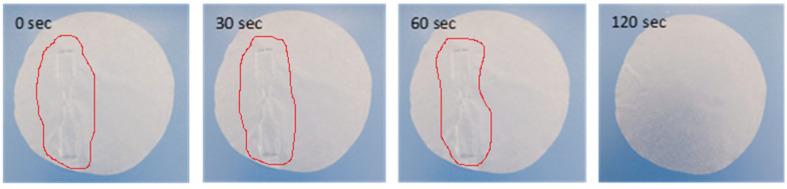
Images of PLA/TEC_0.6_ film during the self-healing process.

Starting with the well-known Greek proverb that an image is worth a thousand words, we present the self-healing process experiment of PLA/TEC_0.6_ composite film in Figure 1 and in Videos S1 and S2 of the Supplementary Materials. Self-healing properties are very important for a polymer or biopolymer matrix and are defined as the ability of polymers/biopolymers to convert physical energy into a chemical and/or physical response to heal the damage [43]. Self-healing polymers/biopolymers respond to external stimuli to recover the initial properties of the material. So, self-healing properties could give new application prospects to such active packaging films. To demonstrate the self-healing properties of PLA/TEC_x_ composite films, a dog bone shape was cut in a PLA/TEC_0.6_ film with a diameter of 10 cm and allowed to stabilize in open air to observe possible self-healing properties (see Figure 1 and Videos S1 and S2).

As observed in Figure 1 and Video S1 of the Supplementary Materials, the cut region of the dog bone-shaped film starts to reattach with the rest of the film from the first seconds. Resealing is evident after 60 s, while after 120 s, the film has fully resealed and returned to its original state. It should be stated that such self-healing properties are reported for the first time for such PLA/TEC_x_ composite materials. In advance, as observed in Video S2, the obtained self-healing mechanism in such PLA/TEC_x_ composite films is thermos-responsive [43].

### 3.2. Physicochemical Characterization of PLA/TEC Composite Films with XRD Analysis, FTIR Spectroscopy and SEM Images

**Figure 2:**
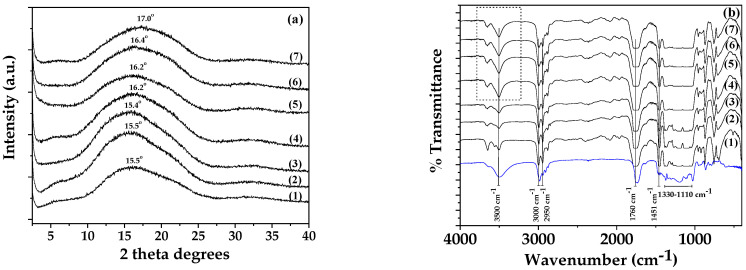
(**a**) XRD plots of (1) pure PLA, (2) PLA/TEC_0.2_, (3) PLA/TEC_0.4_, (4) PLA/TEC_0.6_, (5) PLA/TEC_0.8_, (6) PLA/TEC_1.0_, and (7) PLA/TEC_1.2_ obtained films, (**b**) FTIR plots of pure TEC (blue line plot), (1) pure PLA, (2) PLA/TEC_0.2_, (3) PLA/TEC_0.4_, (4) PLA/TEC_0.6_, (5) PLA/TEC_0.8_, (6) PLA/TEC_1.0_, and (7) PLA/TEC_1.2_ obtained films.

In Figure 2, the XRD (Figure 2a) and FTIR (Figure 2b) plots of all obtained PLA/TEC_x_ films as well as pure PLA films are shown for comparison.

As shown in Figure 2a, the XRD plot of pure PLA film has a broad peak at around 15.5° 2-theta, which corresponds to its amorphous crystal phase. As shown in the XRD plots of PLA/TEC_x_ composite films, this broad peak remains the same in the case of PLA/TEC_0.2_ and PLA/TEC_0.4_ films while it is shifted in higher 2-theta values for PLA/TEC_0.6_, PLA/TEC_0.8_, PLA/TEC_1.0_, and PLA/TEC_1.2_ composite films. This shift of the PLA peak is attributed to the relaxation caused by the incorporation of TEC molecules inside PLA chains, which increased the % *v*/*w* TEC content. This result is in accordance with a previous report that showed that the concentration of TEC added affected PLA’s obtained crystallinity [26].

In Figure 2b, the FTIR plots of pure TEC and all obtained PLA/TEC_x_ films as well as pure PLA films are shown for comparison.

In the FTIR plot of pure PLA (see line (1) in Figure 2b), the following characteristic absorption bands are observed: the stretching vibration at 3500 cm^−1^ of the hydroxyl (O–H) group of PLA, the asymmetrical and symmetrical stretching bands at 2950, 3000, and 3050 cm^−1^ of the methyl group (–C–H) in the side chains of PLA, the stretching vibration at 1760 cm^−1^ of the carbonyl group (C=O) from the repeated ester unit of PLA, the stretching vibration at 1110–1330 cm^−1^ of the ether (–C–O–) group of PLA, and the bending vibrations at 1451 cm^−1^ of methyl (–CH_3_) group.

In the FTIR plot of pure TEC (see the blue line plot in Figure 2b), the following characteristic absorption bands were observed: the stretching vibration at 3496 cm^−1^ of the hydroxyl (O–H) group of TEC, the asymmetrical and symmetrical stretching bands at 2985, 2941, and 2909 cm^−1^ of the methyl group (–C–H) of TEC, the stretching vibration at 1741 cm^−1^ of the carbonyl group (C=O) of TEC, and bands between 1100 and 1330 cm^−1^ that corresponded to the ether (–C–O–) and –CH_2_– groups of the TEC chain [44]. Thus, at first glance, the FTIR plot of TEC looks very similar to the FTIR plot of PLA, with the same bands moving slightly on lower wavelengths.

A careful glance at the FTIR plots of the PLA/TECx composites indicated that there is some molecular interaction between PLA and TEC. The plots of the PLA/TEC_0.2_ and PLA/TEC_0.4_ composites are almost the same as the plot of pure PLA. In contrast, in the plots of PLA/TEC_0.6_, PLA/TEC_0.8_, PLA/TEC_1.0_, and PLA/TEC_1.2_, an increment of bands at 3500 is observed (see the denoted with dot line triangular region). This increment of the hydroxyl group of PLA is in accordance with the shift of the PLA amorphous peak observed in the XRD plots. Both observations suggest the interaction of PLA chains with TEC molecules when the TEC concentration increased up to 15% *v*/*w*. According to the literature, this interaction between PLA and TEC may be attributed to the possible hydrogen bonding that occurs between the C=O group in TEC and the small number of terminal hydroxyl groups in the PLA main chain [26,29].

**Figure 3:**
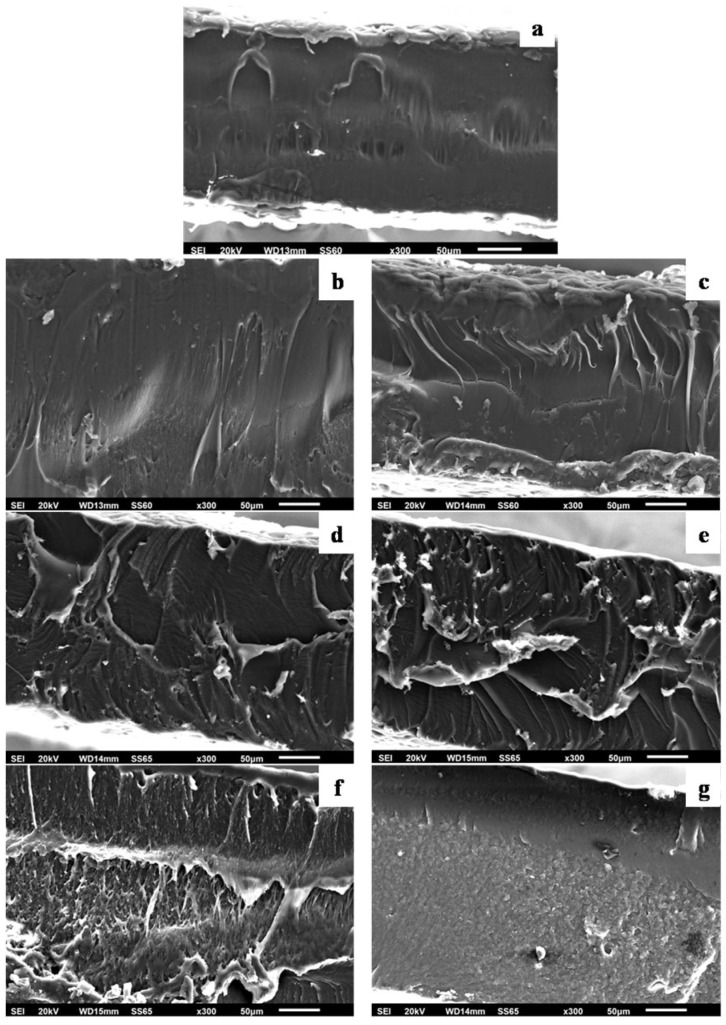
SEM micrographs of the cross-section morphology of PLA/TEC films. (**a**) 0% *v*/*w*, (**b**) 5% *v*/*w*, (**c**) 10% *v*/*w*, (**d**) 15% *v*/*w*, (**e**) 20% *v*/*w*, (**f**) 25% *v*/*w*, and (**g**) 30% *v*/*w* TEC (magnification ×300, scale bar 50 μm).

Scanning electron microscopy (SEM) can be used to investigate the material’s behavior based on the effect of plasticizer level on films and the surface features. Thus, morphological characteristics of the cross-sections of film samples were evaluated with SEM. The surface images of the prepared films are presented in Supplementary Figure S8. It is obvious from such pictures that films shown in Figure S8d,e exhibited low surface roughness and homogenous surface dispersion. For a more informative view, cross-section images were captured. The cross-section images of neat PLA and PLA via plasticizer TEC with different concentrations (5, 10, 15, 20, 25, and 30% *v*/*w* TEC) are shown in Figure 3.

All films obtained were clear and uniform in thickness. The surface of the neat PLA film (Figure 3a) revealed smooth surface morphology and a continuous phase without any evidence of heterogeneities. The SEM micrographs (Figure 3b,c) plasticized with lower amounts of TEC (5 and 10% *v*/*w*) showed surface morphology similar to pure PLA with low dispersion and non-embedded plasticizer in the PLA matrix. When further increasing the TEC content to 15 and 20% (*v*/*w*; Figure 3d,e), rougher surfaces and a few long threads of the composite were occasionally discernible. Small white spots on the cross-section of the PLA/TEC films were observed, and they became denser with increasing TEC content. No inhomogeneity was detected on the composite surface, which suggests excellent compatibility with good dispersion between the plasticizer TEC and polymer matrix PLA,t which was the major parameter hat improved the behavior of the films. In the cases of the modified PLAs with the addition of TEC contents (25 and 30% *v*/*w*; Figure 3f,g), the surface showed greater roughness as the TEC concentration increased in the matrix. Some aggregates and voids were evidenced on the film surface where TEC had accumulated. Since we were able to deduce changes in the structures of the PLA composites with different contents of TEC, we can conclude that the optimal TEC concentrations are evident between 15 and 20% (*v*/*w*), as the plasticizer can be better incorporated into the amorphous parts of the PLA polymer matrix with complete mixing and homogeneous morphology.

### 3.3. Mechanical and Thermomechanical Properties of PLA/TECx Composite Films

In Table 2, the calculated values obtained from stress–strain curves, including mean values of modulus of elasticity (E), tensile strength (σ_uts_), and % elongation at break (ε_b_) of all tested PLA/TEC_x_ films as well as PLA films, are listed for comparison. Descriptive statistics from the statistical analysis are presented in Supplementary Table S1, while the significance of the difference of the mean values of E, σ_uts_, and ε% among the tested samples are listed in Supplementary Tables S2–S5. A general result from Table S2 is that, considering each tensile property separately, there are statistically significant differences between the mean values of all tested samples. A more detailed pairwise comparison for each tensile parameter is presented in Tables S3–S5. Table 2 also shows the values of glass transition temperature (T_g_) calculated from storage modulus and tan delta plots.

The statistical results of Table 2 are also presented graphically in Figure S9 via box-plots graphs.

**Figure 4:**
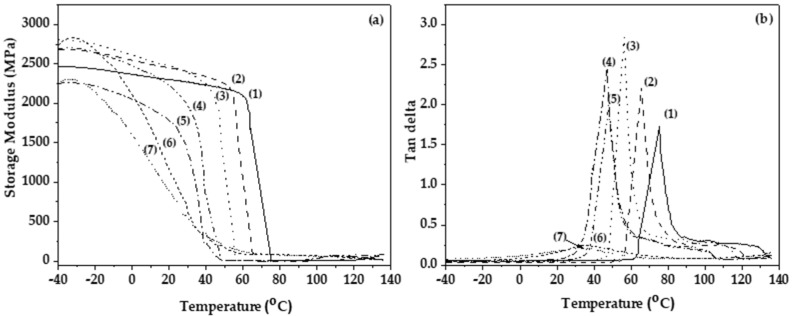
(**a**) Storage modulus and (**b**) tan delta plots as a function of the temperature of (1) PLA, (2) PLA/TEC_0.2_, (3) PLA/TEC_0.4_, (4) PLA/TEC_0.6_, (5) PLA/TEC_0.8_, (6) PLA/TEC_1.0_, and (7) PLA/TEC_1.2_ obtained films.

As shown in Table 2, the addition of the TEC content in PLA causes a decrease in both Young’s Modulus (E) and ultimate strength values (σ_uts_) and an increase in % elongation at break values (ε%), compared to the initial pure PLA films. This result is also confirmed the Figure 4. This behavior is expected when the plasticization process takes place and is in accordance with conclusions reported in the literature [26,29]. Statistical results derived from the median test method showed statistically significant differences for all tensile properties between samples PLA/TEC_0.6_ and PLA/TEC_0.8_, while samples before or after these two materials did not exhibit significantly different tensile values.

Figure 4a,b shows the measurements of the dynamic mechanical analysis (DMA), storage modulus, and tan delta for all tested PLA/TEC_x_ films as well as pure PLA film.

As shown in Figure 4a, the incorporation of TEC in low concentrations does not significantly affect the storage modulus of PLA/TEC_0.2_ and PLA/TEC_0.4_ composite films. Further increase of the plasticizer concentration resulted in a significant decrease of the storage modulus. An increase in the TEC content increased the storage modulus obtained.

Furthermore, as shown in the plots of loss factor (tan delta) as a function of temperature in Figure 4b, increasing the concentration of TEC decreased the obtained glass transition temperature (T_g_; see Table 2) due to the property of the plasticizer to distance the polymeric chains and facilitate their movement.

### 3.4. Water/Oxygen Barrier Properties of PLA/TECx Composite Films

Table 3 and Table 4 present the obtained water vapor transmission rate (WVTR) and oxygen transmission rate (OTR) values, as well as the calculated water vapor diffusion coefficient (D_wv_) and the oxygen permeability (P_O2_) values of all PLA and PLA/TECx films. Descriptive statistics from the statistical analysis are presented in Supplementary Table S6, while the significance of the difference in the mean values of D_wv_ and P_O2_ among the tested samples is listed in Supplementary Tables S7–S9. A general result from Table S7 is that, considering the oxygen permeability coefficient, there are statistically significant differences between the mean values of all tested samples, while considering the water/vapor diffusion coefficient, the mean values of all tested samples are statistically equal.

More detailed pairwise comparisons for each tensile parameter are presented in Tables S8 and S9. Descriptive statistics are also presented graphically in Figure S10.

As shown in Table 3, the calculated D_wv_ value for PLA films is 1.40 × 10^−4^ cm^2^/s. This value is too close to the range of 1.94–2.39 × 10^−4^ cm^2^/s, which is reported in the literature for PLA films [35,45]. Even though the calculated D_wv_ values of PLA/TEC_x_ composite films are statistically different and lower compared to the WVTR and D_wv_ values of the pure PLA film, there is no statistical difference between PLA/TEC_x_ films. Nevertheless, considering Figure S10, we could say that the sample PLA/TEC_0.8_ exhibits the lower D_wv_ value. In other words, the addition of 5% *v*/*w* TEC significantly affects the water barrier properties of PLA films, but any extra addition cannot further change this property.

Table 4 provides the calculated oxygen permeability coefficient (P_O2_) value for PLA film, which is 2.63 ± 0.28 × 10^−9^ cm^2^/s. This value is one order of magnitude lower than the values given in the literature for PLA films, which ranged between 4.21 and 5.76 × 10^−8^ cm^2^/s [45]. The calculated P_O2_ absolute values of PLA/TEC_0.4_ and PLA/TEC_0.6_ composite films are lower than the calculated P_O2_ absolute values of PLA films. On the other hand, the calculated P_O2_ absolute values for the PLA/TEC_0.8_, PLA/TEC_1.0_, and PLA/TEC_1.2_ composite films are higher than the P_O2_ absolute values of PLA film. According to the statistical analysis (see Table S8), the obtained values are significantly different only for PLA/TEC_1.2_ composite films. These results suggest that the addition of TEC in 5, 10, 15, 20, 25, and 30% (*v*/*w*) does not significantly affect the oxygen barrier of obtained PLA/TEC_0.2_, PLA/TEC_0.4_, PLA/TEC_0.6_, and PLA/TEC_0.8_ films as compared to the high oxygen barrier of pure PLA film, while the addition of 30% (*v*/*w*) TEC, statistically decreases the oxygen barrier of obtained PLA/TEC_1.2_ film as compared to high oxygen barrier of pure PLA film.

### 3.5. Migration of Plasticizer

In Table 5, the mean calculated values of % weight loss of all PLA/TEC_x_ composite films as well as the pure PLA film are listed for comparison. Descriptive statistics are presented in Table S6. According to Table S7, the % weight loss of all the developed films is statistically different from each other. Table 5 indicates that the addition of 5% *v*/*w* TEC content in PLA does not change (*p* > 0.05) the % weight loss of PLA/TEC_0.2_. The % weight loss of the PLA/TEC_0.4_ composite film is statistically different compared to all other films. On the other hand, the addition of TEC content in higher amounts (i.e., 15, 20, 25, and 30% (*v*/*w*)) to the PLA polymeric matrix exhibited values of % weight loss statistically different compared to the values obtained for PLA, PLA/TEC_0.2_, and PLA/TEC_0.4_ films. Additionally, the % weight loss values for films with 15, 20, 25, and 30% (*v*/*w*) TEC content are statistically equal to each other (see Table S11). For the composite films with TEC content higher than 15% *v*/*w*, the % weight loss was approximately equal to 2. These low % weight loss values observed for the PLA/TEC_x_ composite films imply the good blending of TEC molecules with PLA chains. In addition, it must be noted that after the end of migration experiments, all the tested films preserve their initial shape and appearance. Considering that TEC is a Quantum Satis food additive (E number E1505) it is concluded that such composite films could be used as novel active packaging films for foods in pasteurization processes.

### 3.6. Antioxidant Activity of PLA/TECx Composite Films

The EC_50_ values for PLA and PLA/TECx composite films are included in Table 5. Descriptive statistics are presented in Table S6. In general, according to Table S7, all tested samples exhibited different EC_50_ mean values. More detailed pairwise statistical results are presented in Table S10.

According to the EC_50_ values given in Table 5, all PLA and PLA/TEC_x_ composite films indicated antioxidant activity. Even though some samples did not exhibit statistically significant differences considering their EC_50_ mean values, Figure S11 shows that by increasing the %(*v*/*w*) TEC content in PLA/TEC_x_ composite films, the obtained EC_50_ values decrease. Thus, the total antioxidant activity increases. Statistical analysis results showed that the EC_50_ value of PLA/TEC_0.2_ composite film is not significantly different from the obtained EC_50_ value of PLA film. The obtained EC_50_ values for all other PLA/TEC_x_ composite films were statistically significantly different than the EC_50_ value of PLA film.

### 3.7. Antibacterial Activity of PLA/TECx Composite Active Films

The antibacterial effect of the PLA/TECx composite active films is presented in Table 6.

**Figure 5:**
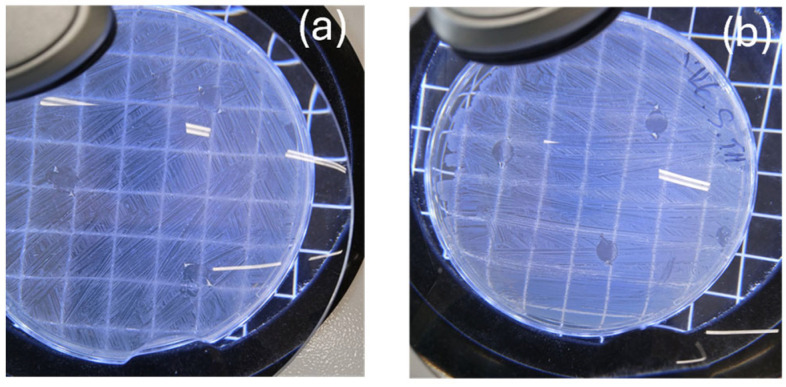
Representative images of obtained inhibition zones against (**a**) *S. aureus* and (**b**) *S. Typhimurium* of tested PLA/TEC_0.6_ films.

Figure 5 shows representative images of obtained inhibition zones against *S. aureus* and *S. Typhimurium* of PLA/TEC_0.6_ film for comparison.

The results showed that all tested films exhibited antimicrobial activity and inhibited bacterial growth only in the contact area under the film (6 mm), without any clear zone of inhibition observed around the films and beyond the contact area for both microorganisms. Moreover, *S. Typhimurium* was inhibited in all treatments with the PLA/TECx composite films, which presented strong antimicrobial activity against the pathogen (i.e., 0 out of 6 replicates with growth in the contact area of the PLA/TECx composite film sample). On the contrary, although *S. aureus* was inhibited in all treatments with the PLA/TECx composite active films, the latter presented moderate (i.e., three out of six replicates with growth in the contact area of the sample) to clear (i.e., one to two out of six replicates with growth in the contact area of the sample) antimicrobial activity in the contact area. To the best of our knowledge, the addition of TEC having a significant effect on the antibacterial activity of PLA-based composite films is for the first time reported.

### 3.8. Lipid Oxidation and Heme Iron Content of Pork Fillets

**Figure 6:**
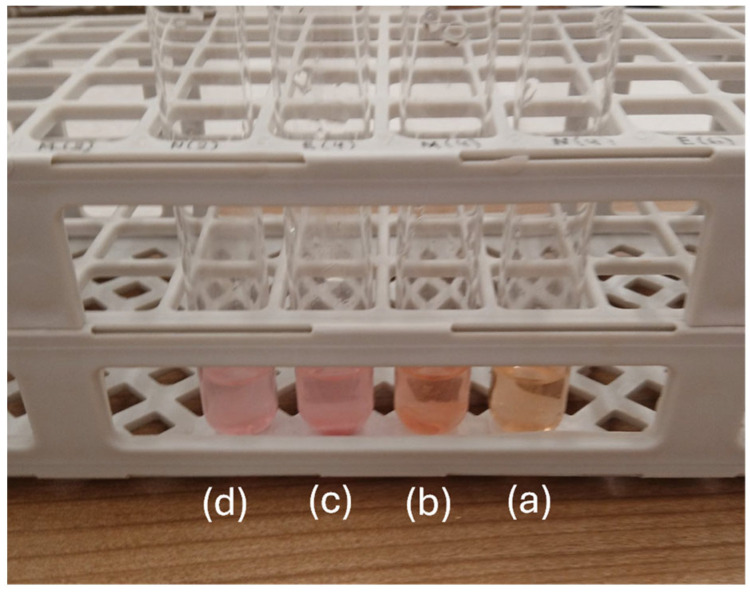
Representative TBA color changes for lipid oxidation experiment of pork fillet wrapped with PLA/TEC_0.6_ film during (a) day 0, (b) day 2, (c) day 4, and (d) day 6 of storage.

Thiobarbituric acid reactive substances (TBARS) and Heme iron content values of pork fillets wrapped with the commercial paper (Control), PLA, and PLA/TEC_0.6_ films are shown in Table 7. Representative TBA color changes for the lipid oxidation experiment of pork fillet wrapped with PLA/TEC0.6 film during (a) day 0, (b) day 2, (c) day 4, and (d) day 6 of storage are shown in Figure 6. Descriptive statistics are presented in Table S12. The results of the statistical analysis are listed in Tables S13–S15. Descriptive statistics are also presented graphically in Figure S12.

According to Table S13, only after 4 days the obtained were the TBARS values statistically different, while the heme iron values were statistically different since day 2. Considering the TBARS values shown in Table 7 and Figure S12, the trend between the control (commercial paper) and PLA films was almost the same, while after 6 days the PLA/TEC_0.6_ sample exhibited the higher resistance to the lipid oxidation process. On the other hand, from these two sources, the heme iron parameter of the PLA/TEC_0.6_ film, which indicates the delaying of lipid oxidation in pork fillets, is statistically significantly higher since day 2. This result may be the effect of the increased antioxidant activity and lower water diffusivity of the PLA/TEC_0.6_ film compared to the relevant PLA film or commercial paper. As an overall observation, we could say that pork fillets wrapped with commercial paper and PLA films had no statistically significant difference, showing the same trend throughout the storage time. For pork fillets wrapped with PLA/TEC_0.6_, film, although only on days 2 and 4 had statistically significant different heme iron values, such values were higher than the relevant of pork fillets wrapped in the Control and PLA films. Thus, PLA/TEC_0.6_ films were able to preserve the pork fillets with a higher heme iron content, which is beneficial from a nutritional point of view. In advance, there is a linear correlation between TBARS values and the heme iron content of pork fillets in accordance with previous reports [31,46].

### 3.9. Correlation of TBARS and Heme Iron

The bivariate Pearson’s correlation analysis was performed on the obtained TBARS and heme iron content values of pork fillets. The analysis results indicated that significant and positive correlations were observed between the two methods throughout the storage period. The corresponding correlations, which were determined in relation to the packaging system and storage time, are shown in Supplementary Materials (see Table S16).

### 3.10. Microbiological Changes of Pork Fillets

TVC gives a quantitative estimate of the population of microorganisms capable of forming visible colonies, such as bacteria, yeasts, and molds in a food sample. Most microorganisms present in pork fillets, either as a part of its natural microflora, or as the result of cross-contamination from other sources, are mostly aerobic microorganisms, and their population is an indicator of product microbiological quality [41]. Table 8 shows the changes in TVC of pork fillets as a function of the kind of film used and the storage time. Descriptive statistics of TVC parameters versus storage days and packaging material are presented in Table S17 and graphically in Figure S13.

As shown in Table 8, the initial value of TVC was 4.38 log cfu/g, indicating a good microbiological quality of pork meat. The upper microbiological limit for acceptable quality of foods TVC is 7 log cfu/g (ICMSF directive) [47]. According to Table S18, all samples exhibited statistically different TVC values throughout the storage time (2–6 Days). More specifically, according to Table 8 and Table S19, after day 4, the PLA/TEC_0.6_ packaging film exhibited statistically a significantly better resistance to the microorganism growth on pork fillets. The limit value (7 log cfu/g) was slightly overlapped in PLA/TEC_0.6_ packaging system on day 6, while on the same day the same limit was significantly exceeded by the commercially used wrapping paper. Overall, it is concluded that PLA/TEC_0.6_ is a promising polymeric matrix that, with the enrichment of antioxidant or bioactive substances, could succeed in extending the shelf life of pork fillets.

### 3.11. Sensory Evaluation of Pork Fillets

Sensory properties such as odor, color, and cohesion are major factors that influence consumers’ acceptance of a food product. Off odors in spoiled pork meat could be related to compounds originating from the growth of some microorganisms (e.g., dimethyl disulfide, dimethyl sulfide and propylene sulfide generated by *Pseudomonas* spp. or acetoin, diacetyl and 3-methylbutanol produced by *homofermentative LAB*, *Enterobacteriaceae*, *Brochothrix thermosphacta*) [42]. Chemical compounds, such as ammonia or amines resulting from protein breakdown and ketones and aldehydes resulting from lipid oxidation, could be responsible for the development of off-meat odors [42]. The results of the sensory evaluation of the present study are displayed in Table 9. Descriptive statistics for sensory parameters are presented in Table S12 and graphically in Figure S14.

According to the overall statistical analysis of Table S13, the cohesion of the tested samples is statistically different from day 4 and after, while the color differs on day 2, is statistically equal on day 4, and differs again significantly on day 6. The odor sensory parameter of each tested sample was statistically unequal on day 2 and after. More specifically, according to Table 9 and Tables S20–S22, it is statistically clear that PLA/TEC_0.6_ exhibited better sensory behavior, especially on day 6. This result can be related to the fact that the PLA/TEC_0.6_ packaging system succeeded in delaying the rate of lipid oxidation throughout storage time (Table 7). This finding shows that PLA/TEC_0.6_ is a promising polymer matrix that has a positive effect on sensory properties as well as lipid oxidation, heme iron content, and total viable count (TVC) properties, and with the addition of active compounds, could be used to extend the shelf life of foods [46].

## 4. Discussion

As shown, PLA/TEC composite films were successfully prepared with varying %*v*/*w* TEC content (5, 10, 15, 20, 25, and 30 mL in 100 g of PLA) via a twin-screw extrusion molding method. The shift of PLA-XRD broad peak from 15.5° to 16.2° for TEC concentrations 15% *v*/*w* and over indicates good incorporation of the two film substances in this range of concentrations. This phenomenon was confirmed by the FTIR measurements that exhibited a sharper and taller peak at 3500 cm^−1^, which suggests that blending of TEC molecules with PLA chains is more effective for PLA contents 15% *v*/*w* or higher. This result is in accordance with previous reports, which also showed the increase of plasticization effect of TEC on PLA by increasing the %wt content of TEC [26,28]. Furthermore, the present study presents new results on the effective plasticization interaction of the TEC molecule with PLA, showing for the first time via SEM images that this interaction is better for TEC addition concentrations between 15% *v*/*w* and 20% *v*/*w*. SEM images agree with the results of XRD and FTIR analysis, and we additionally conclude that the optimal concentrations of TEC are evident between 15 and 20% *v*/*w* (samples with code names PLA/TEC_0.6_ and PLA/TEC_0.8_) as the plasticizer can be better incorporated into the amorphous parts of PLA polymer matrix with complete mixing and homogeneous morphology. Mechanical and thermomechanical properties indicate the increase of flexibility and decrease of T_g_ temperature by increasing TEC content in PLA/TECx composite films. In accordance with SEM images and tests of mechanical properties, the results suggest that by increasing the TEC% *v*/*w* content higher than 20% *v*/*w*, no significant difference is achieved in the absolute values of ultimate strength (σ_uts_) and %elongation at break (%ε) values. This fact implies that the samples with 15% *v*/*w* (PLA/TEC_0.6_) and 20% *v*/*w* achieved the highest TEC molecules blending with PLA chains. Water and oxygen barrier properties suggest that the addition of TEC does not significantly affect the high water and oxygen barrier properties of PLA film except for the case of PLA/TEC film with the highest % *v*/*w* content (PLA/TEC1.2), where a reduction of oxygen barrier properties was recorded. Plasticizer test migration under pasteurization conditions showed no difference in the appearance of films after the end of the test and low TEC release up to approx. 2% weight loss for composite films with TEC content higher than 15% *v*/*w*. The antioxidant activity test showed that the obtained EC50 values increased with the increase in TEC% *v*/*w* contents. Finally, all PLA/TECx composite films exhibited significant antibacterial activity tests against *S. aureus* and *S. Typhimurium* food pathogens without release zone. Considering all the above-listed results from the physicochemical characterization of PLA/TECx composite films as well as from mechanical, thermomechanical, water/oxygen barrier, release properties, and antioxidant/antibacterial activity, it was concluded that the films with the highest blending are the films with code names PLA/TEC_0.6_ and PLA/TEC_0.8_. Considering that these two films have no significant differences in water/oxygen barrier properties and antioxidant/antibacterial activity, we choose the PLA/TEC0.6 film as the optimum film of all blends to use less TEC content as it can be used. This optimum film was used to wrap fresh pork fillets, and a shelf life experiment was conducted by monitoring the lipid oxidation values, the heme iron content values, the TVC values, and color, odor and cohesion values during the storage period at 4 °C. The shelf life experiment showed that PLA/TEC0.6 film has positive effect on sensory properties, as much as in lipid oxidation, heme iron content and total viable count (TVC), of fresh pork fillets meat compared to packaging film used in commerce.

Thus, it is concluded that PLA/TEC_0.6_ composite film is a promising matrix for flexible active packaging applications. Its flexibility, good water/oxygen barrier, and antioxidant/antibacterial activity make it an ideal candidate for the incorporation of bioactive nanocarriers and the development of nanocomposite flexible active packaging films.

## 5. Conclusions

The primary target of this work was to develop a novel self-healable, food-active packaging film using environmentally friendly biodegradable materials. Similar scientific studies are reported in [25,26,27] with similar results on mechanical and barrier properties but with no antioxidant, antimicrobial, or in-vivo shelf-life experiments. Furthermore, no self-healing properties were reported in these works. The PLA/TEC_0.6_ material (i.e., around 15% *v*/*w* TEC composition in PLA polymeric matrix) seems to be the optimum product capable of fulfilling the initial target. Visual tests for the self-healing capability of the novel films showed that in 120 s, the repair procedure was integrated, and a completely healed material was achieved. XRD and FTIR measurements indicated good incorporation of the two substances for a maximum composition of 20% *v*/*w* (i.e., PLA/TEC_0.8_ material), which was also confirmed via the SEM technique. Tensile measurements showed that the addition of plasticizer TEC in concentrations greater than 15% *v*/*w* in the PLA polymeric matrix, decreased drastically the Young Modulus (E) and the ultimate strength (σ_uts_) and increased the elongation at break (ε%). This means that over this concentration, the elasticity increased, but the strength decreased. At this point, the self-healing property can contribute significantly to the overall behavior of the final film. Finally, no significant changes were observed for water/oxygen barrier properties, migration percentages, and antimicrobial activity compared to the initial pure PLA film, while the antioxidant, lipid oxidation, and deterioration behavior for active food packaging applications improved drastically by TEC addition concentrations up to around 15%. The same increased resistant behavior was observed for sensory parameters (i.e., odor, color, and cohesion) by the addition of TEC component.

As an overall conclusion, we could say that the PLA/TEC film that was developed in this work could become an advanced, biodegradable, “green” food packaging that can extend the food shelf life through its antioxidant, antimicrobial, increased barrier, and self-healing properties.

## Figures and Tables

**Table 1:** Sample names, PLA, TEC contents, operating temperatures, and operating speeds of the twin extruder used for the development of all PLA/TEC composite blends.

Sample Code	PLA (g)	TEC mL (*v*/*w*)	Twin Extruder Processing Conditions		
			Temperature (°C)	Speed (rpm)	Time (min)
PLA	4	-	180	120	5
PLA/TEC_0.2_	4	0.2 (5 mL/100 g)	180	120	5
PLA/TEC_0.4_	4	0.4 (10 mL/100 g)	180	120	5
PLA/TEC_0.6_	4	0.6 (15 mL/100 g)	180	120	5
PLA/TEC_0.8_	4	0.8 (20 mL/100 g)	180	120	5
PLA/TEC_1.0_	4	1.0 (25 mL/100 g)	180	120	5
PLA/TEC_1.2_	4	1.2 (30 mL/100 g)	180	120	5

**Table 2:** Mean values (±Standard Deviation) of modulus of elasticity (E), tensile strength (σ_uts_), and % elongation at break (ε_b_) of all tested PLA/TEC_x_ films as well as pure PLA film. The calculated from DMA plots T_g_ values are also listed for all tested PLA/TEC_x_ films as well as PLA film.

Sample Code	Elastic Modulus, E (MPa)	Ultim. Strength σ_uts_ (MPa)	% Elongation at Break (ε%)	T_g_ (°C)
PLA	3102.0 ± 1221.9 ^a^	90.9 ± 24.81 ^a^	5.0 ± 1.0 ^a^	78
PLA/TEC_0.2_	2832.6 ± 565.2 ^a^	51.1 ± 13.7 ^b^	3.4 ± 0.4 ^b^	64
PLA/TEC_0.4_	2675.8 ± 484.28 ^a^	50.5 ± 23.1 ^b^	4.0 ± 2.2 ^a,b^	55
PLA/TEC_0.6_	1438.8 ± 321.55 ^b^	29.4 ± 5.7 ^c^	52.6 ± 48.9 ^c,d^	48
PLA/TEC_0.8_	687.2 ± 225.7 ^c^	17.5 ± 3.1 ^d^	58.7 ± 6.8 ^c^	47
PLA/TEC_1.0_	375.8 ± 129.2 ^c,d^	18.9 ± 4.5 ^b,c,d^	68.3 ± 53.2 ^c^	34
PLA/TEC_1.2_	239.0 ± 53.03 ^d^	20.4 ± 3.7 ^d^	122.3 ± 22.0 ^d^	33

^a–d^: Different letters in each column indicate statistically significant differences at the confidence level *p* < 0.05.

**Table 3:** Film thickness, water vapor transmission rate (WVTR), and water diffusivity (D_wv_) values of pure PLA film and all PLA/TECx films.

Sample	Film Thickness (mm)	WVTR ×10^−7^ (g/cm^2^.s)	Diffusion Coefficient ×10^−4^ (cm^2^/s)
PLA	0.098 ± 0.021	6.522 ± 1.092	1.40 ± 0.09 ^a^
PLA/TEC_0.2_	0.089 ± 0.030	4.648 ± 1.146	0.91 ± 0.21 ^b^
PLA/TEC_0.4_	0.088 ± 0.026	3.958 ± 0.996	0.76 ± 0.09 ^b^
PLA/TEC_0.6_	0.102 ± 0.020	5.078 ± 1.763	1.15 ± 0.35 ^a,b^
PLA/TEC_0.8_	0.081 ± 0.0171	4.091 ± 0.554	0.76 ± 0.21 ^b^
PLA/TEC_1.0_	0.078 ± 0.024	6.093 ± 1.340	1.04 ± 0.28 ^a,b^
PLA/TEC_1.2_	0.099 ± 0.010	4.500 ± 1.705	1.04 ± 0.35 ^a,b^

^a,b^: Different letters in each column indicate statistically significant differences at the confidence level *p* < 0.05.

**Table 4:** Film thickness, oxygen transmission rate (OTR), and oxygen permeation coefficient (P_O2_) values of PLA and PLA/TEC_x_ films.

Sample	Average Film Thickness (mm)	OTR [mL/(m^2^ × Day)]	P_O2_ ×10^−9^ (cm^2^/s)
PLA	0.110 ± 0.002	189.5 ± 17.7	2.64 ± 0.28 ^a^
PLA/TEC_0.2_	0.111 ± 0.016	203.0 ± 59.4	2.66 ± 1.14 ^a,b^
PLA/TEC_0.4_	0.060 ± 0.004	339.0 ± 70.7	2.38 ± 0.65 ^a^
PLA/TEC_0.6_	0.063 ± 0.012	298.0 ± 94.8	2.24 ± 1.11 ^a,b^
PLA/TEC_0.8_	0.056 ± 0.004	503.0 ± 56.6	3.29 ± 0.61 ^a,b^
PLA/TEC_1.0_	0.087 ± 0.002	317.0 ± 5.7	3.27 ± 0.02 ^b^
PLA/TEC_1.2_	0.087 ± 0.002	489.0 ± 2.8	4.62 ± 0.03 ^c^

^a–c^: Different letters in each column indicate statistically significant differences at the confidence level *p* < 0.05.

**Table 5:** % weight loss values and EC_50_ values and standard deviation of PLA and PLA/TECx composite films.

Sample Code	% Weight Loss	EC_50_
PLA	0.06 ± 0.05 ^a^	571.4 ± 65.5 ^a^
PLA/TEC_0.2_	0.10 ± 0.10 ^a^	532.0 ± 106.9 ^a^
PLA/TEC_0.4_	0.66 ± 0.19 ^b^	330.8 ± 46.7 ^b^
PLA/TEC_0.6_	1.99 ± 0.66 ^c^	212.6 ± 12.1 ^c^
PLA/TEC_0.8_	2.60 ± 0.86 ^c^	220.5 ± 51.2 ^c,d^
PLA/TEC_1.0_	2.00 ± 0.47 ^c^	178.0 ± 10.3 ^d^
PLA/TEC_1.2_	2.29 ± 0.56 ^c^	101.8 ± 4.5 ^e^

^a–e^: Different letters in each column indicate statistically significant differences at the confidence level *p* < 0.05.

**Table 6:** Antimicrobial activity of PLA/TECx composite films against the foodborne pathogenic bacteria *S. aureus* and *S. Typhimurium*.

Sample Code	Replicates with Growth in the Contact Area of Sample/Total Replicates (6)	
	*S. aureus*	*S. Typhimurium*
PLA	3/6 ^b^	0/6 ^a^
PLA/TEC_0.2_	4/6 ^c^	0/6 ^a^
PLA/TEC_0.4_	4/6 ^c^	0/6 ^a^
PLA/TEC_0.6_	3/6 ^b^	0/6 ^a^
PLA/TEC_0.8_	3/6 ^b^	0/6 ^a^
PLA/TEC_1.0_	4/6 ^c^	0/6 ^a^
PLA/TEC_1.2_	3/6 ^b^	0/6 ^a^

^a^ 0/6: growth in 6 replicates, strong antimicrobial activity, ^b^ 3/6: growth in 6 replicates, moderate antimicrobial activity, ^c^ 4/6 or 5/6: growth in 6 replicates, weak antimicrobial activity. No samples were found with 1/6 or 2/6: growth in 6 replicates, clear antimicrobial activity, or 6/6: growth in 6 replicates, no antimicrobial activity.

**Table 7:** TBARS and heme iron content values of pork fillets with respect to storage time.

Sample Code	Day 0	Day 2	Day 4	Day 6
	TBARS (mg/kg)			
CONTROL	0.46 ± 0.01 ^a^	0.59 ± 0.02 ^a^	0.75± 0.02 ^a^	0.81 ± 0.02 ^a^
PLA	0.46 ± 0.01 ^a^	0.58 ± 0.01 ^a^	0.71 ± 0.02 ^b^	0.80 ± 0.01 ^a^
PLATEC_0.6_	0.46 ± 0.01 ^a^	0.55 ± 0.01 ^b^	0.66 ± 0.01 ^c^	0.76 ± 0.01 ^b^
	**Heme iron (μg/g)**			
CONTROL	7.66 ± 0.12 ^a^	6.26 ± 0.36 ^a^	5.52 ± 0.18 ^a^	4.70 ± 0.33 ^a,b^
PLA	7.66 ± 0.12 ^a^	6.74 ± 0.21 ^a^	5.72 ± 0.12 ^a^	4.64 ± 0.19 ^a^
PLATEC_0.6_	7.66 ± 0.12 ^a^	7.12 ± 0.12 ^b^	6.10 ± 0.21 ^b^	5.20 ± 0.27 ^b^

^a,b^: Different letters in each column indicate statistically significant differences at the confidence level *p* < 0.05.

**Table 8:** TVC values of pork fillets wrapped with the CONTROL, PLA, and PLA/TEC_0.6_ films with respect to storage time.

Sample Code	logCFU/g			
	Day 0	Day 2	Day 4	Day 6
CONTROL	4.38 ± 0.03 ^a^	5.56 ± 0.13 ^a^	6.84 ± 0.06 ^a^	8.04 ± 0.01 ^a^
PLA	4.38 ± 0.03 ^a^	5.27 ± 0.16 ^a,b^	6.48 ± 0.06 ^b^	7.51 ± 0.04 ^b^
PLATEC_0.6_	4.38 ± 0.03 ^a^	5.08 ± 0.04 ^b^	6.31 ± 0.07 ^c^	7.36 ± 0.05 ^c^

^a–c^: Different letters in each column indicate statistically significant differences at the confidence level *p* < 0.05.

**Table 9:** Odor, color, and cohesion scores of wrapped pork fillets during storage at 4 ± 1 °C.

Sample Name	Day 0	Day 2	Day 4	Day 6
**Odor**				
CONTROL	5.00 ± 0.00 ^a^	4.32 ± 0.24 ^a^	3.94 ± 0.23 ^a^	3.50 ± 0.10 ^a^
PLA	5.00 ± 0.00 ^a^	4.55 ± 0.14 ^a,b^	4.22 ± 0.27 ^a,b^	3.60 ± 0.20 ^a^
PLA/TEC_0.6_	5.00 ± 0.00 ^a^	4.71 ± 0.14 ^b^	4.50 ± 0.15 ^b^	4.05 ± 0.16 ^b^
**Color**				
CONTROL	5.00 ± 0.00 ^a^	4.30 ± 0.20 ^a^	3.98 ± 0.29 ^a^	2.85 ± 0.15 ^a^
PLA	5.00 ± 0.00 ^a^	4.50 ± 0.10 ^a,b^	4.12 ± 0.33 ^a^	3.50 ± 0.16 ^b^
PLA/TEC_0.6_	5.00 ± 0.00 ^a^	4.60 ± 0.05 ^b^	4.35 ± 0.27 ^a^	4.02 ± 0.19 ^c^
**Cohesion**				
	**Day 0**	**Day 2**	**Day 4**	**Day 6**
CONTROL	5.00 ± 0.00 ^a^	4.48 ± 0.29 ^a^	3.80 ± 0.20 ^a^	2.84 ± 0.11 ^a^
PLA	5.00 ± 0.00 ^a^	4.28 ± 0.40 ^a^	4.10 ± 0.25 ^a,b^	3.32 ± 0.28 ^b^
PLA/TEC_0.6_	5.00 ± 0.00 ^a^	4.46 ± 0.27 ^a^	4.32 ± 0.11 ^b^	3.94 ± 0.18 ^c^

^a–c^: Different letters in each column indicate statistically significant differences at the confidence level *p* < 0.05.

## Data Availability

Data are contained within the article and Supplementary Materials.

## Supplementary Materials

The following supporting information can be downloaded at: https://www.mdpi.com/article/10.3390/polym16081130/s1, Figure S1: Equations for the determination of EC50 in PLA films, Figure S2: Equations for the determination of EC50 in PLA/TEC0.2 films, Figure S3: Equations for the determination of EC50 in PLA/TEC0.4 films, Figure S4: Equations for the determination of EC50 in PLA/TEC0.6 films, Figure S5: Equations for the determination of EC50 in PLA/TEC0.8 films, Figure S6: Equations for the determination of EC50 in PLA/TEC1.0 films, Figure S7: Equations for the determination of EC50 in PLA/TEC1.2 films, Figure S8: SEM surface images of (a) PLA, (b) PLA/TEC0.2, (c) PLA/TEC0.4, (d) PLA/TEC0.6, (e) PLA/TEC0.8, (f) PLA/TEC1.0, (g) PLA/TEC1.2 films, Figure S9: Graphical presentation of Table 2 statistical values, Figure S10: Graphical presentation of descriptive statistics for PO2, and Dwv properties, Figure S11: Graphical presentation of descriptive statistics for plasticizer migration (% weight loss), and antioxidant activity (EC50) properties, Figure S12: Graphical presentation of descriptive statistics for TBARS and Heme Iron indicators, Figure S13: Graphical presentation of descriptive statistics for TVC experimental measurements, Figure S14: Graphical presentation of descriptive statistics for sensory indicator values, Table S1: Descriptive statistics for Young Modulus (E), Ultimate strength (σuts), elongation at break (ε%), Table S2: Overall mean values significant difference hypothesis test using Median Test results for E,σuts, ε%, Table S3: Paiwise mean values significant difference hypothesis test using Median Test results for Young Modulus E, Table S4: Pairwise mean values significant difference hypothesis test using Median Test results for Ultimate Strength σuts., Table S5: Pairwise mean values significant difference hypothesis test using Median Test results for elongation at break ε%., Table S6: Descriptive statistics for Oxygen Permeability Coefficient (PO2), Water/Vapor Diffusion Coefficient (Dwv), EC50, %weight loss, Table S7: Overall mean values significant difference hypothesis test using Median Test results for E,σuts, ε%, Table S8: Pairwise mean values significant difference hypothesis test using Median Test results for PO2., Table S9: Pairwise mean values significant difference hypothesis test using Median Test results for Dwv., Table S10: Pairwise mean values significant difference hypothesis test using Median Test results for EC50., Table S11: Pairwise mean values significant difference hypothesis test using Median Test results for %weight loss., Table S12: Description statistics of cohesion, color, heme-iron, odor, and TBA from DAY 0 to DAY 6, Table S13: Overall mean values significant difference hypothesis test using Median Test results for cohesion, color, heme-iron, odor, and TBARS parameters at DAY 0, DAY 2, DAY 4, and DAY 6., Table S14: Pairwise mean values significant difference hypothesis test using Median Test results for TBARS parameter at DAY 0, DAY 2, DAY 4, and DAY 6., Table S15: Pairwise mean values significant difference hypothesis test using Median Test results for Heme-Iron parameter at DAY 0, DAY 2, DAY 4, and DAY 6., Table S16: Correlations between thiobarbituric acid reactive substances (TBARS) and Heme iron content., Table S17: Description statistics of TVC from DAY 0 to DAY 6, Table S18: Overall mean values significant difference hypothesis test using Median Test results for TVC parameter at DAY 0, DAY 2, DAY 4, and DAY 6., Table S19: Pairwise mean values significant difference hypothesis test using Median Test results for TVC parameter at DAY 0, DAY 2, DAY 4, and DAY 6., Table S20: Pairwise mean values significant difference hypothesis test using Median Test results for COHESION parameter at DAY 2, DAY 4, and DAY 6., Table S21: Pairwise mean values significant difference hypothesis test using Median Test results for COLOR parameter at DAY 2, DAY 4, and DAY 6., Table S22: Pairwise mean values significant difference hypothesis test using Median Test results for ODOR parameter at DAY 2, DAY 4, and DAY 6., video_s1: self-healing of PLA/TEC_0.6_, video_s2: thermoresponsive self-healing of PLA/TEC_0.6_.
