# Supplementary material for: Νovel Polylactic Acid/Tetraethyl Citrate Self-Healable Active Packaging Films Applied to Pork Fillets’ Shelf-Life Extension
Source: Polymers (Basel). 2024 Apr 17;16(8):1130. doi: 10.3390/polym16081130 (PMC11054538; doi:10.3390/polym16081130)
Supplement: Supplementary file 1 [file polymers-16-01130-s001.zip › polymers-2951486-supplementary.pdf]

# Supplementary Material

**Figure S1:** Equations for the determination of $EC_{50}$ in PLA films.

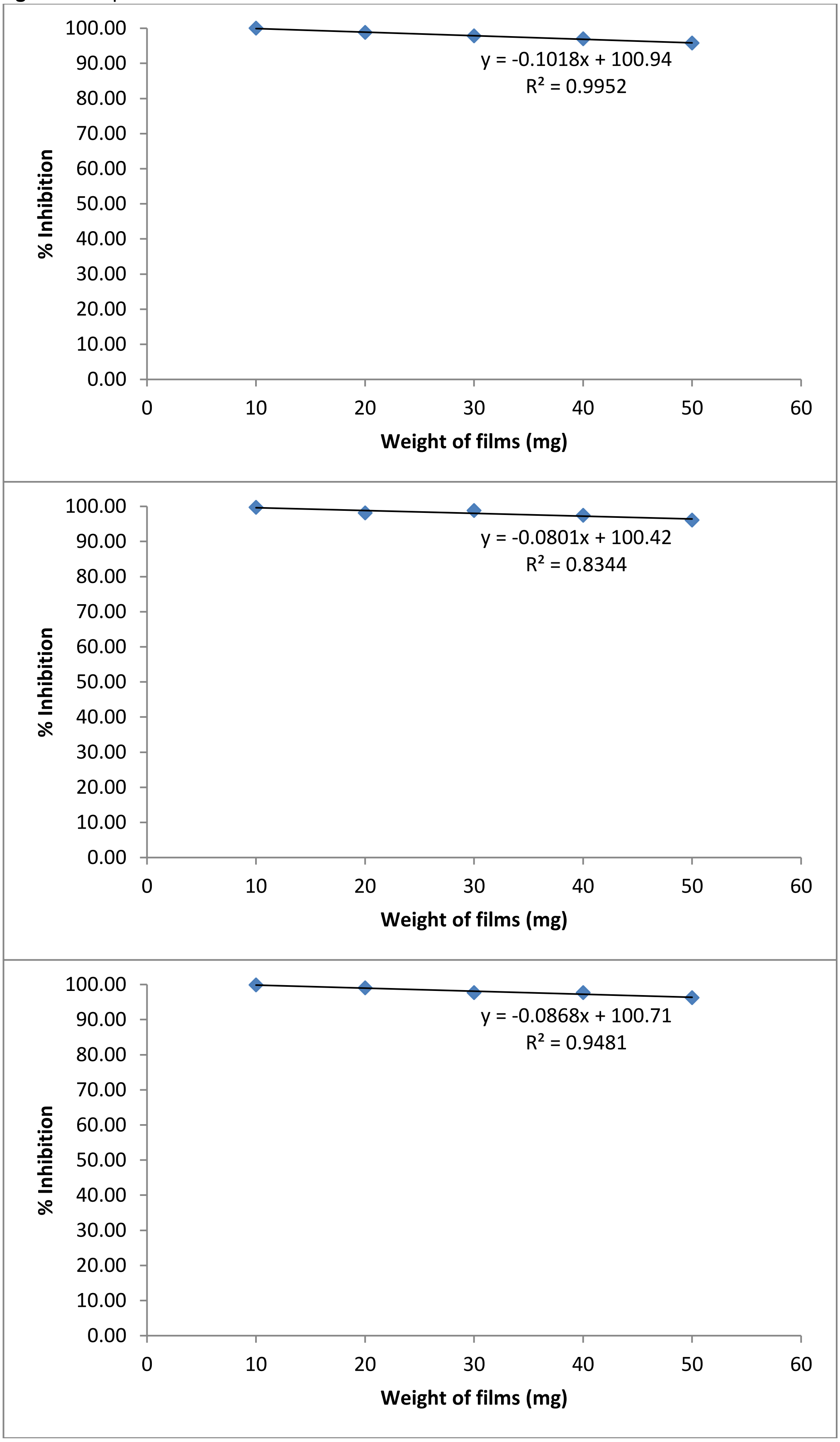

**Figure S2:** Equations for the determination of $EC_{50}$ in PLA/TEC0.2 films.

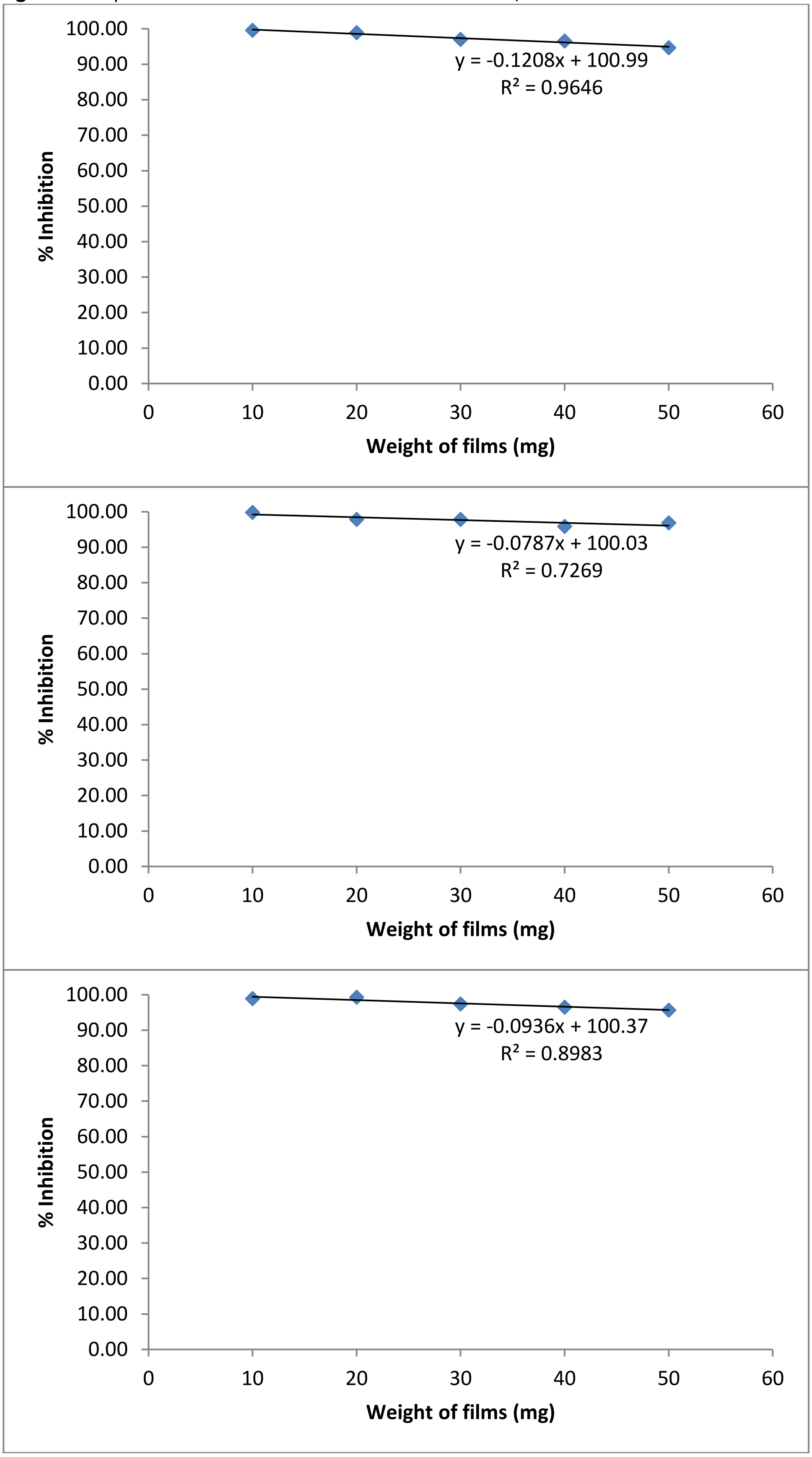

**Figure S3:** Equations for the determination of $EC_{50}$ in PLA/TEC0.4 films.

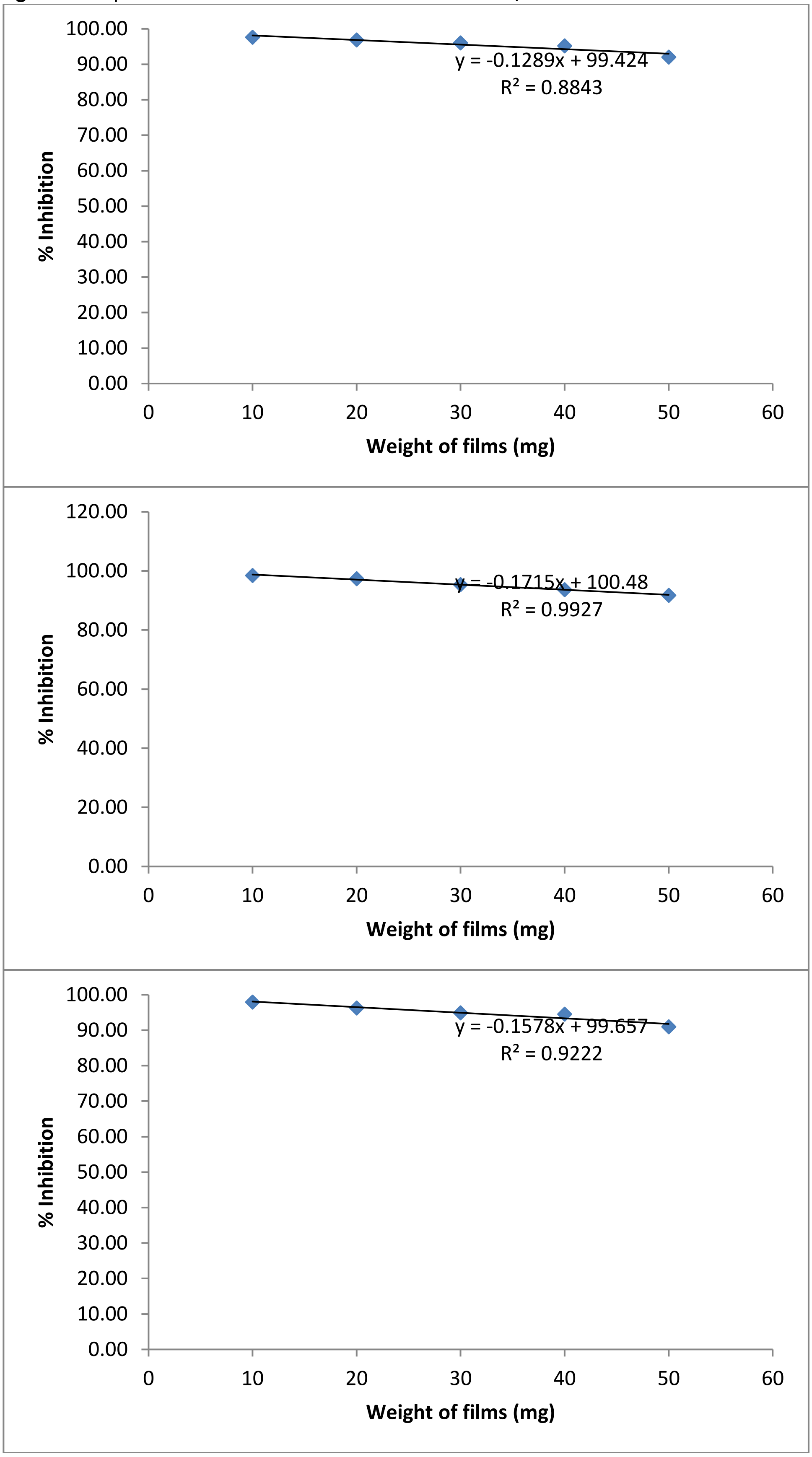

**Figure S4:** Equations for the determination of $EC_{50}$ in PLA/TEC0.6 films.

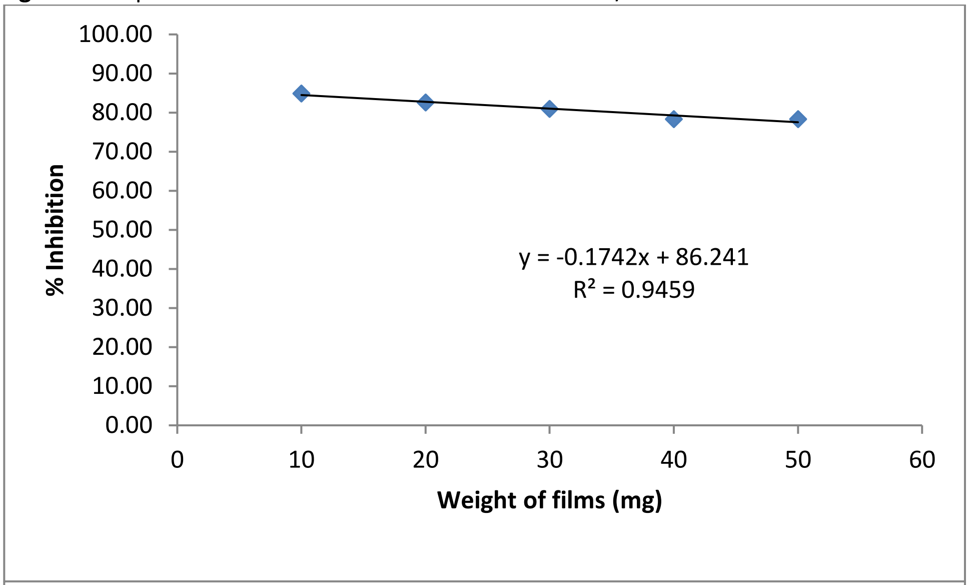


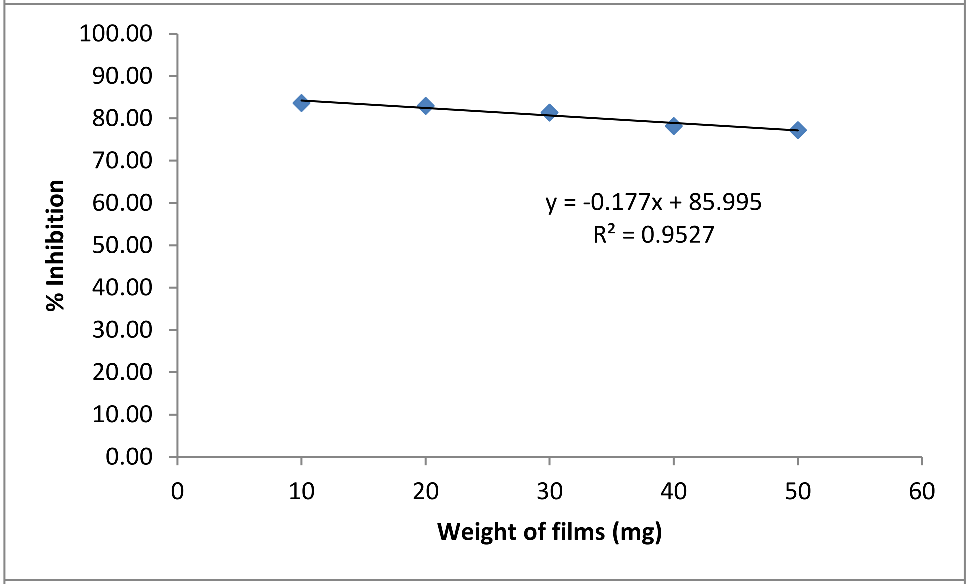


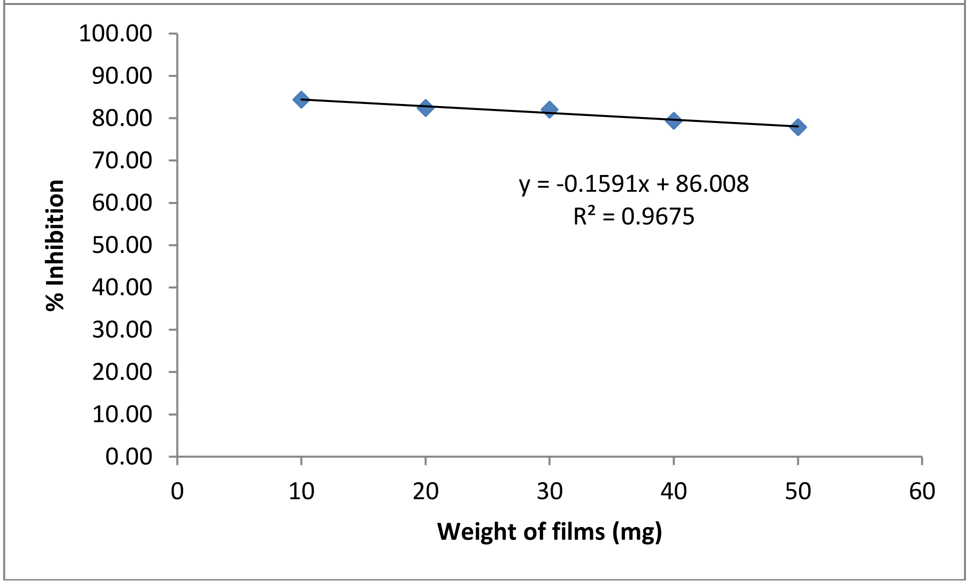

**Figure S5:** Equations for the determination of $EC_{50}$ in PLA/TEC0.8 films.

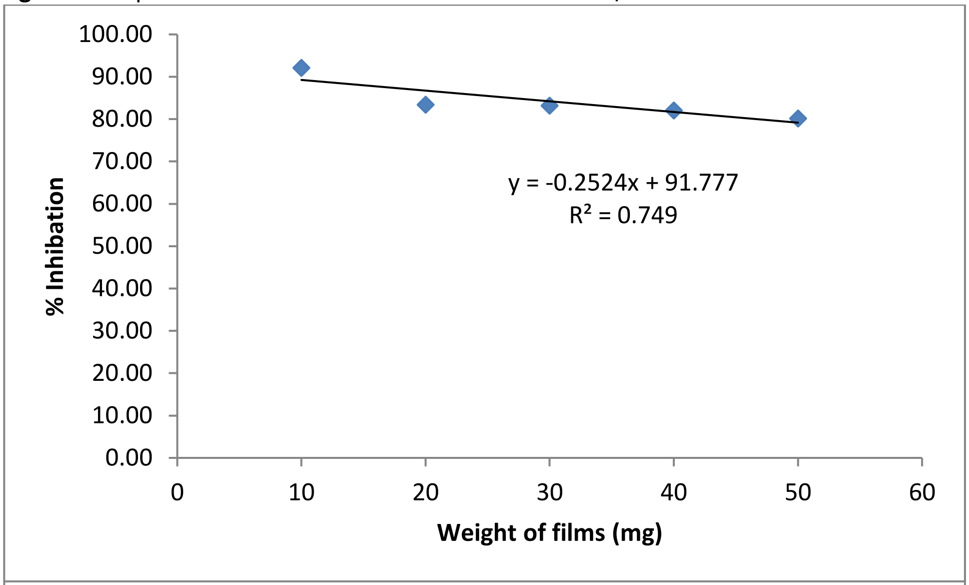


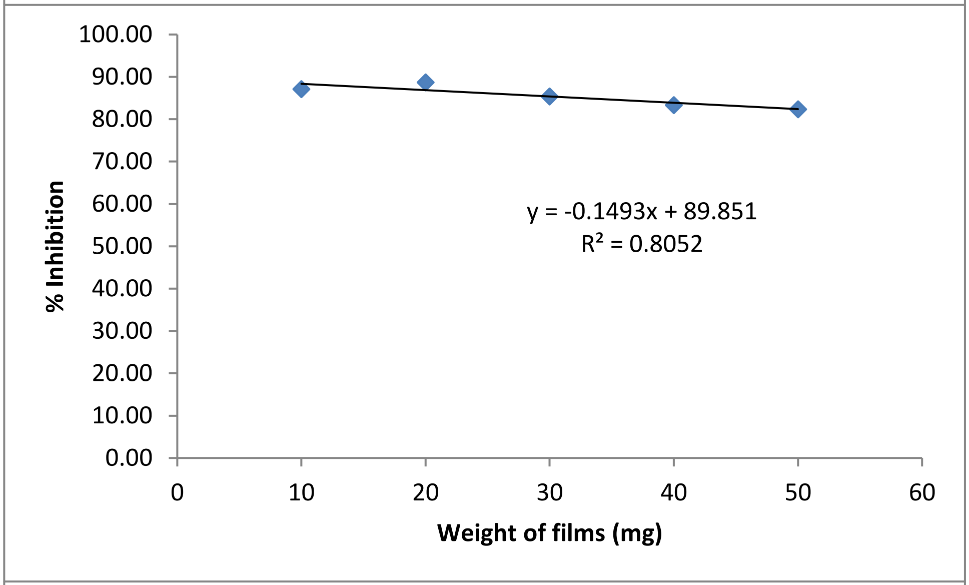


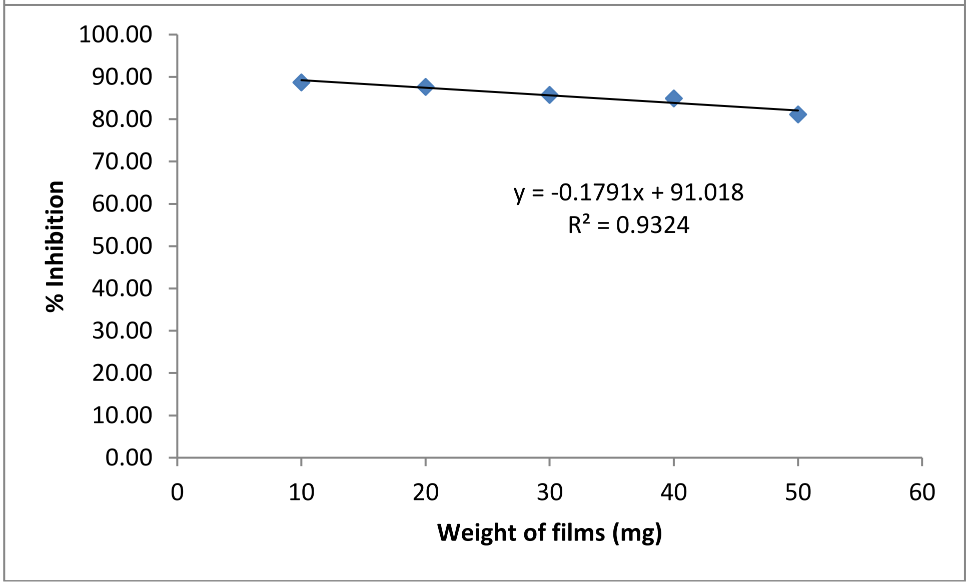

**Figure S6:** Equations for the determination of $EC_{50}$ in PLA/TEC1.0 films.

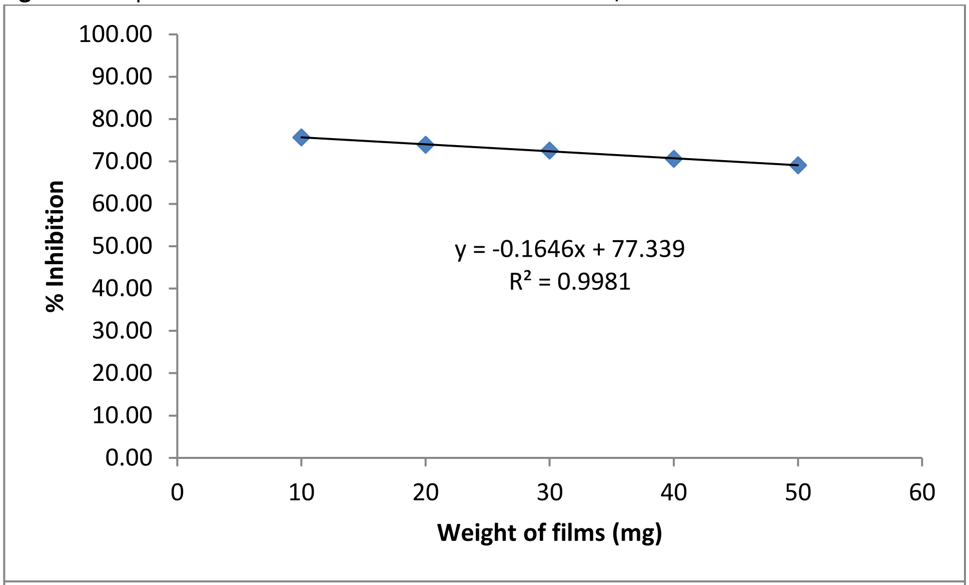


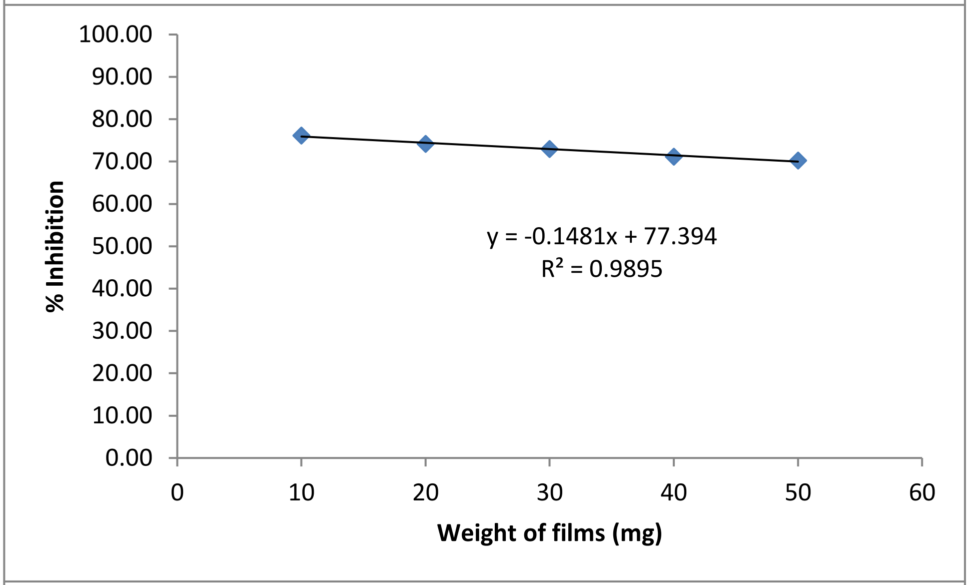


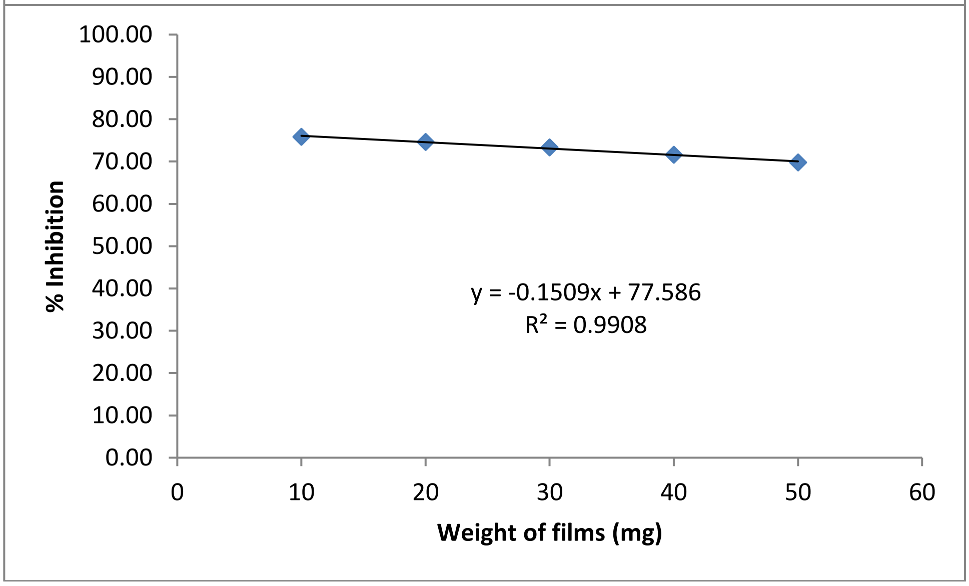

**Figure S7:** Equations for the determination of $EC_{50}$ in PLA/TEC1.2 films.

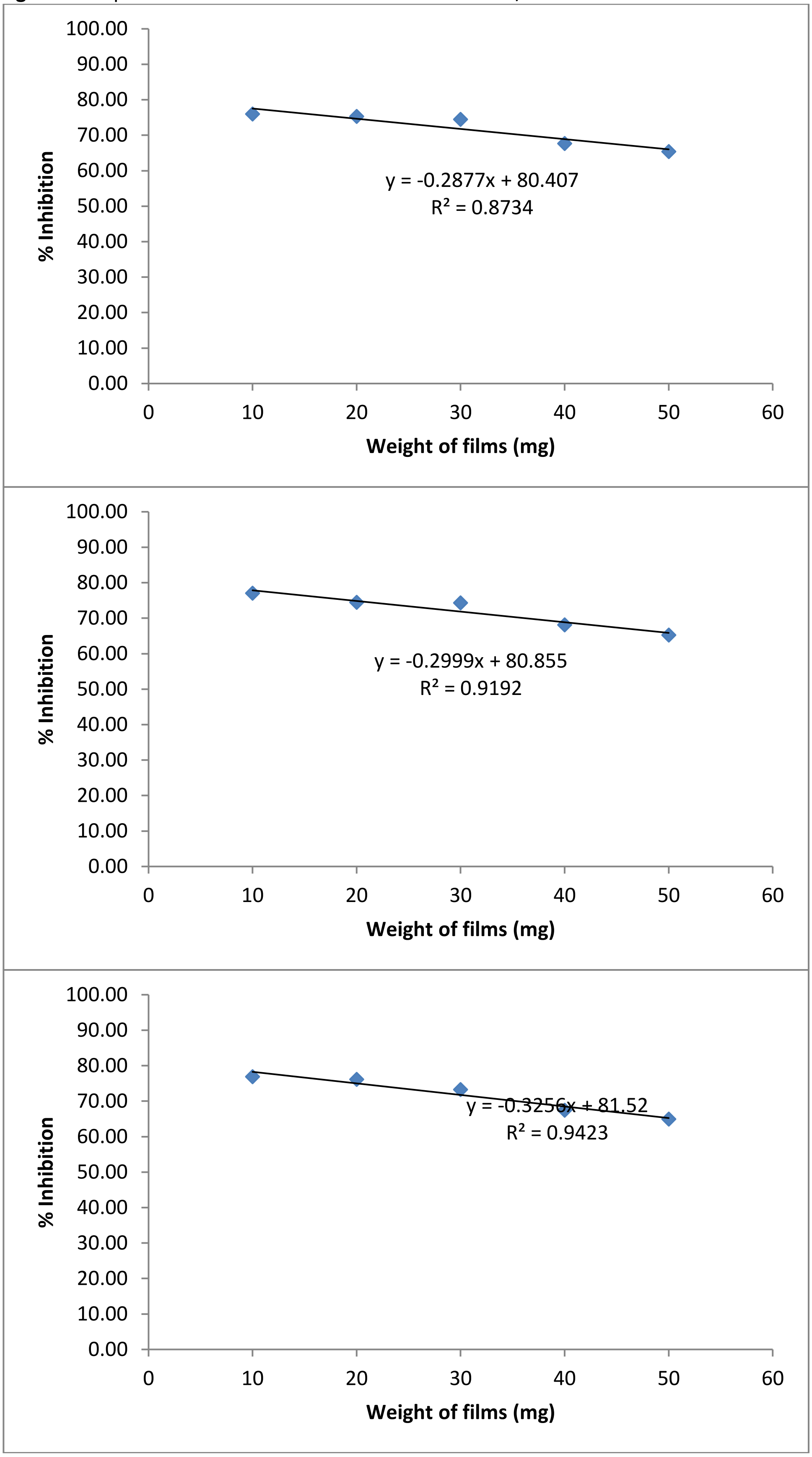

**Figure S8.** SEM surface images of (a) PLA, (b) PLA/$TEC_{0.2}$, (c) PLA/$TEC_{0.4}$, (d) PLA/$TEC_{0.6}$, (e) PLA/$TEC_{0.8}$, (f) PLA/$TEC_{1.0}$, (g) PLA/$TEC_{1.2}$ films.

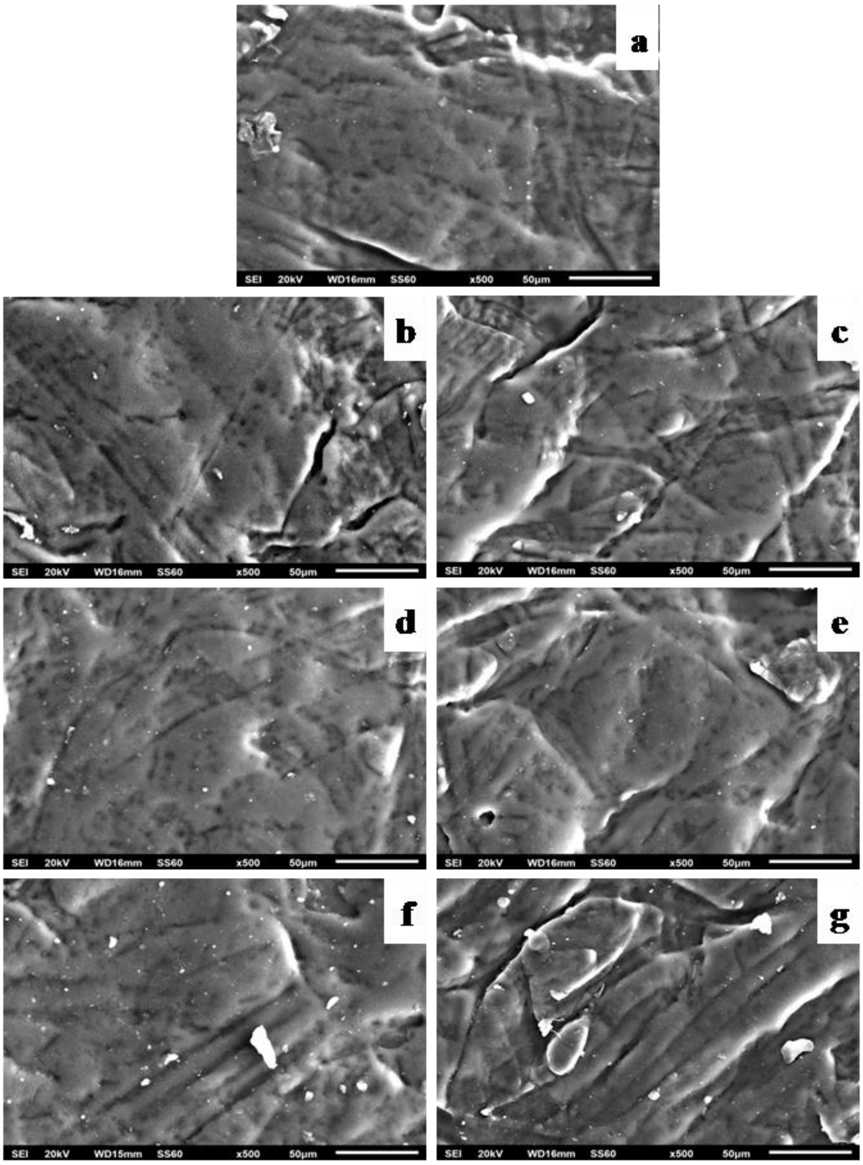


**Figure S9.** Graphical presentation of Table 2 statistical values.

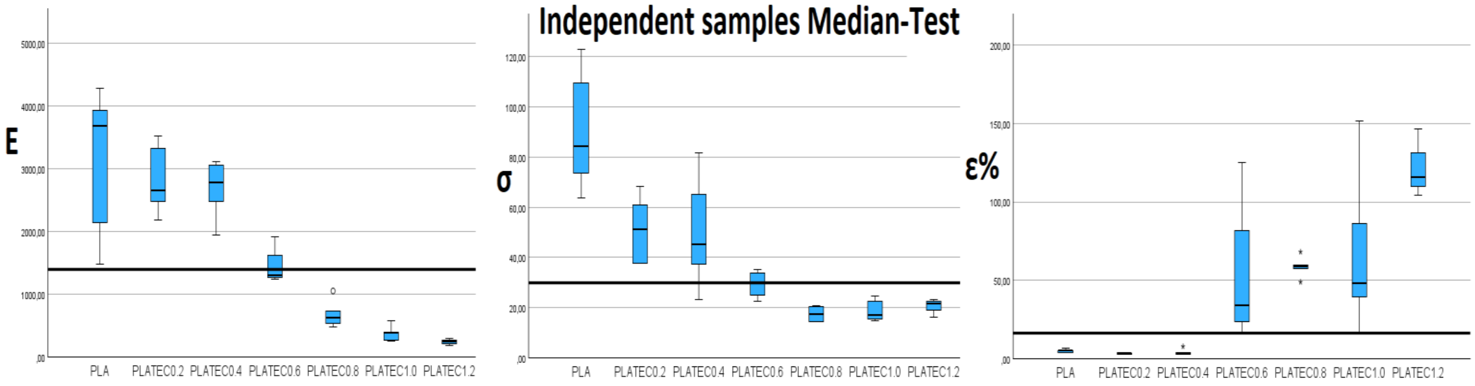

**Figure S10.** Graphical presentation of descriptive statistics for $P_{O2}$, and $D_{wv}$ properties.

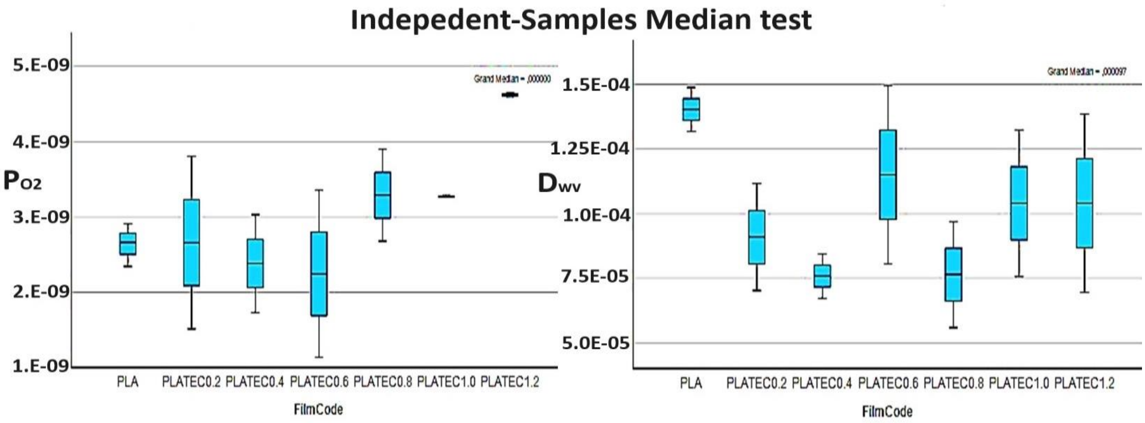


**Figure S11.** Graphical presentation of descriptive statistics for plasticizer migration (% weight loss), and antioxidant activity ($EC_{50}$) properties.

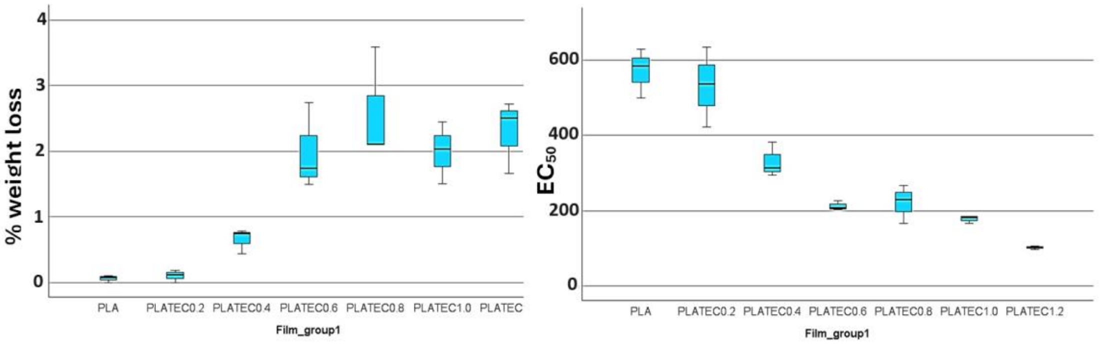


**Figure S12.** Graphical presentation of descriptive statistics for TBARS and Heme Iron indicators.

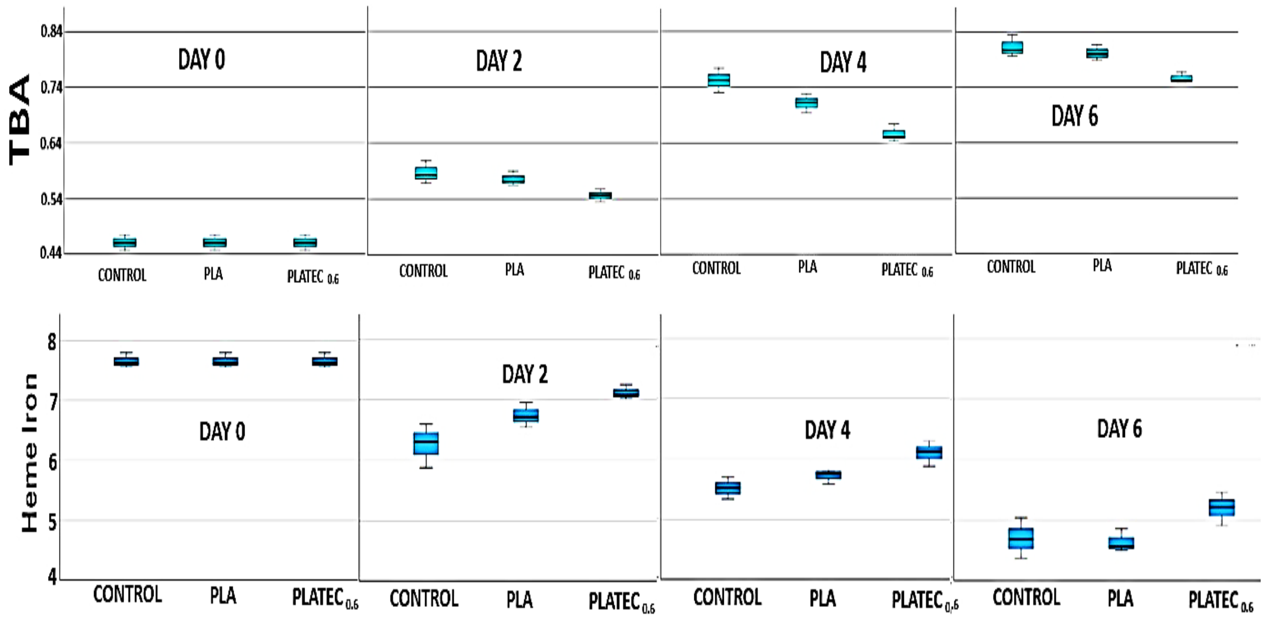

**Figure S13.** Graphical presentation of descriptive statistics for TVC experimental measurements

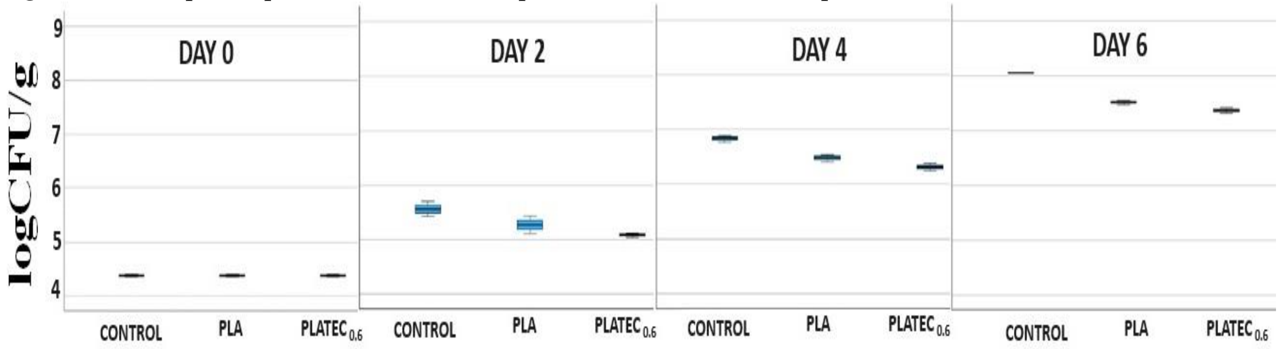


**Figure S14.** Graphical presentation of descriptive statistics for sensory indicator values.

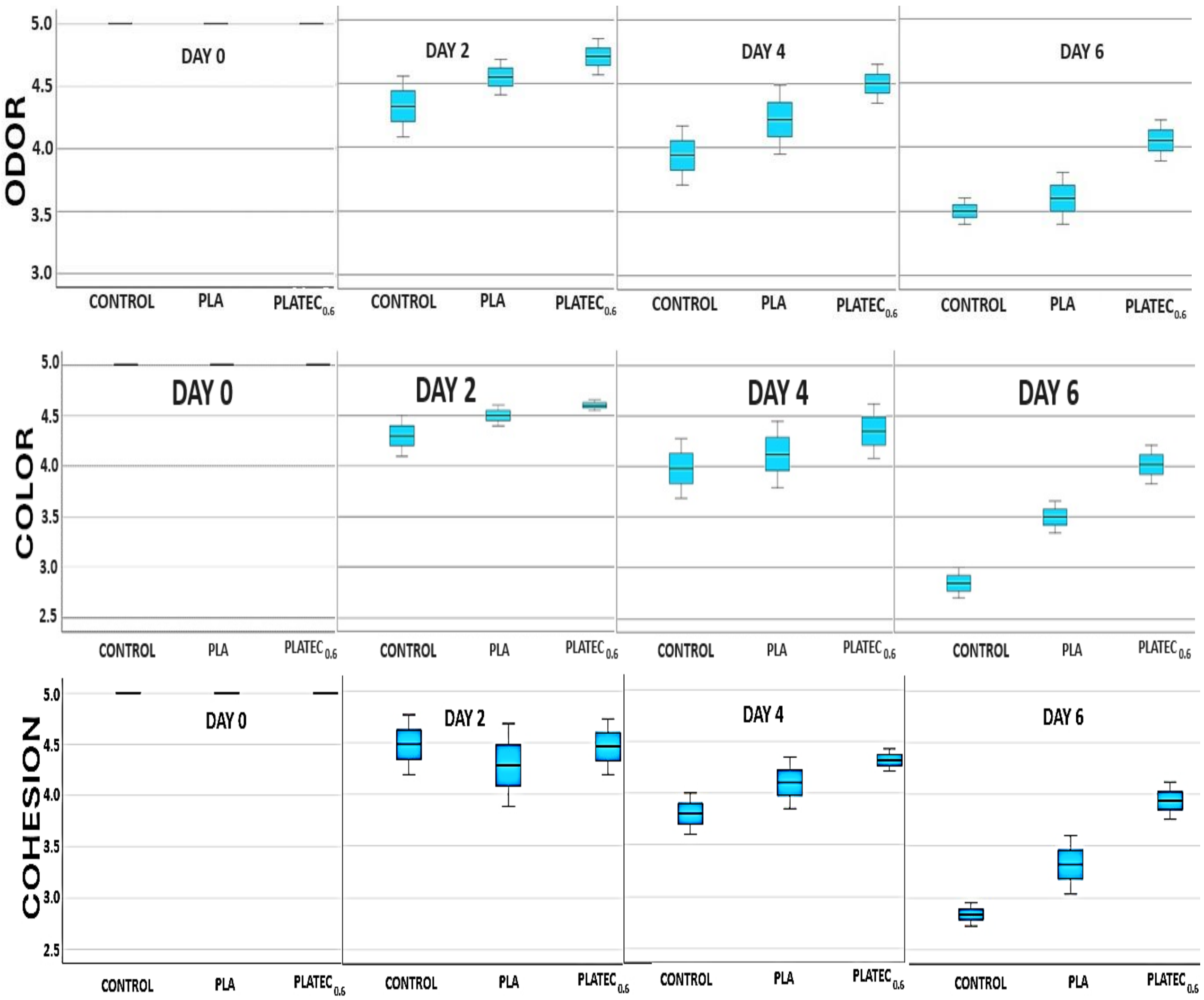

**Table S1:** Descriptive statistics for Young Modulus (E), Ultimate strength ($\sigma_{uts}$), elongation at break (ε%)

**Descriptives**

| | Film_group2 | | | Statistic | Std. Error |
|---|---|---|---|---|---|
| E | PLA | Mean | | 3102.0400 | 546.42919 |
| | | 95% Confidence Interval for Mean | Lower Bound | 1584.9094 | |
| | | | Upper Bound | 4619.1706 | |
| | | 5% Trimmed Mean | | 3126.8778 | |
| | | Median | | 3689.3000 | |
| | | Variance | | 1492924.273 | |
| | | Std. Deviation | | 1221.85280 | |
| | | Minimum | | 1480.00 | |
| | | Maximum | | 4277.00 | |
| | | Range | | 2797.00 | |
| | | Interquartile Range | | 2294.45 | |
| | | Skewness | | -.635 | .913 |
| | | Kurtosis | | -2.233 | 2.000 |
| | PLATEC0.2 | Mean | | 2832.5600 | 252.76117 |
| | | 95% Confidence Interval for Mean | Lower Bound | 2130.7825 | |
| | | | Upper Bound | 3534.3375 | |
| | | 5% Trimmed Mean | | 2830.3167 | |
| | | Median | | 2655.1000 | |
| | | Variance | | 319441.033 | |
| | | Std. Deviation | | 565.19115 | |
| | | Minimum | | 2187.00 | |
| | | Maximum | | 3518.50 | |
| | | Range | | 1331.50 | |
| | | Interquartile Range | | 1083.85 | |
| | | Skewness | | .278 | .913 |
| | | Kurtosis | | -2.279 | 2.000 |
| | PLATEC0.4 | Mean | | 2675.7800 | 216.57645 |
| | | 95% Confidence Interval for Mean | Lower Bound | 2074.4674 | |
| | | | Upper Bound | 3277.0926 | |
| | | 5% Trimmed Mean | | 2692.2333 | |
| | | Median | | 2785.5000 | |
| | | Variance | | 234526.792 | |
| | | Std. Deviation | | 484.27966 | |
| | | Minimum | | 1939.00 | |
| | | Maximum | | 3116.40 | |
| | | Range | | 1177.40 | |
| | | Interquartile Range | | 881.70 | |
| | | Skewness | | -.967 | .913 |
| | | Kurtosis | | .099 | 2.000 |
| | PLATEC0.6 | Mean | | 1438.7500 | 160.77747 |
| | | 95% Confidence Interval for Mean | Lower Bound | 927.0843 | |
| | | | Upper Bound | 1950.4157 | |
| | | 5% Trimmed Mean | | 1423.1667 | |
| | | Median | | 1298.5000 | |
| | | Variance | | 103397.583 | |
| | | Std. Deviation | | 321.55495 | |
| | | Minimum | | 1239.00 | |
| | | Maximum | | 1919.00 | |
| | | Range | | 680.00 | |
| | | Interquartile Range | | 516.25 | |
| | | Skewness | | 1.948 | 1.014 |
| | | Kurtosis | | 3.835 | 2.619 |
| | PLATEC0.8 | Mean | | 687.2000 | 100.91650 |
| | | 95% Confidence Interval for Mean | Lower Bound | 407.0109 | |
| | | | Upper Bound | 967.3891 | |
| | | 5% Trimmed Mean | | 678.3333 | |
| | | Median | | 632.0000 | |
| | | Variance | | 50920.700 | |
| | | Std. Deviation | | 225.65615 | |
| | | Minimum | | 482.00 | |
| | | Maximum | | 1052.00 | |
| | | Range | | 570.00 | |
| | | Interquartile Range | | 385.00 | |
| | | Skewness | | 1.317 | .913 |
| | | Kurtosis | | 1.658 | 2.000 |
| | PLATEC1.0 | Mean | | 375.8000 | 57.79048 |
| | | 95% Confidence Interval for Mean | Lower Bound | 215.3479 | |
| | | | Upper Bound | 536.2521 | |
| | | 5% Trimmed Mean | | 371.1667 | |
| | | Median | | 385.0000 | |
| | | Variance | | 16698.700 | |
| | | Std. Deviation | | 129.22345 | |

| | | | | | |
|---|---|---|---|---|---|
| | | Minimum | | 258.00 | |
| | | Maximum | | 577.00 | |
| | | Range | | 319.00 | |
| | | Interquartile Range | | 223.00 | |
| | | Skewness | | 1.003 | .913 |
| | | Kurtosis | | .861 | 2.000 |
| | PLATEC1.2 | Mean | | 239.0000 | 30.61590 |
| | | 95% Confidence Interval for Mean | Lower Bound | 107.2704 | |
| | | | Upper Bound | 370.7296 | |
| | | 5% Trimmed Mean | | . | |
| | | Median | | 241.0000 | |
| | | Variance | | 2812.000 | |
| | | Std. Deviation | | 53.02829 | |
| | | Minimum | | 185.00 | |
| | | Maximum | | 291.00 | |
| | | Range | | 106.00 | |
| | | Interquartile Range | | . | |
| | | Skewness | | -.169 | 1.225 |
| | | Kurtosis | | . | . |
| σ | PLA | Mean | | 90.8920 | 11.09518 |
| | | 95% Confidence Interval for Mean | Lower Bound | 60.0868 | |
| | | | Upper Bound | 121.6972 | |
| | | 5% Trimmed Mean | | 90.6172 | |
| | | Median | | 84.3800 | |
| | | Variance | | 615.515 | |
| | | Std. Deviation | | 24.80958 | |
| | | Minimum | | 63.67 | |
| | | Maximum | | 123.06 | |
| | | Range | | 59.39 | |
| | | Interquartile Range | | 47.62 | |
| | | Skewness | | .392 | .913 |
| | | Kurtosis | | -1.960 | 2.000 |
| | PLATEC0.2 | Mean | | 51.1480 | 6.11492 |
| | | 95% Confidence Interval for Mean | Lower Bound | 34.1703 | |
| | | | Upper Bound | 68.1257 | |
| | | 5% Trimmed Mean | | 50.9461 | |
| | | Median | | 51.1600 | |
| | | Variance | | 186.961 | |
| | | Std. Deviation | | 13.67337 | |
| | | Minimum | | 37.63 | |
| | | Maximum | | 68.30 | |
| | | Range | | 30.67 | |
| | | Interquartile Range | | 26.83 | |
| | | Skewness | | .182 | .913 |
| | | Kurtosis | | -2.273 | 2.000 |
| | PLATEC0.4 | Mean | | 50.5480 | 10.32931 |
| | | 95% Confidence Interval for Mean | Lower Bound | 21.8692 | |
| | | | Upper Bound | 79.2268 | |
| | | 5% Trimmed Mean | | 50.3433 | |
| | | Median | | 45.2800 | |
| | | Variance | | 533.473 | |
| | | Std. Deviation | | 23.09704 | |
| | | Minimum | | 23.14 | |
| | | Maximum | | 81.64 | |
| | | Range | | 58.50 | |
| | | Interquartile Range | | 43.16 | |
| | | Skewness | | .346 | .913 |
| | | Kurtosis | | -1.078 | 2.000 |
| | PLATEC0.6 | Mean | | 29.4250 | 2.83200 |
| | | 95% Confidence Interval for Mean | Lower Bound | 20.4123 | |
| | | | Upper Bound | 38.4377 | |
| | | 5% Trimmed Mean | | 29.4800 | |
| | | Median | | 29.9200 | |
| | | Variance | | 32.081 | |
| | | Std. Deviation | | 5.66401 | |
| | | Minimum | | 22.60 | |
| | | Maximum | | 35.26 | |
| | | Range | | 12.66 | |
| | | Interquartile Range | | 10.87 | |
| | | Skewness | | -.354 | 1.014 |
| | | Kurtosis | | -2.275 | 2.619 |
| | PLATEC0.8 | Mean | | 17.5180 | 1.39541 |
| | | 95% Confidence Interval for Mean | Lower Bound | 13.6437 | |
| | | | Upper Bound | 21.3923 | |
| | | 5% Trimmed Mean | | 17.5150 | |
| | | Median | | 17.4800 | |
| | | Variance | | 9.736 | |
| | | Std. Deviation | | 3.12023 | |

| | | | | | |
|---|---|---|---|---|---|
| | | Minimum | | 14.30 | |
| | | Maximum | | 20.79 | |
| | | Range | | 6.49 | |
| | | Interquartile Range | | 6.23 | |
| | | Skewness | | .018 | .913 |
| | | Kurtosis | | -2.966 | 2.000 |
| | PLATEC1.0 | Mean | | 18.9300 | 2.02295 |
| | | 95% Confidence Interval for Mean | Lower Bound | 13.3134 | |
| | | | Upper Bound | 24.5466 | |
| | | 5% Trimmed Mean | | 18.8289 | |
| | | Median | | 16.9100 | |
| | | Variance | | 20.462 | |
| | | Std. Deviation | | 4.52345 | |
| | | Minimum | | 14.89 | |
| | | Maximum | | 24.79 | |
| | | Range | | 9.90 | |
| | | Interquartile Range | | 8.63 | |
| | | Skewness | | .609 | .913 |
| | | Kurtosis | | -2.534 | 2.000 |
| | PLATEC1.2 | Mean | | 20.3967 | 2.11574 |
| | | 95% Confidence Interval for Mean | Lower Bound | 11.2934 | |
| | | | Upper Bound | 29.4999 | |
| | | 5% Trimmed Mean | | . | |
| | | Median | | 21.7400 | |
| | | Variance | | 13.429 | |
| | | Std. Deviation | | 3.66456 | |
| | | Minimum | | 16.25 | |
| | | Maximum | | 23.20 | |
| | | Range | | 6.95 | |
| | | Interquartile Range | | . | |
| | | Skewness | | -1.428 | 1.225 |
| | | Kurtosis | | . | . |
| ε | PLA | Mean | | 5,0460 | ,44270 |
| | | 95% Confidence Interval for Mean | Lower Bound | 3,8169 | |
| | | | Upper Bound | 6,2751 | |
| | | 5% Trimmed Mean | | 5,0178 | |
| | | Median | | 5,1800 | |
| | | Variance | | ,980 | |
| | | Std. Deviation | | ,98991 | |
| | | Minimum | | 4,09 | |
| | | Maximum | | 6,51 | |
| | | Range | | 2,42 | |
| | | Interquartile Range | | 1,78 | |
| | | Skewness | | ,681 | ,913 |
| | | Kurtosis | | -,126 | 2,000 |
| | PLATEC0.2 | Mean | | 3,4060 | ,18052 |
| | | 95% Confidence Interval for Mean | Lower Bound | 2,9048 | |
| | | | Upper Bound | 3,9072 | |
| | | 5% Trimmed Mean | | 3,3994 | |
| | | Median | | 3,4200 | |
| | | Variance | | ,163 | |
| | | Std. Deviation | | ,40365 | |
| | | Minimum | | 2,97 | |
| | | Maximum | | 3,96 | |
| | | Range | | ,99 | |
| | | Interquartile Range | | ,77 | |
| | | Skewness | | ,366 | ,913 |
| | | Kurtosis | | -1,181 | 2,000 |
| | PLATEC0.4 | Mean | | 4,0480 | ,96791 |
| | | 95% Confidence Interval for Mean | Lower Bound | 1,3606 | |
| | | | Upper Bound | 6,7354 | |
| | | 5% Trimmed Mean | | 3,9178 | |
| | | Median | | 3,1800 | |
| | | Variance | | 4,684 | |
| | | Std. Deviation | | 2,16432 | |
| | | Minimum | | 2,62 | |
| | | Maximum | | 7,82 | |
| | | Range | | 5,20 | |
| | | Interquartile Range | | 3,16 | |
| | | Skewness | | 1,969 | ,913 |
| | | Kurtosis | | 3,963 | 2,000 |
| | PLATEC0.6 | Mean | | 52,6350 | 24,46090 |
| | | 95% Confidence Interval for Mean | Lower Bound | -25,2105 | |
| | | | Upper Bound | 130,4805 | |

| | | | | | |
|---|---|---|---|---|---|
| | | 5% Trimmed Mean | | 50,6000 | |
| | | Median | | 34,3200 | |
| | | Variance | | 2393,342 | |
| | | Std. Deviation | | 48,92179 | |
| | | Minimum | | 17,07 | |
| | | Maximum | | 124,83 | |
| | | Range | | 107,76 | |
| | | Interquartile Range | | 82,83 | |
| | | Skewness | | 1,806 | 1,014 |
| | | Kurtosis | | 3,409 | 2,619 |
| | PLATEC0.8 | Mean | | 58,6800 | 3,05653 |
| | | 95% Confidence Interval for Mean | Lower Bound | 50,1937 | |
| | | | Upper Bound | 67,1663 | |
| | | 5% Trimmed Mean | | 58,7000 | |
| | | Median | | 58,8000 | |
| | | Variance | | 46,712 | |
| | | Std. Deviation | | 6,83462 | |
| | | Minimum | | 48,90 | |
| | | Maximum | | 68,10 | |
| | | Range | | 19,20 | |
| | | Interquartile Range | | 10,70 | |
| | | Skewness | | -,129 | ,913 |
| | | Kurtosis | | 1,754 | 2,000 |
| | PLATEC1.0 | Mean | | 68,2520 | 23,79465 |
| | | 95% Confidence Interval for Mean | Lower Bound | 2,1875 | |
| | | | Upper Bound | 134,3165 | |
| | | 5% Trimmed Mean | | 66,5311 | |
| | | Median | | 48,0200 | |
| | | Variance | | 2830,927 | |
| | | Std. Deviation | | 53,20646 | |
| | | Minimum | | 15,64 | |
| | | Maximum | | 151,84 | |
| | | Range | | 136,20 | |
| | | Interquartile Range | | 91,57 | |
| | | Skewness | | 1,140 | ,913 |
| | | Kurtosis | | ,960 | 2,000 |
| | PLATEC1.2 | Mean | | 122,2767 | 12,67994 |
| | | 95% Confidence Interval for Mean | Lower Bound | 67,7193 | |
| | | | Upper Bound | 176,8340 | |
| | | 5% Trimmed Mean | | . | |
| | | Median | | 116,0500 | |
| | | Variance | | 482,343 | |
| | | Std. Deviation | | 21,96230 | |
| | | Minimum | | 104,10 | |
| | | Maximum | | 146,68 | |
| | | Range | | 42,58 | |
| | | Interquartile Range | | . | |
| | | Skewness | | 1,173 | 1,225 |
| | | Kurtosis | | . | . |

**Table S2:** Overall mean values significant difference hypothesis test using Median Test results for E,$\sigma_{uts}$, ε%

**Hypothesis Test Summary**

| | Null Hypothesis | Test | Sig.[a,b] | Decision |
|---|---|---|---|---|
| 1 | The medians of E are the same across categories of Film_group2. | Independent-Samples Median Test | <.001 | Reject the null hypothesis. |
| 2 | The medians of σ are the same across categories of Film_group2. | Independent-Samples Median Test | <.001 | Reject the null hypothesis. |
| 3 | The medians of ε are the same across categories of Film_group2. | Independent-Samples Median Test | <.001 | Reject the null hypothesis. |

a. The significance level is .050.

b. Asymptotic significance is displayed.

**Table S3:** Paiwise mean values significant difference hypothesis test using Median Test results for Young Modulus E

**Pairwise Comparisons of Film_group2**

| Sample 1-Sample 2 | Test Statistic | Sig. | Adj. Sig.[a] |
|---|---|---|---|

| | | | |
|---|---|---|---|
| PLATEC1.2-PLATEC1.0 | .533 | .465 | 1.000 |
| PLATEC1.2-PLATEC0.8 | 4.800 | .028 | .598 |
| PLATEC1.2-PLATEC0.6 | 3.938 | .047 | .992 |
| PLATEC1.2-PLATEC0.2 | 4.800 | .028 | .598 |
| PLATEC1.2-PLATEC0.4 | 4.800 | .028 | .598 |
| PLATEC1.2-PLA | 4.800 | .028 | .598 |
| PLATEC1.0-PLATEC0.8 | 3.600 | .058 | 1.000 |
| PLATEC1.0-PLATEC0.6 | 9.000 | .003 | .057 |
| PLATEC1.0-PLATEC0.2 | 10.000 | .002 | .033 |
| PLATEC1.0-PLATEC0.4 | 10.000 | .002 | .033 |
| PLATEC1.0-PLA | 10.000 | .002 | .033 |
| PLATEC0.8-PLATEC0.6 | 9.000 | .003 | .057 |
| PLATEC0.8-PLATEC0.2 | 10.000 | .002 | .033 |
| PLATEC0.8-PLATEC0.4 | 10.000 | .002 | .033 |
| PLATEC0.8-PLA | 10.000 | .002 | .033 |
| PLATEC0.6-PLATEC0.2 | 5.760 | .016 | .344 |
| PLATEC0.6-PLATEC0.4 | 5.760 | .016 | .344 |
| PLATEC0.6-PLA | 5.760 | .016 | .344 |
| PLATEC0.2-PLATEC0.4 | .400 | .527 | 1.000 |
| PLATEC0.2-PLA | .400 | .527 | 1.000 |
| PLATEC0.4-PLA | .400 | .527 | 1.000 |

Each row tests the null hypothesis that the Sample 1 and Sample 2 distributions are the same.
Asymptotic significances (2-sided tests) are displayed. The significance level is .050.
a. Significance values have been adjusted by the Bonferroni correction for multiple tests.

**Table S4:** Pairwise mean values significant difference hypothesis test using Median Test results for Ultimate Strength $\sigma_{uts}$.

Pairwise Comparisons of Film_group2

| Sample 1-Sample 2 | Test Statistic | Sig. | Adj. Sig.[a] |
|---|---|---|---|
| PLATEC1.0-PLATEC0.8 | .400 | .527 | 1.000 |
| PLATEC1.0-PLATEC1.2 | .533 | .465 | 1.000 |
| PLATEC1.0-PLATEC0.6 | 2.723 | .099 | 1.000 |
| PLATEC1.0-PLATEC0.4 | 3.600 | .058 | 1.000 |
| PLATEC1.0-PLATEC0.2 | 10.000 | .002 | .033 |
| PLATEC1.0-PLA | 10.000 | .002 | .033 |
| PLATEC0.8-PLATEC1.2 | .533 | .465 | 1.000 |
| PLATEC0.8-PLATEC0.6 | 9.000 | .003 | .057 |
| PLATEC0.8-PLATEC0.4 | 10.000 | .002 | .033 |
| PLATEC0.8-PLATEC0.2 | 10.000 | .002 | .033 |
| PLATEC0.8-PLA | 10.000 | .002 | .033 |
| PLATEC1.2-PLATEC0.6 | 3.938 | .047 | .992 |
| PLATEC1.2-PLATEC0.4 | 4.800 | .028 | .598 |
| PLATEC1.2-PLATEC0.2 | 4.800 | .028 | .598 |
| PLATEC1.2-PLA | 4.800 | .028 | .598 |
| PLATEC0.6-PLATEC0.4 | 5.760 | .016 | .344 |
| PLATEC0.6-PLATEC0.2 | 5.760 | .016 | .344 |
| PLATEC0.6-PLA | 5.760 | .016 | .344 |
| PLATEC0.4-PLATEC0.2 | .400 | .527 | 1.000 |
| PLATEC0.4-PLA | 3.600 | .058 | 1.000 |
| PLATEC0.2-PLA | 3.600 | .058 | 1.000 |

Each row tests the null hypothesis that the Sample 1 and Sample 2 distributions are the same.
Asymptotic significances (2-sided tests) are displayed. The significance level is .050.
a. Significance values have been adjusted by the Bonferroni correction for multiple tests.

**Table S5:** Pairwise mean values significant difference hypothesis test using Median Test results for elongation at break ε%.

**Pairwise Comparisons of Film_group2**

| Sample 1-Sample 2 | Test Statistic | Sig. | Adj. Sig.[a] |
|---|---|---|---|
| PLATEC0.4-PLATEC0.2 | ,400 | ,527 | 1,000 |
| PLATEC0.4-PLA | 3,600 | ,058 | 1,000 |
| PLATEC0.4-PLATEC0.6 | 9,000 | ,003 | ,057 |
| PLATEC0.4-PLATEC1.0 | 10,000 | ,002 | ,033 |
| PLATEC0.4-PLATEC0.8 | 10,000 | ,002 | ,033 |
| PLATEC0.4-PLATEC1.2 | 4,800 | ,028 | ,598 |
| PLATEC0.2-PLA | 10,000 | ,002 | ,033 |
| PLATEC0.2-PLATEC0.6 | 9,000 | ,003 | ,057 |
| PLATEC0.2-PLATEC1.0 | 10,000 | ,002 | ,033 |
| PLATEC0.2-PLATEC0.8 | 10,000 | ,002 | ,033 |
| PLATEC0.2-PLATEC1.2 | 4,800 | ,028 | ,598 |
| PLA-PLATEC0.6 | 9,000 | ,003 | ,057 |
| PLA-PLATEC1.0 | 10,000 | ,002 | ,033 |

| | | | |
|---|---|---|---|
| PLA-PLATEC0.8 | 10,000 | ,002 | ,033 |
| PLA-PLATEC1.2 | 4,800 | ,028 | ,598 |
| PLATEC0.6-PLATEC1.0 | 1,103 | ,294 | 1,000 |
| PLATEC0.6-PLATEC0.8 | 1,103 | ,294 | 1,000 |
| PLATEC0.6-PLATEC1.2 | 1,215 | ,270 | 1,000 |
| PLATEC1.0-PLATEC0.8 | ,400 | ,527 | 1,000 |
| PLATEC1.0-PLATEC1.2 | 4,800 | ,028 | ,598 |
| PLATEC0.8-PLATEC1.2 | 4,800 | ,028 | ,598 |

Each row tests the null hypothesis that the Sample 1 and Sample 2 distributions are the same.
Asymptotic significances (2-sided tests) are displayed. The significance level is ,050.
a. Significance values have been adjusted by the Bonferroni correction for multiple tests.

**Table S6:** Descriptive statistics for Oxygen Permeability Coefficient ($P_{O2}$), Water/Vapor Diffusion Coefficient ($D_{wv}$), $EC_{50}$, %weight loss

**Descriptives**[a.b.c.d]

| | Film_group1 | | | Statistic | Std. Error |
|---|---|---|---|---|---|
| PeO2 | PLA | Mean | | 2.6381467e-009 | 1.63031919e-010 |
| | | 95% Confidence Interval for Mean | Lower Bound | 1.9366769e-009 | |
| | | | Upper Bound | 3.3396164e-009 | |
| | | 5% Trimmed Mean | | . | |
| | | Median | | 2.6625900e-009 | |
| | | Variance | | .000 | |
| | | Std. Deviation | | 2.82379567e-010 | |
| | | Minimum | | 2.34434e-009 | |
| | | Maximum | | 2.90751e-009 | |
| | | Range | | 5.63170e-010 | |
| | | Interquartile Range | | . | |
| | | Skewness | | -.387 | 1.225 |
| | | Kurtosis | | . | . |
| | PLATEC0.2 | Mean | | 2.6586533e-009 | 6.61005436e-010 |
| | | 95% Confidence Interval for Mean | Lower Bound | -1.8542351e-010 | |
| | | | Upper Bound | 5.5027302e-009 | |
| | | 5% Trimmed Mean | | . | |
| | | Median | | 2.6586500e-009 | |
| | | Variance | | .000 | |
| | | Std. Deviation | | 1.14489500e-009 | |
| | | Minimum | | 1.51376e-009 | |
| | | Maximum | | 3.80355e-009 | |
| | | Range | | 2.28979e-009 | |
| | | Interquartile Range | | . | |
| | | Skewness | | .000 | 1.225 |
| | | Kurtosis | | . | . |
| | PLATEC0.4 | Mean | | 2.3816233e-009 | 3.74200917e-010 |
| | | 95% Confidence Interval for Mean | Lower Bound | 7.7156674e-010 | |
| | | | Upper Bound | 3.9916799e-009 | |
| | | 5% Trimmed Mean | | . | |
| | | Median | | 2.3816200e-009 | |
| | | Variance | | .000 | |
| | | Std. Deviation | | 6.48135000e-010 | |
| | | Minimum | | 1.73349e-009 | |
| | | Maximum | | 3.02976e-009 | |
| | | Range | | 1.29627e-009 | |
| | | Interquartile Range | | . | |
| | | Skewness | | .000 | 1.225 |
| | | Kurtosis | | . | . |
| | PLATEC0.6 | Mean | | 2.2438933e-009 | 6.39880190e-010 |
| | | 95% Confidence Interval for Mean | Lower Bound | -5.0928891e-010 | |
| | | | Upper Bound | 4.9970756e-009 | |
| | | 5% Trimmed Mean | | . | |
| | | Median | | 2.2438900e-009 | |
| | | Variance | | .000 | |
| | | Std. Deviation | | 1.10830500e-009 | |
| | | Minimum | | 1.13559e-009 | |
| | | Maximum | | 3.35220e-009 | |
| | | Range | | 2.21661e-009 | |
| | | Interquartile Range | | . | |
| | | Skewness | | .000 | 1.225 |
| | | Kurtosis | | . | . |
| | PLATEC0.8 | Mean | | 3.2882400e-009 | 3.51271451e-010 |
| | | 95% Confidence Interval for Mean | Lower Bound | 1.7768409e-009 | |
| | | | Upper Bound | 4.7996391e-009 | |

| | | | | | |
|---|---|---|---|---|---|
| | | 5% Trimmed Mean | | . | |
| | | Median | | 3.2882400e-009 | |
| | | Variance | | .000 | |
| | | Std. Deviation | | 6.08420000e-010 | |
| | | Minimum | | 2.67982e-009 | |
| | | Maximum | | 3.89666e-009 | |
| | | Range | | 1.21684e-009 | |
| | | Interquartile Range | | . | |
| | | Skewness | | .000 | 1.225 |
| | | Kurtosis | | . | . |
| | PLATEC1.0 | Mean | | 3.2711200e-009 | 8.74108308e-012 |
| | | 95% Confidence Interval for Mean | Lower Bound | 3.2335102e-009 | |
| | | | Upper Bound | 3.3087298e-009 | |
| | | 5% Trimmed Mean | | . | |
| | | Median | | 3.2711200e-009 | |
| | | Variance | | .000 | |
| | | Std. Deviation | | 1.51400000e-011 | |
| | | Minimum | | 3.25598e-009 | |
| | | Maximum | | 3.28626e-009 | |
| | | Range | | 3.02800e-011 | |
| | | Interquartile Range | | . | |
| | | Skewness | | .000 | 1.225 |
| | | Kurtosis | | . | . |
| | PLATEC1.2 | Mean | | 4.6219733e-009 | 1.53201418e-011 |
| | | 95% Confidence Interval for Mean | Lower Bound | 4.5560561e-009 | |
| | | | Upper Bound | 4.6878906e-009 | |
| | | 5% Trimmed Mean | | . | |
| | | Median | | 4.6221100e-009 | |
| | | Variance | | .000 | |
| | | Std. Deviation | | 2.65352640e-011 | |
| | | Minimum | | 4.59537e-009 | |
| | | Maximum | | 4.64844e-009 | |
| | | Range | | 5.30700e-011 | |
| | | Interquartile Range | | . | |
| | | Skewness | | -.023 | 1.225 |
| | | Kurtosis | | . | . |
| Dwv | PLA | Mean | | 1.4030000e-004 | 4.90747729e-006 |
| | | 95% Confidence Interval for Mean | Lower Bound | 1.1918483e-004 | |
| | | | Upper Bound | 1.6141517e-004 | |
| | | 5% Trimmed Mean | | . | |
| | | Median | | 1.4030000e-004 | |
| | | Variance | | .000 | |
| | | Std. Deviation | | 8.50000000e-006 | |
| | | Minimum | | 1.31800e-004 | |
| | | Maximum | | 1.48800e-004 | |
| | | Range | | 1.70000e-005 | |
| | | Interquartile Range | | . | |
| | | Skewness | | .000 | 1.225 |
| | | Kurtosis | | . | . |
| | PLATEC0.2 | Mean | | 9.0900000e-005 | 1.19511506e-005 |
| | | 95% Confidence Interval for Mean | Lower Bound | 3.9478349e-005 | |
| | | | Upper Bound | 1.4232165e-004 | |
| | | 5% Trimmed Mean | | . | |
| | | Median | | 9.0900000e-005 | |
| | | Variance | | .000 | |
| | | Std. Deviation | | 2.07000000e-005 | |
| | | Minimum | | 7.02000e-005 | |
| | | Maximum | | 1.11600e-004 | |
| | | Range | | 4.14000e-005 | |
| | | Interquartile Range | | . | |
| | | Skewness | | .000 | 1.225 |
| | | Kurtosis | | . | . |
| | PLATEC0.4 | Mean | | 7.5800000e-005 | 4.96521232e-006 |
| | | 95% Confidence Interval for Mean | Lower Bound | 5.4436416e-005 | |
| | | | Upper Bound | 9.7163584e-005 | |
| | | 5% Trimmed Mean | | . | |
| | | Median | | 7.5800000e-005 | |
| | | Variance | | .000 | |
| | | Std. Deviation | | 8.60000000e-006 | |
| | | Minimum | | 6.72000e-005 | |
| | | Maximum | | 8.44000e-005 | |

| | | | | | |
|---|---|---|---|---|---|
| | | Range | | 1.72000e-005 | |
| | | Interquartile Range | | . | |
| | | Skewness | | .000 | 1.225 |
| | | Kurtosis | | . | . |
| | PLATEC0.6 | Mean | | 1.1500000e-004 | 1.99185843e-005 |
| | | 95% Confidence Interval for Mean | Lower Bound | 2.9297249e-005 | |
| | | | Upper Bound | 2.0070275e-004 | |
| | | 5% Trimmed Mean | | . | |
| | | Median | | 1.1500000e-004 | |
| | | Variance | | .000 | |
| | | Std. Deviation | | 3.45000000e-005 | |
| | | Minimum | | 8.05000e-005 | |
| | | Maximum | | 1.49500e-004 | |
| | | Range | | 6.90000e-005 | |
| | | Interquartile Range | | . | |
| | | Skewness | | .000 | 1.225 |
| | | Kurtosis | | . | . |
| | PLATEC0.8 | Mean | | 7.6400000e-005 | 1.18356805e-005 |
| | | 95% Confidence Interval for Mean | Lower Bound | 2.5475177e-005 | |
| | | | Upper Bound | 1.2732482e-004 | |
| | | 5% Trimmed Mean | | . | |
| | | Median | | 7.6400000e-005 | |
| | | Variance | | .000 | |
| | | Std. Deviation | | 2.05000000e-005 | |
| | | Minimum | | 5.59000e-005 | |
| | | Maximum | | 9.69000e-005 | |
| | | Range | | 4.10000e-005 | |
| | | Interquartile Range | | . | |
| | | Skewness | | .000 | 1.225 |
| | | Kurtosis | | . | . |
| | PLATEC1.0 | Mean | | 1.0400000e-004 | 1.63967476e-005 |
| | | 95% Confidence Interval for Mean | Lower Bound | 3.3450489e-005 | |
| | | | Upper Bound | 1.7454951e-004 | |
| | | 5% Trimmed Mean | | . | |
| | | Median | | 1.0400000e-004 | |
| | | Variance | | .000 | |
| | | Std. Deviation | | 2.84000000e-005 | |
| | | Minimum | | 7.56000e-005 | |
| | | Maximum | | 1.32400e-004 | |
| | | Range | | 5.68000e-005 | |
| | | Interquartile Range | | . | |
| | | Skewness | | .000 | 1.225 |
| | | Kurtosis | | . | . |
| | PLATEC1.2 | Mean | | 1.0400000e-004 | 1.99185843e-005 |
| | | 95% Confidence Interval for Mean | Lower Bound | 1.8297249e-005 | |
| | | | Upper Bound | 1.8970275e-004 | |
| | | 5% Trimmed Mean | | . | |
| | | Median | | 1.0400000e-004 | |
| | | Variance | | .000 | |
| | | Std. Deviation | | 3.45000000e-005 | |
| | | Minimum | | 6.95000e-005 | |
| | | Maximum | | 1.38500e-004 | |
| | | Range | | 6.90000e-005 | |
| | | Interquartile Range | | . | |
| | | Skewness | | .000 | 1.225 |
| | | Kurtosis | | . | . |
| EC50 | PLA | Mean | | 571.3567 | 37.81034 |
| | | 95% Confidence Interval for Mean | Lower Bound | 408.6719 | |
| | | | Upper Bound | 734.0414 | |
| | | 5% Trimmed Mean | | . | |
| | | Median | | 584.2200 | |
| | | Variance | | 4288.865 | |
| | | Std. Deviation | | 65.48943 | |
| | | Minimum | | 500.39 | |
| | | Maximum | | 629.46 | |
| | | Range | | 129.07 | |
| | | Interquartile Range | | . | |
| | | Skewness | | -.850 | 1.225 |
| | | Kurtosis | | . | . |
| | PLATEC0.2 | Mean | | 531.9833 | 61.74068 |
| | | 95% Confidence Interval for Mean | Lower Bound | 266.3346 | |

| | | | | |
|---|---|---|---|---|
| | | Upper Bound | 797.6321 | |
| | 5% Trimmed Mean | | . | |
| | Median | | 538.1400 | |
| | Variance | | 11435.736 | |
| | Std. Deviation | | 106.93800 | |
| | Minimum | | 422.10 | |
| | Maximum | | 635.71 | |
| | Range | | 213.61 | |
| | Interquartile Range | | . | |
| | Skewness | | -.258 | 1.225 |
| | Kurtosis | | . | . |
| PLATEC0.4 | Mean | | 330.8167 | 26.95398 |
| | 95% Confidence Interval for Mean | Lower Bound | 214.8431 | |
| | | Upper Bound | 446.7903 | |
| | 5% Trimmed Mean | | . | |
| | Median | | 314.6800 | |
| | Variance | | 2179.551 | |
| | Std. Deviation | | 46.68566 | |
| | Minimum | | 294.34 | |
| | Maximum | | 383.43 | |
| | Range | | 89.09 | |
| | Interquartile Range | | . | |
| | Skewness | | 1.370 | 1.225 |
| | Kurtosis | | . | . |
| PLATEC0.6 | Mean | | 212.5733 | 7.00485 |
| | 95% Confidence Interval for Mean | Lower Bound | 182.4339 | |
| | | Upper Bound | 242.7128 | |
| | 5% Trimmed Mean | | . | |
| | Median | | 208.0400 | |
| | Variance | | 147.204 | |
| | Std. Deviation | | 12.13275 | |
| | Minimum | | 203.36 | |
| | Maximum | | 226.32 | |
| | Range | | 22.96 | |
| | Interquartile Range | | . | |
| | Skewness | | 1.447 | 1.225 |
| | Kurtosis | | . | . |
| PLATEC0.8 | Mean | | 220.4867 | 29.58098 |
| | 95% Confidence Interval for Mean | Lower Bound | 93.2100 | |
| | | Upper Bound | 347.7634 | |
| | 5% Trimmed Mean | | . | |
| | Median | | 229.0200 | |
| | Variance | | 2625.103 | |
| | Std. Deviation | | 51.23576 | |
| | Minimum | | 165.52 | |
| | Maximum | | 266.92 | |
| | Range | | 101.40 | |
| | Interquartile Range | | . | |
| | Skewness | | -.729 | 1.225 |
| | Kurtosis | | . | . |
| PLATEC1.0 | Mean | | 177.9567 | 5.96601 |
| | 95% Confidence Interval for Mean | Lower Bound | 152.2870 | |
| | | Upper Bound | 203.6263 | |
| | 5% Trimmed Mean | | . | |
| | Median | | 182.8100 | |
| | Variance | | 106.780 | |
| | Std. Deviation | | 10.33343 | |
| | Minimum | | 166.09 | |
| | Maximum | | 184.97 | |
| | Range | | 18.88 | |
| | Interquartile Range | | . | |
| | Skewness | | -1.647 | 1.225 |
| | Kurtosis | | . | . |
| PLATEC1.2 | Mean | | 101.7933 | 2.62038 |
| | 95% Confidence Interval for Mean | Lower Bound | 90.5187 | |
| | | Upper Bound | 113.0679 | |
| | 5% Trimmed Mean | | . | |
| | Median | | 102.8800 | |
| | Variance | | 20.599 | |
| | Std. Deviation | | 4.53864 | |
| | Minimum | | 96.81 | |

| | | | | | |
|---|---|---|---|---|---|
| | | Maximum | | 105.69 | |
| | | Range | | 8.88 | |
| | | Interquartile Range | | . | |
| | | Skewness | | -1.016 | 1.225 |
| | | Kurtosis | | . | . |
| W.L | PLA | Mean | | .0600 | .03055 |
| | | 95% Confidence Interval for Mean | Lower Bound | -.0714 | |
| | | | Upper Bound | .1914 | |
| | | 5% Trimmed Mean | | . | |
| | | Median | | .0800 | |
| | | Variance | | .003 | |
| | | Std. Deviation | | .05292 | |
| | | Minimum | | .00 | |
| | | Maximum | | .10 | |
| | | Range | | .10 | |
| | | Interquartile Range | | . | |
| | | Skewness | | -1.458 | 1.225 |
| | | Kurtosis | | . | . |
| | PLATEC0.2 | Mean | | .1033 | .05548 |
| | | 95% Confidence Interval for Mean | Lower Bound | -.1354 | |
| | | | Upper Bound | .3420 | |
| | | 5% Trimmed Mean | | . | |
| | | Median | | .1200 | |
| | | Variance | | .009 | |
| | | Std. Deviation | | .09609 | |
| | | Minimum | | .00 | |
| | | Maximum | | .19 | |
| | | Range | | .19 | |
| | | Interquartile Range | | . | |
| | | Skewness | | -.757 | 1.225 |
| | | Kurtosis | | . | . |
| | PLATEC0.4 | Mean | | .6567 | .10868 |
| | | 95% Confidence Interval for Mean | Lower Bound | .1891 | |
| | | | Upper Bound | 1.1243 | |
| | | 5% Trimmed Mean | | . | |
| | | Median | | .7500 | |
| | | Variance | | .035 | |
| | | Std. Deviation | | .18824 | |
| | | Minimum | | .44 | |
| | | Maximum | | .78 | |
| | | Range | | .34 | |
| | | Interquartile Range | | . | |
| | | Skewness | | -1.683 | 1.225 |
| | | Kurtosis | | . | . |
| | PLATEC0.6 | Mean | | 1.9900 | .38188 |
| | | 95% Confidence Interval for Mean | Lower Bound | .3469 | |
| | | | Upper Bound | 3.6331 | |
| | | 5% Trimmed Mean | | . | |
| | | Median | | 1.7400 | |
| | | Variance | | .438 | |
| | | Std. Deviation | | .66144 | |
| | | Minimum | | 1.49 | |
| | | Maximum | | 2.74 | |
| | | Range | | 1.25 | |
| | | Interquartile Range | | . | |
| | | Skewness | | 1.458 | 1.225 |
| | | Kurtosis | | . | . |
| | PLATEC0.8 | Mean | | 2.6000 | .49501 |
| | | 95% Confidence Interval for Mean | Lower Bound | .4702 | |
| | | | Upper Bound | 4.7298 | |
| | | 5% Trimmed Mean | | . | |
| | | Median | | 2.1100 | |
| | | Variance | | .735 | |
| | | Std. Deviation | | .85738 | |
| | | Minimum | | 2.10 | |
| | | Maximum | | 3.59 | |
| | | Range | | 1.49 | |
| | | Interquartile Range | | . | |
| | | Skewness | | 1.732 | 1.225 |
| | | Kurtosis | | . | . |
| | PLATEC1.0 | Mean | | 1.9967 | .27187 |

| | | | | | |
|---|---|---|---|---|---|
| | | 95% Confidence Interval for Mean | Lower Bound | .8269 | |
| | | | Upper Bound | 3.1664 | |
| | | 5% Trimmed Mean | | . | |
| | | Median | | 2.0300 | |
| | | Variance | | .222 | |
| | | Std. Deviation | | .47089 | |
| | | Minimum | | 1.51 | |
| | | Maximum | | 2.45 | |
| | | Range | | .94 | |
| | | Interquartile Range | | . | |
| | | Skewness | | -.317 | 1.225 |
| | | Kurtosis | | . | . |
| | PLATEC1.2 | Mean | | 2.2933 | .32297 |
| | | 95% Confidence Interval for Mean | Lower Bound | .9037 | |
| | | | Upper Bound | 3.6830 | |
| | | 5% Trimmed Mean | | . | |
| | | Median | | 2.5000 | |
| | | Variance | | .313 | |
| | | Std. Deviation | | .55940 | |
| | | Minimum | | 1.66 | |
| | | Maximum | | 2.72 | |
| | | Range | | 1.06 | |
| | | Interquartile Range | | . | |
| | | Skewness | | -1.436 | 1.225 |
| | | Kurtosis | | . | . |

a. There are no valid cases for PeO2 when Film_group1 = .. Statistics cannot be computed for this level.
b. There are no valid cases for Dwv when Film_group1 = .. Statistics cannot be computed for this level.
c. There are no valid cases for EC50 when Film_group1 = .. Statistics cannot be computed for this level.
d. There are no valid cases for W.L when Film_group1 = .. Statistics cannot be computed for this level.

**Table S7:** Overall mean values significant difference hypothesis test using Median Test results for E,$\sigma_{uts}$, ε%

Hypothesis Test Summary

| | Null Hypothesis | Test | Sig.a.b | Decision |
|---|---|---|---|---|
| 1 | The medians of PeO2 are the same across categories of Film_group1. | Independent-Samples Median Test | .043 | Reject the null hypothesis. |
| 2 | The medians of Dwv are the same across categories of Film_group1. | Independent-Samples Median Test | .112 | Retain the null hypothesis. |
| 3 | The medians of EC50 are the same across categories of Film_group1. | Independent-Samples Median Test | .005 | Reject the null hypothesis. |
| 4 | The medians of W.L are the same across categories of Film_group1. | Independent-Samples Median Test | .016 | Reject the null hypothesis. |

a. The significance level is .050.
b. Asymptotic significance is displayed.

**Table S8:** Pairwise mean values significant difference hypothesis test using Median Test results for $P_{O2}$.

**Pairwise Comparisons of Film_group1**

| Sample 1-Sample 2 | Test Statistic | Sig. | Adj. Sig.[a] |
|---|---|---|---|
| PLATEC0.6-PLATEC0.4 | .667 | .414 | 1.000 |
| PLATEC0.6-PLATEC0.2 | .667 | .414 | 1.000 |
| PLATEC0.6-PLA | .667 | .414 | 1.000 |
| PLATEC0.6-PLATEC1.0 | .667 | .414 | 1.000 |
| PLATEC0.6-PLATEC0.8 | .667 | .414 | 1.000 |
| PLATEC0.6-PLATEC1.2 | 6.000 | .014 | .300 |
| PLATEC0.4-PLATEC0.2 | .667 | .414 | 1.000 |
| PLATEC0.4-PLA | .667 | .414 | 1.000 |
| PLATEC0.4-PLATEC1.0 | 6.000 | .014 | .300 |
| PLATEC0.4-PLATEC0.8 | .667 | .414 | 1.000 |
| PLATEC0.4-PLATEC1.2 | 6.000 | .014 | .300 |
| PLATEC0.2-PLA | .667 | .414 | 1.000 |
| PLATEC0.2-PLATEC1.0 | .667 | .414 | 1.000 |
| PLATEC0.2-PLATEC0.8 | .667 | .414 | 1.000 |
| PLATEC0.2-PLATEC1.2 | 6.000 | .014 | .300 |
| PLA-PLATEC1.0 | 6.000 | .014 | .300 |
| PLA-PLATEC0.8 | .667 | .414 | 1.000 |
| PLA-PLATEC1.2 | 6.000 | .014 | .300 |
| PLATEC1.0-PLATEC0.8 | .667 | .414 | 1.000 |
| PLATEC1.0-PLATEC1.2 | 6.000 | .014 | .300 |
| PLATEC0.8-PLATEC1.2 | 6.000 | .014 | .300 |

Each row tests the null hypothesis that the Sample 1 and Sample 2 distributions are the same.
Asymptotic significances (2-sided tests) are displayed. The significance level is .050.
a. Significance values have been adjusted by the Bonferroni correction for multiple tests.

**Table S9:** Pairwise mean values significant difference hypothesis test using Median Test results for $D_{wv}$.

Pairwise Comparisons of Film_group1

| Sample 1-Sample 2 | Test Statistic | Sig. | Adj. Sig.[a] |
|---|---|---|---|
| PLATEC0.4-PLATEC0.8 | .667 | .414 | 1.000 |
| PLATEC0.4-PLATEC0.2 | .667 | .414 | 1.000 |
| PLATEC0.4-PLATEC1.0 | .667 | .414 | 1.000 |
| PLATEC0.4-PLATEC1.2 | .667 | .414 | 1.000 |
| PLATEC0.4-PLATEC0.6 | .667 | .414 | 1.000 |
| PLATEC0.4-PLA | 6.000 | .014 | .300 |
| PLATEC0.8-PLATEC0.2 | .667 | .414 | 1.000 |
| PLATEC0.8-PLATEC1.0 | .667 | .414 | 1.000 |
| PLATEC0.8-PLATEC1.2 | .667 | .414 | 1.000 |
| PLATEC0.8-PLATEC0.6 | .667 | .414 | 1.000 |
| PLATEC0.8-PLA | 6.000 | .014 | .300 |
| PLATEC0.2-PLATEC1.0 | .667 | .414 | 1.000 |
| PLATEC0.2-PLATEC1.2 | .667 | .414 | 1.000 |
| PLATEC0.2-PLATEC0.6 | .667 | .414 | 1.000 |
| PLATEC0.2-PLA | 6.000 | .014 | .300 |
| PLATEC1.0-PLA | .667 | .414 | 1.000 |
| PLATEC1.2-PLA | .667 | .414 | 1.000 |
| PLATEC1.0-PLATEC0.6 | .667 | .414 | 1.000 |
| PLATEC1.2-PLATEC0.6 | .667 | .414 | 1.000 |
| PLATEC1.0-PLATEC1.2 | .000 | 1.000 | 1.000 |
| PLATEC0.6-PLA | .667 | .414 | 1.000 |

Each row tests the null hypothesis that the Sample 1 and Sample 2 distributions are the same.
Asymptotic significances (2-sided tests) are displayed. The significance level is .050.
a. Significance values have been adjusted by the Bonferroni correction for multiple tests.

**Table S10:** Pairwise mean values significant difference hypothesis test using Median Test results for $EC_{50}$.

Pairwise Comparisons of Film_group1

| Sample 1-Sample 2 | Test Statistic | Sig. | Adj. Sig.[a] |
|---|---|---|---|
| PLATEC1.2-PLATEC1.0 | 6.000 | .014 | .300 |
| PLATEC1.2-PLATEC0.6 | 6.000 | .014 | .300 |
| PLATEC1.2-PLATEC0.8 | 6.000 | .014 | .300 |
| PLATEC1.2-PLATEC0.4 | 6.000 | .014 | .300 |
| PLATEC1.2-PLATEC0.2 | 6.000 | .014 | .300 |
| PLATEC1.2-PLA | 6.000 | .014 | .300 |
| PLATEC1.0-PLATEC0.6 | 6.000 | .014 | .300 |
| PLATEC1.0-PLATEC0.8 | .667 | .414 | 1.000 |
| PLATEC1.0-PLATEC0.4 | 6.000 | .014 | .300 |
| PLATEC1.0-PLATEC0.2 | 6.000 | .014 | .300 |
| PLATEC1.0-PLA | 6.000 | .014 | .300 |
| PLATEC0.6-PLATEC0.8 | .667 | .414 | 1.000 |
| PLATEC0.6-PLATEC0.4 | 6.000 | .014 | .300 |
| PLATEC0.6-PLATEC0.2 | 6.000 | .014 | .300 |
| PLATEC0.6-PLA | 6.000 | .014 | .300 |
| PLATEC0.8-PLATEC0.4 | 6.000 | .014 | .300 |
| PLATEC0.8-PLATEC0.2 | 6.000 | .014 | .300 |
| PLATEC0.8-PLA | 6.000 | .014 | .300 |
| PLATEC0.4-PLATEC0.2 | 6.000 | .014 | .300 |
| PLATEC0.4-PLA | 6.000 | .014 | .300 |
| PLATEC0.2-PLA | .667 | .414 | 1.000 |

Each row tests the null hypothesis that the Sample 1 and Sample 2 distributions are the same.
Asymptotic significances (2-sided tests) are displayed. The significance level is .050.
a. Significance values have been adjusted by the Bonferroni correction for multiple tests.

**Table S11:** Pairwise mean values significant difference hypothesis test using Median Test results for %weight loss.

Pairwise Comparisons of Film_group1

| Sample 1-Sample 2 | Test Statistic | Sig. | Adj. Sig.[a] |
|---|---|---|---|
| PLA-PLATEC0.2 | .667 | .414 | 1.000 |
| PLA-PLATEC0.4 | 6.000 | .014 | .300 |
| PLA-PLATEC0.6 | 6.000 | .014 | .300 |
| PLA-PLATEC1.0 | 6.000 | .014 | .300 |
| PLA-PLATEC0.8 | 6.000 | .014 | .300 |
| PLA-PLATEC1.2 | 6.000 | .014 | .300 |
| PLATEC0.2-PLATEC0.4 | 6.000 | .014 | .300 |
| PLATEC0.2-PLATEC0.6 | 6.000 | .014 | .300 |
| PLATEC0.2-PLATEC1.0 | 6.000 | .014 | .300 |
| PLATEC0.2-PLATEC0.8 | 6.000 | .014 | .300 |
| PLATEC0.2-PLATEC1.2 | 6.000 | .014 | .300 |

| | | | |
|---|---|---|---|
| PLATEC0.4-PLATEC0.6 | 6.000 | .014 | .300 |
| PLATEC0.4-PLATEC1.0 | 6.000 | .014 | .300 |
| PLATEC0.4-PLATEC0.8 | 6.000 | .014 | .300 |
| PLATEC0.4-PLATEC1.2 | 6.000 | .014 | .300 |
| PLATEC0.6-PLATEC1.0 | .667 | .414 | 1.000 |
| PLATEC0.6-PLATEC0.8 | .667 | .414 | 1.000 |
| PLATEC0.6-PLATEC1.2 | .667 | .414 | 1.000 |
| PLATEC1.0-PLATEC0.8 | .667 | .414 | 1.000 |
| PLATEC1.0-PLATEC1.2 | .667 | .414 | 1.000 |
| PLATEC0.8-PLATEC1.2 | .667 | .414 | 1.000 |

Each row tests the null hypothesis that the Sample 1 and Sample 2 distributions are the same.
Asymptotic significances (2-sided tests) are displayed. The significance level is .050.
a. Significance values have been adjusted by the Bonferroni correction for multiple tests.

**Table S12**: Description statistics of cohesion, color, heme-iron, odor, and TBA from DAY 0 to DAY 6

Descriptives[a,b,c,d,e,f,g,h,i,j,k,l,m,n,o,p,q,r,s,t]

| | Film_group3 | | | Statistic | Std. Error |
|---|---|---|---|---|---|
| cohesionDAY0 | CONTROL | Mean | | 5.0000 | .00000 |
| | | 95% Confidence Interval for Mean | Lower Bound | 5.0000 | |
| | | | Upper Bound | 5.0000 | |
| | | 5% Trimmed Mean | | 5.0000 | |
| | | Median | | 5.0000 | |
| | | Variance | | .000 | |
| | | Std. Deviation | | .00000 | |
| | | Minimum | | 5.00 | |
| | | Maximum | | 5.00 | |
| | | Range | | .00 | |
| | | Interquartile Range | | .00 | |
| | | Skewness | | . | . |
| | | Kurtosis | | . | . |
| | PLA | Mean | | 5.0000 | .00000 |
| | | 95% Confidence Interval for Mean | Lower Bound | 5.0000 | |
| | | | Upper Bound | 5.0000 | |
| | | 5% Trimmed Mean | | 5.0000 | |
| | | Median | | 5.0000 | |
| | | Variance | | .000 | |
| | | Std. Deviation | | .00000 | |
| | | Minimum | | 5.00 | |
| | | Maximum | | 5.00 | |
| | | Range | | .00 | |
| | | Interquartile Range | | .00 | |
| | | Skewness | | . | . |
| | | Kurtosis | | . | . |
| | PLATEC0.6 | Mean | | 5.0000 | .00000 |
| | | 95% Confidence Interval for Mean | Lower Bound | 5.0000 | |
| | | | Upper Bound | 5.0000 | |
| | | 5% Trimmed Mean | | 5.0000 | |
| | | Median | | 5.0000 | |
| | | Variance | | .000 | |
| | | Std. Deviation | | .00000 | |
| | | Minimum | | 5.00 | |
| | | Maximum | | 5.00 | |
| | | Range | | .00 | |
| | | Interquartile Range | | .00 | |
| | | Skewness | | . | . |
| | | Kurtosis | | . | . |
| cohesionDAY2 | CONTROL | Mean | | 4.4800 | .16743 |
| | | 95% Confidence Interval for Mean | Lower Bound | 3.7596 | |
| | | | Upper Bound | 5.2004 | |
| | | 5% Trimmed Mean | | . | |
| | | Median | | 4.4800 | |
| | | Variance | | .084 | |
| | | Std. Deviation | | .29000 | |
| | | Minimum | | 4.19 | |
| | | Maximum | | 4.77 | |
| | | Range | | .58 | |
| | | Interquartile Range | | . | |
| | | Skewness | | .000 | 1.225 |
| | | Kurtosis | | . | . |
| | PLA | Mean | | 4.2800 | .23094 |
| | | 95% Confidence Interval for Mean | Lower Bound | 3.2863 | |

| | | | | | |
|---|---|---|---|---|---|
| | | | Upper Bound | 5.2737 | |
| | | 5% Trimmed Mean | | . | |
| | | Median | | 4.2800 | |
| | | Variance | | .160 | |
| | | Std. Deviation | | .40000 | |
| | | Minimum | | 3.88 | |
| | | Maximum | | 4.68 | |
| | | Range | | .80 | |
| | | Interquartile Range | | . | |
| | | Skewness | | .000 | 1.225 |
| | | Kurtosis | | . | . |
| | PLATEC0.6 | Mean | | 4.4600 | .15588 |
| | | 95% Confidence Interval for Mean | Lower Bound | 3.7893 | |
| | | | Upper Bound | 5.1307 | |
| | | 5% Trimmed Mean | | . | |
| | | Median | | 4.4600 | |
| | | Variance | | .073 | |
| | | Std. Deviation | | .27000 | |
| | | Minimum | | 4.19 | |
| | | Maximum | | 4.73 | |
| | | Range | | .54 | |
| | | Interquartile Range | | . | |
| | | Skewness | | .000 | 1.225 |
| | | Kurtosis | | . | . |
| cohesionDAY4 | CONTROL | Mean | | 3.8000 | .11547 |
| | | 95% Confidence Interval for Mean | Lower Bound | 3.3032 | |
| | | | Upper Bound | 4.2968 | |
| | | 5% Trimmed Mean | | . | |
| | | Median | | 3.8000 | |
| | | Variance | | .040 | |
| | | Std. Deviation | | .20000 | |
| | | Minimum | | 3.60 | |
| | | Maximum | | 4.00 | |
| | | Range | | .40 | |
| | | Interquartile Range | | . | |
| | | Skewness | | .000 | 1.225 |
| | | Kurtosis | | . | . |
| | PLA | Mean | | 4.1000 | .14434 |
| | | 95% Confidence Interval for Mean | Lower Bound | 3.4790 | |
| | | | Upper Bound | 4.7210 | |
| | | 5% Trimmed Mean | | . | |
| | | Median | | 4.1000 | |
| | | Variance | | .062 | |
| | | Std. Deviation | | .25000 | |
| | | Minimum | | 3.85 | |
| | | Maximum | | 4.35 | |
| | | Range | | .50 | |
| | | Interquartile Range | | . | |
| | | Skewness | | .000 | 1.225 |
| | | Kurtosis | | . | . |
| | PLATEC0.6 | Mean | | 4.3200 | .06351 |
| | | 95% Confidence Interval for Mean | Lower Bound | 4.0467 | |
| | | | Upper Bound | 4.5933 | |
| | | 5% Trimmed Mean | | . | |
| | | Median | | 4.3200 | |
| | | Variance | | .012 | |
| | | Std. Deviation | | .11000 | |
| | | Minimum | | 4.21 | |
| | | Maximum | | 4.43 | |
| | | Range | | .22 | |
| | | Interquartile Range | | . | |
| | | Skewness | | .000 | 1.225 |
| | | Kurtosis | | . | . |
| cohesionDAY6 | CONTROL | Mean | | 2.8400 | .06351 |
| | | 95% Confidence Interval for Mean | Lower Bound | 2.5667 | |
| | | | Upper Bound | 3.1133 | |
| | | 5% Trimmed Mean | | . | |
| | | Median | | 2.8400 | |
| | | Variance | | .012 | |
| | | Std. Deviation | | .11000 | |
| | | Minimum | | 2.73 | |

| | | | | | |
|---|---|---|---|---|---|
| | | Maximum | | 2.95 | |
| | | Range | | .22 | |
| | | Interquartile Range | | . | |
| | | Skewness | | .000 | 1.225 |
| | | Kurtosis | | . | . |
| | PLA | Mean | | 3.3200 | .16166 |
| | | 95% Confidence Interval for Mean | Lower Bound | 2.6244 | |
| | | | Upper Bound | 4.0156 | |
| | | 5% Trimmed Mean | | . | |
| | | Median | | 3.3200 | |
| | | Variance | | .078 | |
| | | Std. Deviation | | .28000 | |
| | | Minimum | | 3.04 | |
| | | Maximum | | 3.60 | |
| | | Range | | .56 | |
| | | Interquartile Range | | . | |
| | | Skewness | | .000 | 1.225 |
| | | Kurtosis | | . | . |
| | PLATEC0.6 | Mean | | 3.9400 | .10392 |
| | | 95% Confidence Interval for Mean | Lower Bound | 3.4929 | |
| | | | Upper Bound | 4.3871 | |
| | | 5% Trimmed Mean | | . | |
| | | Median | | 3.9400 | |
| | | Variance | | .032 | |
| | | Std. Deviation | | .18000 | |
| | | Minimum | | 3.76 | |
| | | Maximum | | 4.12 | |
| | | Range | | .36 | |
| | | Interquartile Range | | . | |
| | | Skewness | | .000 | 1.225 |
| | | Kurtosis | | . | . |
| colorDAY0 | CONTROL | Mean | | 5.0000 | .00000 |
| | | 95% Confidence Interval for Mean | Lower Bound | 5.0000 | |
| | | | Upper Bound | 5.0000 | |
| | | 5% Trimmed Mean | | 5.0000 | |
| | | Median | | 5.0000 | |
| | | Variance | | .000 | |
| | | Std. Deviation | | .00000 | |
| | | Minimum | | 5.00 | |
| | | Maximum | | 5.00 | |
| | | Range | | .00 | |
| | | Interquartile Range | | .00 | |
| | | Skewness | | . | . |
| | | Kurtosis | | . | . |
| | PLA | Mean | | 5.0000 | .00000 |
| | | 95% Confidence Interval for Mean | Lower Bound | 5.0000 | |
| | | | Upper Bound | 5.0000 | |
| | | 5% Trimmed Mean | | 5.0000 | |
| | | Median | | 5.0000 | |
| | | Variance | | .000 | |
| | | Std. Deviation | | .00000 | |
| | | Minimum | | 5.00 | |
| | | Maximum | | 5.00 | |
| | | Range | | .00 | |
| | | Interquartile Range | | .00 | |
| | | Skewness | | . | . |
| | | Kurtosis | | . | . |
| | PLATEC0.6 | Mean | | 5.0000 | .00000 |
| | | 95% Confidence Interval for Mean | Lower Bound | 5.0000 | |
| | | | Upper Bound | 5.0000 | |
| | | 5% Trimmed Mean | | 5.0000 | |
| | | Median | | 5.0000 | |
| | | Variance | | .000 | |
| | | Std. Deviation | | .00000 | |
| | | Minimum | | 5.00 | |
| | | Maximum | | 5.00 | |
| | | Range | | .00 | |
| | | Interquartile Range | | .00 | |
| | | Skewness | | . | . |
| | | Kurtosis | | . | . |
| colorDAY2 | CONTROL | Mean | | 4.3000 | .11547 |

| | | | | | |
|---|---|---|---|---|---|
| | | 95% Confidence Interval for Mean | Lower Bound | 3.8032 | |
| | | | Upper Bound | 4.7968 | |
| | | 5% Trimmed Mean | | . | |
| | | Median | | 4.3000 | |
| | | Variance | | .040 | |
| | | Std. Deviation | | .20000 | |
| | | Minimum | | 4.10 | |
| | | Maximum | | 4.50 | |
| | | Range | | .40 | |
| | | Interquartile Range | | . | |
| | | Skewness | | .000 | 1.225 |
| | | Kurtosis | | . | . |
| | PLA | Mean | | 4.5000 | .05774 |
| | | 95% Confidence Interval for Mean | Lower Bound | 4.2516 | |
| | | | Upper Bound | 4.7484 | |
| | | 5% Trimmed Mean | | . | |
| | | Median | | 4.5000 | |
| | | Variance | | .010 | |
| | | Std. Deviation | | .10000 | |
| | | Minimum | | 4.40 | |
| | | Maximum | | 4.60 | |
| | | Range | | .20 | |
| | | Interquartile Range | | . | |
| | | Skewness | | .000 | 1.225 |
| | | Kurtosis | | . | . |
| | PLATEC0.6 | Mean | | 4.6000 | .02887 |
| | | 95% Confidence Interval for Mean | Lower Bound | 4.4758 | |
| | | | Upper Bound | 4.7242 | |
| | | 5% Trimmed Mean | | . | |
| | | Median | | 4.6000 | |
| | | Variance | | .003 | |
| | | Std. Deviation | | .05000 | |
| | | Minimum | | 4.55 | |
| | | Maximum | | 4.65 | |
| | | Range | | .10 | |
| | | Interquartile Range | | . | |
| | | Skewness | | .000 | 1.225 |
| | | Kurtosis | | . | . |
| colorDAY4 | CONTROL | Mean | | 3.9800 | .16743 |
| | | 95% Confidence Interval for Mean | Lower Bound | 3.2596 | |
| | | | Upper Bound | 4.7004 | |
| | | 5% Trimmed Mean | | . | |
| | | Median | | 3.9800 | |
| | | Variance | | .084 | |
| | | Std. Deviation | | .29000 | |
| | | Minimum | | 3.69 | |
| | | Maximum | | 4.27 | |
| | | Range | | .58 | |
| | | Interquartile Range | | . | |
| | | Skewness | | .000 | 1.225 |
| | | Kurtosis | | . | . |
| | PLA | Mean | | 4.1200 | .19053 |
| | | 95% Confidence Interval for Mean | Lower Bound | 3.3002 | |
| | | | Upper Bound | 4.9398 | |
| | | 5% Trimmed Mean | | . | |
| | | Median | | 4.1200 | |
| | | Variance | | .109 | |
| | | Std. Deviation | | .33000 | |
| | | Minimum | | 3.79 | |
| | | Maximum | | 4.45 | |
| | | Range | | .66 | |
| | | Interquartile Range | | . | |
| | | Skewness | | .000 | 1.225 |
| | | Kurtosis | | . | . |
| | PLATEC0.6 | Mean | | 4.3500 | .15588 |
| | | 95% Confidence Interval for Mean | Lower Bound | 3.6793 | |
| | | | Upper Bound | 5.0207 | |
| | | 5% Trimmed Mean | | . | |
| | | Median | | 4.3500 | |
| | | Variance | | .073 | |
| | | Std. Deviation | | .27000 | |

| | | | | | |
|---|---|---|---|---|---|
| | | Minimum | | 4.08 | |
| | | Maximum | | 4.62 | |
| | | Range | | .54 | |
| | | Interquartile Range | | . | |
| | | Skewness | | .000 | 1.225 |
| | | Kurtosis | | . | . |
| colorDAY6 | CONTROL | Mean | | 2.8500 | .08660 |
| | | 95% Confidence Interval for Mean | Lower Bound | 2.4774 | |
| | | | Upper Bound | 3.2226 | |
| | | 5% Trimmed Mean | | . | |
| | | Median | | 2.8500 | |
| | | Variance | | .022 | |
| | | Std. Deviation | | .15000 | |
| | | Minimum | | 2.70 | |
| | | Maximum | | 3.00 | |
| | | Range | | .30 | |
| | | Interquartile Range | | . | |
| | | Skewness | | .000 | 1.225 |
| | | Kurtosis | | . | . |
| | PLA | Mean | | 3.5000 | .09238 |
| | | 95% Confidence Interval for Mean | Lower Bound | 3.1025 | |
| | | | Upper Bound | 3.8975 | |
| | | 5% Trimmed Mean | | . | |
| | | Median | | 3.5000 | |
| | | Variance | | .026 | |
| | | Std. Deviation | | .16000 | |
| | | Minimum | | 3.34 | |
| | | Maximum | | 3.66 | |
| | | Range | | .32 | |
| | | Interquartile Range | | . | |
| | | Skewness | | .000 | 1.225 |
| | | Kurtosis | | . | . |
| | PLATEC0.6 | Mean | | 4.0200 | .10970 |
| | | 95% Confidence Interval for Mean | Lower Bound | 3.5480 | |
| | | | Upper Bound | 4.4920 | |
| | | 5% Trimmed Mean | | . | |
| | | Median | | 4.0200 | |
| | | Variance | | .036 | |
| | | Std. Deviation | | .19000 | |
| | | Minimum | | 3.83 | |
| | | Maximum | | 4.21 | |
| | | Range | | .38 | |
| | | Interquartile Range | | . | |
| | | Skewness | | .000 | 1.225 |
| | | Kurtosis | | . | . |
| feDAY0 | CONTROL | Mean | | 7.6569 | .07208 |
| | | 95% Confidence Interval for Mean | Lower Bound | 7.3468 | |
| | | | Upper Bound | 7.9671 | |
| | | 5% Trimmed Mean | | . | |
| | | Median | | 7.6170 | |
| | | Variance | | .016 | |
| | | Std. Deviation | | .12485 | |
| | | Minimum | | 7.56 | |
| | | Maximum | | 7.80 | |
| | | Range | | .24 | |
| | | Interquartile Range | | . | |
| | | Skewness | | 1.293 | 1.225 |
| | | Kurtosis | | . | . |
| | PLA | Mean | | 7.6569 | .07208 |
| | | 95% Confidence Interval for Mean | Lower Bound | 7.3468 | |
| | | | Upper Bound | 7.9671 | |
| | | 5% Trimmed Mean | | . | |
| | | Median | | 7.6170 | |
| | | Variance | | .016 | |
| | | Std. Deviation | | .12485 | |
| | | Minimum | | 7.56 | |
| | | Maximum | | 7.80 | |
| | | Range | | .24 | |
| | | Interquartile Range | | . | |
| | | Skewness | | 1.293 | 1.225 |
| | | Kurtosis | | . | . |

| | | | | | |
|---|---|---|---|---|---|
| | PLATEC0.6 | Mean | | 7.6569 | .07208 |
| | | 95% Confidence Interval for Mean | Lower Bound | 7.3468 | |
| | | | Upper Bound | 7.9671 | |
| | | 5% Trimmed Mean | | . | |
| | | Median | | 7.6170 | |
| | | Variance | | .016 | |
| | | Std. Deviation | | .12485 | |
| | | Minimum | | 7.56 | |
| | | Maximum | | 7.80 | |
| | | Range | | .24 | |
| | | Interquartile Range | | . | |
| | | Skewness | | 1.293 | 1.225 |
| | | Kurtosis | | . | . |
| feDAY2 | CONTROL | Mean | | 6.2575 | .20872 |
| | | 95% Confidence Interval for Mean | Lower Bound | 5.3594 | |
| | | | Upper Bound | 7.1556 | |
| | | 5% Trimmed Mean | | . | |
| | | Median | | 6.2975 | |
| | | Variance | | .131 | |
| | | Std. Deviation | | .36152 | |
| | | Minimum | | 5.88 | |
| | | Maximum | | 6.60 | |
| | | Range | | .72 | |
| | | Interquartile Range | | . | |
| | | Skewness | | -.492 | 1.225 |
| | | Kurtosis | | . | . |
| | PLA | Mean | | 6.7373 | .12161 |
| | | 95% Confidence Interval for Mean | Lower Bound | 6.2141 | |
| | | | Upper Bound | 7.2605 | |
| | | 5% Trimmed Mean | | . | |
| | | Median | | 6.7173 | |
| | | Variance | | .044 | |
| | | Std. Deviation | | .21063 | |
| | | Minimum | | 6.54 | |
| | | Maximum | | 6.96 | |
| | | Range | | .42 | |
| | | Interquartile Range | | . | |
| | | Skewness | | .423 | 1.225 |
| | | Kurtosis | | . | . |
| | PLATEC0.6 | Mean | | 7.1172 | .07208 |
| | | 95% Confidence Interval for Mean | Lower Bound | 6.8070 | |
| | | | Upper Bound | 7.4273 | |
| | | 5% Trimmed Mean | | . | |
| | | Median | | 7.0772 | |
| | | Variance | | .016 | |
| | | Std. Deviation | | .12485 | |
| | | Minimum | | 7.02 | |
| | | Maximum | | 7.26 | |
| | | Range | | .24 | |
| | | Interquartile Range | | . | |
| | | Skewness | | 1.293 | 1.225 |
| | | Kurtosis | | . | . |
| feDAY4 | CONTROL | Mean | | 5.5178 | .10388 |
| | | 95% Confidence Interval for Mean | Lower Bound | 5.0708 | |
| | | | Upper Bound | 5.9648 | |
| | | 5% Trimmed Mean | | . | |
| | | Median | | 5.5178 | |
| | | Variance | | .032 | |
| | | Std. Deviation | | .17993 | |
| | | Minimum | | 5.34 | |
| | | Maximum | | 5.70 | |
| | | Range | | .36 | |
| | | Interquartile Range | | . | |
| | | Skewness | | .000 | 1.225 |
| | | Kurtosis | | . | . |
| | PLA | Mean | | 5.7177 | .07208 |
| | | 95% Confidence Interval for Mean | Lower Bound | 5.4076 | |
| | | | Upper Bound | 6.0279 | |
| | | 5% Trimmed Mean | | . | |
| | | Median | | 5.7577 | |
| | | Variance | | .016 | |

| | | | | | |
|---|---|---|---|---|---|
| | | Std. Deviation | | .12485 | |
| | | Minimum | | 5.58 | |
| | | Maximum | | 5.82 | |
| | | Range | | .24 | |
| | | Interquartile Range | | . | |
| | | Skewness | | -1.293 | 1.225 |
| | | Kurtosis | | . | . |
| | PLATEC0.6 | Mean | | 6.0976 | .12161 |
| | | 95% Confidence Interval for Mean | Lower Bound | 5.5743 | |
| | | | Upper Bound | 6.6208 | |
| | | 5% Trimmed Mean | | . | |
| | | Median | | 6.1176 | |
| | | Variance | | .044 | |
| | | Std. Deviation | | .21063 | |
| | | Minimum | | 5.88 | |
| | | Maximum | | 6.30 | |
| | | Range | | .42 | |
| | | Interquartile Range | | . | |
| | | Skewness | | -.423 | 1.225 |
| | | Kurtosis | | . | . |
| feDAY6 | CONTROL | Mean | | 4.6981 | .19071 |
| | | 95% Confidence Interval for Mean | Lower Bound | 3.8776 | |
| | | | Upper Bound | 5.5187 | |
| | | 5% Trimmed Mean | | . | |
| | | Median | | 4.6781 | |
| | | Variance | | .109 | |
| | | Std. Deviation | | .33032 | |
| | | Minimum | | 4.38 | |
| | | Maximum | | 5.04 | |
| | | Range | | .66 | |
| | | Interquartile Range | | . | |
| | | Skewness | | .271 | 1.225 |
| | | Kurtosis | | . | . |
| | PLA | Mean | | 4.6381 | .11131 |
| | | 95% Confidence Interval for Mean | Lower Bound | 4.1592 | |
| | | | Upper Bound | 5.1171 | |
| | | 5% Trimmed Mean | | . | |
| | | Median | | 4.5582 | |
| | | Variance | | .037 | |
| | | Std. Deviation | | .19280 | |
| | | Minimum | | 4.50 | |
| | | Maximum | | 4.86 | |
| | | Range | | .36 | |
| | | Interquartile Range | | . | |
| | | Skewness | | 1.545 | 1.225 |
| | | Kurtosis | | . | . |
| | PLATEC0.6 | Mean | | 5.1979 | .15614 |
| | | 95% Confidence Interval for Mean | Lower Bound | 4.5261 | |
| | | | Upper Bound | 5.8697 | |
| | | 5% Trimmed Mean | | . | |
| | | Median | | 5.2179 | |
| | | Variance | | .073 | |
| | | Std. Deviation | | .27045 | |
| | | Minimum | | 4.92 | |
| | | Maximum | | 5.46 | |
| | | Range | | .54 | |
| | | Interquartile Range | | . | |
| | | Skewness | | -.331 | 1.225 |
| | | Kurtosis | | . | . |
| odorDAY0 | CONTROL | Mean | | 5.0000 | .00000 |
| | | 95% Confidence Interval for Mean | Lower Bound | 5.0000 | |
| | | | Upper Bound | 5.0000 | |
| | | 5% Trimmed Mean | | 5.0000 | |
| | | Median | | 5.0000 | |
| | | Variance | | .000 | |
| | | Std. Deviation | | .00000 | |
| | | Minimum | | 5.00 | |
| | | Maximum | | 5.00 | |
| | | Range | | .00 | |
| | | Interquartile Range | | .00 | |
| | | Skewness | | . | . |

| | | | | | |
|---|---|---|---|---|---|
| | | Kurtosis | | . | . |
| | PLA | Mean | | 5.0000 | .00000 |
| | | 95% Confidence Interval for Mean | Lower Bound | 5.0000 | |
| | | | Upper Bound | 5.0000 | |
| | | 5% Trimmed Mean | | 5.0000 | |
| | | Median | | 5.0000 | |
| | | Variance | | .000 | |
| | | Std. Deviation | | .00000 | |
| | | Minimum | | 5.00 | |
| | | Maximum | | 5.00 | |
| | | Range | | .00 | |
| | | Interquartile Range | | .00 | |
| | | Skewness | | . | . |
| | | Kurtosis | | . | . |
| | PLATEC0.6 | Mean | | 5.0000 | .00000 |
| | | 95% Confidence Interval for Mean | Lower Bound | 5.0000 | |
| | | | Upper Bound | 5.0000 | |
| | | 5% Trimmed Mean | | 5.0000 | |
| | | Median | | 5.0000 | |
| | | Variance | | .000 | |
| | | Std. Deviation | | .00000 | |
| | | Minimum | | 5.00 | |
| | | Maximum | | 5.00 | |
| | | Range | | .00 | |
| | | Interquartile Range | | .00 | |
| | | Skewness | | . | . |
| | | Kurtosis | | . | . |
| odorDAY2 | CONTROL | Mean | | 4.3200 | .13856 |
| | | 95% Confidence Interval for Mean | Lower Bound | 3.7238 | |
| | | | Upper Bound | 4.9162 | |
| | | 5% Trimmed Mean | | . | |
| | | Median | | 4.3200 | |
| | | Variance | | .058 | |
| | | Std. Deviation | | .24000 | |
| | | Minimum | | 4.08 | |
| | | Maximum | | 4.56 | |
| | | Range | | .48 | |
| | | Interquartile Range | | . | |
| | | Skewness | | .000 | 1.225 |
| | | Kurtosis | | . | . |
| | PLA | Mean | | 4.5500 | .08083 |
| | | 95% Confidence Interval for Mean | Lower Bound | 4.2022 | |
| | | | Upper Bound | 4.8978 | |
| | | 5% Trimmed Mean | | . | |
| | | Median | | 4.5500 | |
| | | Variance | | .020 | |
| | | Std. Deviation | | .14000 | |
| | | Minimum | | 4.41 | |
| | | Maximum | | 4.69 | |
| | | Range | | .28 | |
| | | Interquartile Range | | . | |
| | | Skewness | | .000 | 1.225 |
| | | Kurtosis | | . | . |
| | PLATEC0.6 | Mean | | 4.7100 | .08083 |
| | | 95% Confidence Interval for Mean | Lower Bound | 4.3622 | |
| | | | Upper Bound | 5.0578 | |
| | | 5% Trimmed Mean | | . | |
| | | Median | | 4.7100 | |
| | | Variance | | .020 | |
| | | Std. Deviation | | .14000 | |
| | | Minimum | | 4.57 | |
| | | Maximum | | 4.85 | |
| | | Range | | .28 | |
| | | Interquartile Range | | . | |
| | | Skewness | | .000 | 1.225 |
| | | Kurtosis | | . | . |
| odorDAY4 | CONTROL | Mean | | 3.9400 | .13279 |
| | | 95% Confidence Interval for Mean | Lower Bound | 3.3686 | |
| | | | Upper Bound | 4.5114 | |
| | | 5% Trimmed Mean | | . | |
| | | Median | | 3.9400 | |

| | | | | | |
|---|---|---|---|---|---|
| | | Variance | | .053 | |
| | | Std. Deviation | | .23000 | |
| | | Minimum | | 3.71 | |
| | | Maximum | | 4.17 | |
| | | Range | | .46 | |
| | | Interquartile Range | | . | |
| | | Skewness | | .000 | 1.225 |
| | | Kurtosis | | . | . |
| | PLA | Mean | | 4.2200 | .15588 |
| | | 95% Confidence Interval for Mean | Lower Bound | 3.5493 | |
| | | | Upper Bound | 4.8907 | |
| | | 5% Trimmed Mean | | . | |
| | | Median | | 4.2200 | |
| | | Variance | | .073 | |
| | | Std. Deviation | | .27000 | |
| | | Minimum | | 3.95 | |
| | | Maximum | | 4.49 | |
| | | Range | | .54 | |
| | | Interquartile Range | | . | |
| | | Skewness | | .000 | 1.225 |
| | | Kurtosis | | . | . |
| | PLATEC0.6 | Mean | | 4.5000 | .08660 |
| | | 95% Confidence Interval for Mean | Lower Bound | 4.1274 | |
| | | | Upper Bound | 4.8726 | |
| | | 5% Trimmed Mean | | . | |
| | | Median | | 4.5000 | |
| | | Variance | | .023 | |
| | | Std. Deviation | | .15000 | |
| | | Minimum | | 4.35 | |
| | | Maximum | | 4.65 | |
| | | Range | | .30 | |
| | | Interquartile Range | | . | |
| | | Skewness | | .000 | 1.225 |
| | | Kurtosis | | . | . |
| odorDAY6 | CONTROL | Mean | | 3.5000 | .05774 |
| | | 95% Confidence Interval for Mean | Lower Bound | 3.2516 | |
| | | | Upper Bound | 3.7484 | |
| | | 5% Trimmed Mean | | . | |
| | | Median | | 3.5000 | |
| | | Variance | | .010 | |
| | | Std. Deviation | | .10000 | |
| | | Minimum | | 3.40 | |
| | | Maximum | | 3.60 | |
| | | Range | | .20 | |
| | | Interquartile Range | | . | |
| | | Skewness | | .000 | 1.225 |
| | | Kurtosis | | . | . |
| | PLA | Mean | | 3.6000 | .11547 |
| | | 95% Confidence Interval for Mean | Lower Bound | 3.1032 | |
| | | | Upper Bound | 4.0968 | |
| | | 5% Trimmed Mean | | . | |
| | | Median | | 3.6000 | |
| | | Variance | | .040 | |
| | | Std. Deviation | | .20000 | |
| | | Minimum | | 3.40 | |
| | | Maximum | | 3.80 | |
| | | Range | | .40 | |
| | | Interquartile Range | | . | |
| | | Skewness | | .000 | 1.225 |
| | | Kurtosis | | . | . |
| | PLATEC0.6 | Mean | | 4.0500 | .09238 |
| | | 95% Confidence Interval for Mean | Lower Bound | 3.6525 | |
| | | | Upper Bound | 4.4475 | |
| | | 5% Trimmed Mean | | . | |
| | | Median | | 4.0500 | |
| | | Variance | | .026 | |
| | | Std. Deviation | | .16000 | |
| | | Minimum | | 3.89 | |
| | | Maximum | | 4.21 | |
| | | Range | | .32 | |
| | | Interquartile Range | | . | |

| | | | | | |
|---|---|---|---|---|---|
| | | Skewness | | .000 | 1.225 |
| | | Kurtosis | | . | . |
| TBA_DAY0 | CONTROL | Mean | | .4608 | .00831 |
| | | 95% Confidence Interval for Mean | Lower Bound | .4250 | |
| | | | Upper Bound | .4966 | |
| | | 5% Trimmed Mean | | . | |
| | | Median | | .4608 | |
| | | Variance | | .000 | |
| | | Std. Deviation | | .01440 | |
| | | Minimum | | .45 | |
| | | Maximum | | .48 | |
| | | Range | | .03 | |
| | | Interquartile Range | | . | |
| | | Skewness | | .000 | 1.225 |
| | | Kurtosis | | . | . |
| | PLA | Mean | | .4608 | .00831 |
| | | 95% Confidence Interval for Mean | Lower Bound | .4250 | |
| | | | Upper Bound | .4966 | |
| | | 5% Trimmed Mean | | . | |
| | | Median | | .4608 | |
| | | Variance | | .000 | |
| | | Std. Deviation | | .01440 | |
| | | Minimum | | .45 | |
| | | Maximum | | .48 | |
| | | Range | | .03 | |
| | | Interquartile Range | | . | |
| | | Skewness | | .000 | 1.225 |
| | | Kurtosis | | . | . |
| | PLATEC0.6 | Mean | | .4608 | .00831 |
| | | 95% Confidence Interval for Mean | Lower Bound | .4250 | |
| | | | Upper Bound | .4966 | |
| | | 5% Trimmed Mean | | . | |
| | | Median | | .4608 | |
| | | Variance | | .000 | |
| | | Std. Deviation | | .01440 | |
| | | Minimum | | .45 | |
| | | Maximum | | .48 | |
| | | Range | | .03 | |
| | | Interquartile Range | | . | |
| | | Skewness | | .000 | 1.225 |
| | | Kurtosis | | . | . |
| TBA_DAY2 | CONTROL | Mean | | .5868 | .01157 |
| | | 95% Confidence Interval for Mean | Lower Bound | .5370 | |
| | | | Upper Bound | .6366 | |
| | | 5% Trimmed Mean | | . | |
| | | Median | | .5832 | |
| | | Variance | | .000 | |
| | | Std. Deviation | | .02004 | |
| | | Minimum | | .57 | |
| | | Maximum | | .61 | |
| | | Range | | .04 | |
| | | Interquartile Range | | . | |
| | | Skewness | | .782 | 1.225 |
| | | Kurtosis | | . | . |
| | PLA | Mean | | .5760 | .00749 |
| | | 95% Confidence Interval for Mean | Lower Bound | .5438 | |
| | | | Upper Bound | .6082 | |
| | | 5% Trimmed Mean | | . | |
| | | Median | | .5724 | |
| | | Variance | | .000 | |
| | | Std. Deviation | | .01298 | |
| | | Minimum | | .57 | |
| | | Maximum | | .59 | |
| | | Range | | .03 | |
| | | Interquartile Range | | . | |
| | | Skewness | | 1.152 | 1.225 |
| | | Kurtosis | | . | . |
| | PLATEC0.6 | Mean | | .5472 | .00624 |
| | | 95% Confidence Interval for Mean | Lower Bound | .5204 | |
| | | | Upper Bound | .5740 | |
| | | 5% Trimmed Mean | | . | |

| | | | | | |
|---|---|---|---|---|---|
| | | Median | | .5472 | |
| | | Variance | | .000 | |
| | | Std. Deviation | | .01080 | |
| | | Minimum | | .54 | |
| | | Maximum | | .56 | |
| | | Range | | .02 | |
| | | Interquartile Range | | . | |
| | | Skewness | | .000 | 1.225 |
| | | Kurtosis | | . | . |
| TBA_DAY4 | CONTROL | Mean | | .7524 | .01247 |
| | | 95% Confidence Interval for Mean | Lower Bound | .6987 | |
| | | | Upper Bound | .8061 | |
| | | 5% Trimmed Mean | | . | |
| | | Median | | .7524 | |
| | | Variance | | .000 | |
| | | Std. Deviation | | .02160 | |
| | | Minimum | | .73 | |
| | | Maximum | | .77 | |
| | | Range | | .04 | |
| | | Interquartile Range | | . | |
| | | Skewness | | .000 | 1.225 |
| | | Kurtosis | | . | . |
| | PLA | Mean | | .7116 | .00937 |
| | | 95% Confidence Interval for Mean | Lower Bound | .6713 | |
| | | | Upper Bound | .7519 | |
| | | 5% Trimmed Mean | | . | |
| | | Median | | .7128 | |
| | | Variance | | .000 | |
| | | Std. Deviation | | .01623 | |
| | | Minimum | | .69 | |
| | | Maximum | | .73 | |
| | | Range | | .03 | |
| | | Interquartile Range | | . | |
| | | Skewness | | -.331 | 1.225 |
| | | Kurtosis | | . | . |
| | PLATEC0.6 | Mean | | .6564 | .00865 |
| | | 95% Confidence Interval for Mean | Lower Bound | .6192 | |
| | | | Upper Bound | .6936 | |
| | | 5% Trimmed Mean | | . | |
| | | Median | | .6516 | |
| | | Variance | | .000 | |
| | | Std. Deviation | | .01499 | |
| | | Minimum | | .64 | |
| | | Maximum | | .67 | |
| | | Range | | .03 | |
| | | Interquartile Range | | . | |
| | | Skewness | | 1.293 | 1.225 |
| | | Kurtosis | | . | . |
| TBA_DAY6 | CONTROL | Mean | | .8124 | .01182 |
| | | 95% Confidence Interval for Mean | Lower Bound | .7615 | |
| | | | Upper Bound | .8633 | |
| | | 5% Trimmed Mean | | . | |
| | | Median | | .8064 | |
| | | Variance | | .000 | |
| | | Std. Deviation | | .02047 | |
| | | Minimum | | .80 | |
| | | Maximum | | .84 | |
| | | Range | | .04 | |
| | | Interquartile Range | | . | |
| | | Skewness | | 1.206 | 1.225 |
| | | Kurtosis | | . | . |
| | PLA | Mean | | .8016 | .00840 |
| | | 95% Confidence Interval for Mean | Lower Bound | .7655 | |
| | | | Upper Bound | .8377 | |
| | | 5% Trimmed Mean | | . | |
| | | Median | | .7992 | |
| | | Variance | | .000 | |
| | | Std. Deviation | | .01455 | |
| | | Minimum | | .79 | |
| | | Maximum | | .82 | |
| | | Range | | .03 | |

| | | | | | |
|---|---|---|---|---|---|
| | | Interquartile Range | | . | |
| | | Skewness | | .722 | 1.225 |
| | | Kurtosis | | . | . |
| | PLATEC0.6 | Mean | | .7560 | .00550 |
| | | 95% Confidence Interval for Mean | Lower Bound | .7323 | |
| | | | Upper Bound | .7797 | |
| | | 5% Trimmed Mean | | . | |
| | | Median | | .7524 | |
| | | Variance | | .000 | |
| | | Std. Deviation | | .00952 | |
| | | Minimum | | .75 | |
| | | Maximum | | .77 | |
| | | Range | | .02 | |
| | | Interquartile Range | | . | |
| | | Skewness | | 1.458 | 1.225 |
| | | Kurtosis | | . | . |

a. There are no valid cases for cohesionDAY0 when Film_group3 = .. Statistics cannot be computed for this level.
b. There are no valid cases for cohesionDAY2 when Film_group3 = .. Statistics cannot be computed for this level.
c. There are no valid cases for cohesionDAY4 when Film_group3 = .. Statistics cannot be computed for this level.
d. There are no valid cases for cohesionDAY6 when Film_group3 = .. Statistics cannot be computed for this level.
e. There are no valid cases for colorDAY0 when Film_group3 = .. Statistics cannot be computed for this level.
f. There are no valid cases for colorDAY2 when Film_group3 = .. Statistics cannot be computed for this level.
g. There are no valid cases for colorDAY4 when Film_group3 = .. Statistics cannot be computed for this level.
h. There are no valid cases for colorDAY6 when Film_group3 = .. Statistics cannot be computed for this level.
i. There are no valid cases for feDAY0 when Film_group3 = .. Statistics cannot be computed for this level.
j. There are no valid cases for feDAY2 when Film_group3 = .. Statistics cannot be computed for this level.
k. There are no valid cases for feDAY4 when Film_group3 = .. Statistics cannot be computed for this level.
l. There are no valid cases for feDAY6 when Film_group3 = .. Statistics cannot be computed for this level.
m. There are no valid cases for odorDAY0 when Film_group3 = .. Statistics cannot be computed for this level.
n. There are no valid cases for odorDAY2 when Film_group3 = .. Statistics cannot be computed for this level.
o. There are no valid cases for odorDAY4 when Film_group3 = .. Statistics cannot be computed for this level.
p. There are no valid cases for odorDAY6 when Film_group3 = .. Statistics cannot be computed for this level.
q. There are no valid cases for TBA_DAY0 when Film_group3 = .. Statistics cannot be computed for this level.
r. There are no valid cases for TBA_DAY2 when Film_group3 = .. Statistics cannot be computed for this level.
s. There are no valid cases for TBA_DAY4 when Film_group3 = .. Statistics cannot be computed for this level.
t. There are no valid cases for TBA_DAY6 when Film_group3 = .. Statistics cannot be computed for this level.

**Table S13:** Overall mean values significant difference hypothesis test using Median Test results for cohesion, color, heme-iron, odor, and TBARS parameters at DAY 0, DAY 2, DAY 4, and DAY 6.

Hypothesis Test Summary

| | Null Hypothesis | Test | Sig.[b,c] | Decision |
|---|---|---|---|---|
| 1 | The medians of cohesionDAY0 are the same across categories of Film_group3. | Independent-Samples Median Test | .[a] | Unable to compute. |
| 2 | The medians of cohesionDAY2 are the same across categories of Film_group3. | Independent-Samples Median Test | .638 | Retain the null hypothesis. |
| 3 | The medians of cohesionDAY4 are the same across categories of Film_group3. | Independent-Samples Median Test | .043 | Reject the null hypothesis. |
| 4 | The medians of cohesionDAY6 are the same across categories of Film_group3. | Independent-Samples Median Test | .043 | Reject the null hypothesis. |
| 5 | The medians of colorDAY0 are the same across categories of Film_group3. | Independent-Samples Median Test | .[a] | Unable to compute. |
| 6 | The medians of colorDAY2 are the same across categories of Film_group3. | Independent-Samples Median Test | .043 | Reject the null hypothesis. |
| 7 | The medians of colorDAY4 are the same across categories of Film_group3. | Independent-Samples Median Test | .638 | Retain the null hypothesis. |
| 8 | The medians of colorDAY6 are the same across categories of Film_group3. | Independent-Samples Median Test | .043 | Reject the null hypothesis. |
| 9 | The medians of feDAY0 are the same across categories of Film_group3. | Independent-Samples Median Test | 1.000 | Retain the null hypothesis. |
| 10 | The medians of feDAY2 are the same across categories of Film_group3. | Independent-Samples Median Test | .043 | Reject the null hypothesis. |
| 11 | The medians of feDAY4 are the same across categories of Film_group3. | Independent-Samples Median Test | .043 | Reject the null hypothesis. |
| 12 | The medians of feDAY6 are the same across categories of Film_group3. | Independent-Samples Median Test | .043 | Reject the null hypothesis. |
| 13 | The medians of odorDAY0 are the same across categories of Film_group3. | Independent-Samples Median Test | .[a] | Unable to compute. |
| 14 | The medians of odorDAY2 are the same across categories of Film_group3. | Independent-Samples Median Test | .043 | Reject the null hypothesis. |
| 15 | The medians of odorDAY4 are the same across categories of Film_group3. | Independent-Samples Median Test | .043 | Reject the null hypothesis. |
| 16 | The medians of odorDAY6 are the same across categories of Film_group3. | Independent-Samples Median Test | .043 | Reject the null hypothesis. |
| 17 | The medians of TBA_DAY0 are the same across categories of Film_group3. | Independent-Samples Median Test | 1.000 | Retain the null hypothesis. |
| 18 | The medians of TBA_DAY2 are the same across categories of Film_group3. | Independent-Samples Median Test | .165 | Retain the null hypothesis. |
| 19 | The medians of TBA_DAY4 are the same across categories of Film_group3. | Independent-Samples Median Test | .043 | Reject the null hypothesis. |
| 20 | The medians of TBA_DAY6 are the same across categories of Film_group3. | Independent-Samples Median Test | .165 | Retain the null hypothesis. |

a. All test field values are less than or equal to the median.
b. The significance level is .050.
c. Asymptotic significance is displayed.

**Table S14:** Pairwise mean values significant difference hypothesis test using Median Test results for TBARS parameter at DAY 0, DAY 2, DAY 4, and DAY 6.

Pairwise Comparisons of Film_group3 DAY 0

| Sample 1-Sample 2 | Test Statistic | Sig. | Adj. Sig.[a] |
|---|---|---|---|
| CONTROL-PLA | .000 | 1.000 | 1.000 |
| CONTROL-PLATEC0.6 | .000 | 1.000 | 1.000 |

| | | | |
|---|---|---|---|
| PLA-PLATEC0.6 | .000 | 1.000 | 1.000 |

Pairwise Comparisons of Film_group3 DAY 2

| Sample 1-Sample 2 | Test Statistic | Sig. | Adj. Sig.[a] |
|---|---|---|---|
| PLATEC0.6-PLA | 6.000 | .014 | .043 |
| PLATEC0.6-CONTROL | 6.000 | .014 | .043 |
| PLA-CONTROL | .667 | .414 | 1.000 |

Pairwise Comparisons of Film_group3 DAY 4

| Sample 1-Sample 2 | Test Statistic | Sig. | Adj. Sig.[a] |
|---|---|---|---|
| PLATEC0.6-PLA | 6.000 | .014 | .043 |
| PLATEC0.6-CONTROL | 6.000 | .014 | .043 |
| PLA-CONTROL | 6.000 | .014 | .043 |

Pairwise Comparisons of Film_group3 DAY 6

| Sample 1-Sample 2 | Test Statistic | Sig. | Adj. Sig.[a] |
|---|---|---|---|
| PLATEC0.6-PLA | 6.000 | .014 | .043 |
| PLATEC0.6-CONTROL | 6.000 | .014 | .043 |
| PLA-CONTROL | .667 | .414 | 1.000 |

Each row tests the null hypothesis that the Sample 1 and Sample 2 distributions are the same.
Asymptotic significances (2-sided tests) are displayed. The significance level is .050.
a. Significance values have been adjusted by the Bonferroni correction for multiple tests.

**Table S15:** Pairwise mean values significant difference hypothesis test using Median Test results for Heme-Iron parameter at DAY 0, DAY 2, DAY 4, and DAY 6.

Pairwise Comparisons of Film_group3 DAY 0

| Sample 1-Sample 2 | Test Statistic | Sig. | Adj. Sig.[a] |
|---|---|---|---|
| CONTROL-PLA | .000 | 1.000 | 1.000 |
| CONTROL-PLATEC0.6 | .000 | 1.000 | 1.000 |
| PLA-PLATEC0.6 | .000 | 1.000 | 1.000 |

Pairwise Comparisons of Film_group3 DAY 2

| Sample 1-Sample 2 | Test Statistic | Sig. | Adj. Sig.[a] |
|---|---|---|---|
| CONTROL-PLA | .667 | .414 | 1.000 |
| CONTROL-PLATEC0.6 | 6.000 | .014 | .043 |
| PLA-PLATEC0.6 | 6.000 | .014 | .043 |

Pairwise Comparisons of Film_group3 DAY 4

| Sample 1-Sample 2 | Test Statistic | Sig. | Adj. Sig.[a] |
|---|---|---|---|
| CONTROL-PLA | .667 | .414 | 1.000 |
| CONTROL-PLATEC0.6 | 6.000 | .014 | .043 |
| PLA-PLATEC0.6 | 6.000 | .014 | .043 |

Pairwise Comparisons of Film_group3 DAY 6

| Sample 1-Sample 2 | Test Statistic | Sig. | Adj. Sig.[a] |
|---|---|---|---|
| PLA-CONTROL | .667 | .414 | 1.000 |
| PLA-PLATEC0.6 | 6.000 | .014 | .043 |
| CONTROL-PLATEC0.6 | .667 | .414 | 1.000 |

Each row tests the null hypothesis that the Sample 1 and Sample 2 distributions are the same.
Asymptotic significances (2-sided tests) are displayed. The significance level is .050.
a. Significance values have been adjusted by the Bonferroni correction for multiple tests.

**Table S16:** Correlations between thiobarbituric acid reactive substances (TBARS) and Heme iron content.

| | | DAY0 | DAY2 | DAY4 | DAY6 |
|---|---|---|---|---|---|
| DAY0 | Pearson Correlation | 1 | .357 | .378 | .389 |
| | Sig. (2-tailed) | | .146 | .122 | .111 |
| | N | 18 | 18 | 18 | 18 |
| DAY2 | Pearson Correlation | .357 | 1 | .997** | .997** |
| | Sig. (2-tailed) | .146 | | .000 | .000 |
| | N | 18 | 18 | 18 | 18 |
| DAY4 | Pearson Correlation | .378 | .997** | 1 | .997** |
| | Sig. (2-tailed) | .122 | .000 | | .000 |
| | N | 18 | 18 | 18 | 18 |
| DAY6 | Pearson Correlation | .389 | .997** | .997** | 1 |
| | Sig. (2-tailed) | .111 | .000 | .000 | |
| | N | 18 | 18 | 18 | 18 |

**. Correlation is significant at the 0.01 level (2-tailed).

**Table S17:** Description statistics of TVC from DAY 0 to DAY 6

**Descriptives[a,b,c,d]**

| | Film_group3 | | | Statistic | Std. Error |
|---|---|---|---|---|---|
| TVC_DAY0 | CONTROL | Mean | | 4.3800 | .01732 |
| | | 95% Confidence Interval for Mean | Lower Bound | 4.3055 | |
| | | | Upper Bound | 4.4545 | |
| | | 5% Trimmed Mean | | . | |

| | | | | | |
|---|---|---|---|---|---|
| | | Median | | 4.3800 | |
| | | Variance | | .001 | |
| | | Std. Deviation | | .03000 | |
| | | Minimum | | 4.35 | |
| | | Maximum | | 4.41 | |
| | | Range | | .06 | |
| | | Interquartile Range | | . | |
| | | Skewness | | .000 | 1.225 |
| | | Kurtosis | | . | . |
| | PLA | Mean | | 4.3800 | .01732 |
| | | 95% Confidence Interval for Mean | Lower Bound | 4.3055 | |
| | | | Upper Bound | 4.4545 | |
| | | 5% Trimmed Mean | | . | |
| | | Median | | 4.3800 | |
| | | Variance | | .001 | |
| | | Std. Deviation | | .03000 | |
| | | Minimum | | 4.35 | |
| | | Maximum | | 4.41 | |
| | | Range | | .06 | |
| | | Interquartile Range | | . | |
| | | Skewness | | .000 | 1.225 |
| | | Kurtosis | | . | . |
| | PLATEC0.6 | Mean | | 4.3800 | .01732 |
| | | 95% Confidence Interval for Mean | Lower Bound | 4.3055 | |
| | | | Upper Bound | 4.4545 | |
| | | 5% Trimmed Mean | | . | |
| | | Median | | 4.3800 | |
| | | Variance | | .001 | |
| | | Std. Deviation | | .03000 | |
| | | Minimum | | 4.35 | |
| | | Maximum | | 4.41 | |
| | | Range | | .06 | |
| | | Interquartile Range | | . | |
| | | Skewness | | .000 | 1.225 |
| | | Kurtosis | | . | . |
| TVC_DAY2 | CONTROL | Mean | | 5.5567 | .07796 |
| | | 95% Confidence Interval for Mean | Lower Bound | 5.2212 | |
| | | | Upper Bound | 5.8921 | |
| | | 5% Trimmed Mean | | . | |
| | | Median | | 5.5600 | |
| | | Variance | | .018 | |
| | | Std. Deviation | | .13503 | |
| | | Minimum | | 5.42 | |
| | | Maximum | | 5.69 | |
| | | Range | | .27 | |
| | | Interquartile Range | | . | |
| | | Skewness | | -.111 | 1.225 |
| | | Kurtosis | | . | . |
| | PLA | Mean | | 5.2667 | .09528 |
| | | 95% Confidence Interval for Mean | Lower Bound | 4.8567 | |
| | | | Upper Bound | 5.6766 | |
| | | 5% Trimmed Mean | | . | |
| | | Median | | 5.2700 | |
| | | Variance | | .027 | |
| | | Std. Deviation | | .16503 | |
| | | Minimum | | 5.10 | |
| | | Maximum | | 5.43 | |
| | | Range | | .33 | |
| | | Interquartile Range | | . | |
| | | Skewness | | -.091 | 1.225 |
| | | Kurtosis | | . | . |
| | PLATEC0.6 | Mean | | 5.0800 | .02309 |
| | | 95% Confidence Interval for Mean | Lower Bound | 4.9806 | |
| | | | Upper Bound | 5.1794 | |
| | | 5% Trimmed Mean | | . | |
| | | Median | | 5.0800 | |
| | | Variance | | .002 | |
| | | Std. Deviation | | .04000 | |
| | | Minimum | | 5.04 | |
| | | Maximum | | 5.12 | |
| | | Range | | .08 | |

| | | | | | |
|---|---|---|---|---|---|
| | | Interquartile Range | | . | |
| | | Skewness | | .000 | 1.225 |
| | | Kurtosis | | . | . |
| TVC_DAY4 | CONTROL | Mean | | 6.8367 | .03756 |
| | | 95% Confidence Interval for Mean | Lower Bound | 6.6750 | |
| | | | Upper Bound | 6.9983 | |
| | | 5% Trimmed Mean | | . | |
| | | Median | | 6.8400 | |
| | | Variance | | .004 | |
| | | Std. Deviation | | .06506 | |
| | | Minimum | | 6.77 | |
| | | Maximum | | 6.90 | |
| | | Range | | .13 | |
| | | Interquartile Range | | . | |
| | | Skewness | | -.230 | 1.225 |
| | | Kurtosis | | . | . |
| | PLA | Mean | | 6.4767 | .03756 |
| | | 95% Confidence Interval for Mean | Lower Bound | 6.3150 | |
| | | | Upper Bound | 6.6383 | |
| | | 5% Trimmed Mean | | . | |
| | | Median | | 6.4800 | |
| | | Variance | | .004 | |
| | | Std. Deviation | | .06506 | |
| | | Minimum | | 6.41 | |
| | | Maximum | | 6.54 | |
| | | Range | | .13 | |
| | | Interquartile Range | | . | |
| | | Skewness | | -.230 | 1.225 |
| | | Kurtosis | | . | . |
| | PLATEC0.6 | Mean | | 6.3100 | .04041 |
| | | 95% Confidence Interval for Mean | Lower Bound | 6.1361 | |
| | | | Upper Bound | 6.4839 | |
| | | 5% Trimmed Mean | | . | |
| | | Median | | 6.3100 | |
| | | Variance | | .005 | |
| | | Std. Deviation | | .07000 | |
| | | Minimum | | 6.24 | |
| | | Maximum | | 6.38 | |
| | | Range | | .14 | |
| | | Interquartile Range | | . | |
| | | Skewness | | .000 | 1.225 |
| | | Kurtosis | | . | . |
| TVC_DAY6 | CONTROL | Mean | | 8.0400 | .00577 |
| | | 95% Confidence Interval for Mean | Lower Bound | 8.0152 | |
| | | | Upper Bound | 8.0648 | |
| | | 5% Trimmed Mean | | . | |
| | | Median | | 8.0400 | |
| | | Variance | | .000 | |
| | | Std. Deviation | | .01000 | |
| | | Minimum | | 8.03 | |
| | | Maximum | | 8.05 | |
| | | Range | | .02 | |
| | | Interquartile Range | | . | |
| | | Skewness | | .000 | 1.225 |
| | | Kurtosis | | . | . |
| | PLA | Mean | | 7.5067 | .02028 |
| | | 95% Confidence Interval for Mean | Lower Bound | 7.4194 | |
| | | | Upper Bound | 7.5939 | |
| | | 5% Trimmed Mean | | . | |
| | | Median | | 7.5100 | |
| | | Variance | | .001 | |
| | | Std. Deviation | | .03512 | |
| | | Minimum | | 7.47 | |
| | | Maximum | | 7.54 | |
| | | Range | | .07 | |
| | | Interquartile Range | | . | |
| | | Skewness | | -.423 | 1.225 |
| | | Kurtosis | | . | . |
| | PLATEC0.6 | Mean | | 7.3567 | .02603 |
| | | 95% Confidence Interval for Mean | Lower Bound | 7.2447 | |
| | | | Upper Bound | 7.4687 | |

| | | | |
|---|---|---|---|
| | 5% Trimmed Mean | . | |
| | Median | 7.3600 | |
| | Variance | .002 | |
| | Std. Deviation | .04509 | |
| | Minimum | 7.31 | |
| | Maximum | 7.40 | |
| | Range | .09 | |
| | Interquartile Range | . | |
| | Skewness | -.331 | 1.225 |
| | Kurtosis | . | . |

a. There are no valid cases for TVC_DAY0 when Film_group3 = .. Statistics cannot be computed for this level.
b. There are no valid cases for TVC_DAY2 when Film_group3 = .. Statistics cannot be computed for this level.
c. There are no valid cases for TVC_DAY4 when Film_group3 = .. Statistics cannot be computed for this level.
d. There are no valid cases for TVC_DAY6 when Film_group3 = .. Statistics cannot be computed for this level.

**Table S18:** Overall mean values significant difference hypothesis test using Median Test results for TVC parameter at DAY 0, DAY 2, DAY 4, and DAY 6.

Hypothesis Test Summary

| | Null Hypothesis | Test | Sig.[a,b] | Decision |
|---|---|---|---|---|
| 1 | The medians of TVC_DAY0 are the same across categories of Film_group3. | Independent-Samples Median Test | 1.000 | Retain the null hypothesis. |
| 2 | The medians of TVC_DAY2 are the same across categories of Film_group3. | Independent-Samples Median Test | .043 | Reject the null hypothesis. |
| 3 | The medians of TVC_DAY4 are the same across categories of Film_group3. | Independent-Samples Median Test | .043 | Reject the null hypothesis. |
| 4 | The medians of TVC_DAY6 are the same across categories of Film_group3. | Independent-Samples Median Test | .043 | Reject the null hypothesis. |

a. The significance level is .050.
b. Asymptotic significance is displayed.

**Table S19:** Pairwise mean values significant difference hypothesis test using Median Test results for TVC parameter at DAY 0, DAY 2, DAY 4, and DAY 6.

Pairwise Comparisons of Film_group3 DAY 0

| Sample 1-Sample 2 | Test Statistic | Sig. | Adj. Sig.[a] |
|---|---|---|---|
| CONTROL-PLA | .000 | 1.000 | 1.000 |
| CONTROL-PLATEC0.6 | .000 | 1.000 | 1.000 |
| PLA-PLATEC0.6 | .000 | 1.000 | 1.000 |

Pairwise Comparisons of Film_group3 DAY 2

| Sample 1-Sample 2 | Test Statistic | Sig. | Adj. Sig.[a] |
|---|---|---|---|
| PLATEC0.6-PLA | .667 | .414 | 1.000 |
| PLATEC0.6-CONTROL | 6.000 | .014 | .043 |
| PLA-CONTROL | .667 | .414 | 1.000 |

Pairwise Comparisons of Film_group3 DAY 4

| Sample 1-Sample 2 | Test Statistic | Sig. | Adj. Sig.[a] |
|---|---|---|---|
| PLATEC0.6-PLA | 6.000 | .014 | .043 |
| PLATEC0.6-CONTROL | 6.000 | .014 | .043 |
| PLA-CONTROL | 6.000 | .014 | .043 |

Pairwise Comparisons of Film_group3 DAY 6

| Sample 1-Sample 2 | Test Statistic | Sig. | Adj. Sig.[a] |
|---|---|---|---|
| PLATEC0.6-PLA | 6.000 | .014 | .043 |
| PLATEC0.6-CONTROL | 6.000 | .014 | .043 |
| PLA-CONTROL | 6.000 | .014 | .043 |

Each row tests the null hypothesis that the Sample 1 and Sample 2 distributions are the same.
Asymptotic significances (2-sided tests) are displayed. The significance level is .050.
a. Significance values have been adjusted by the Bonferroni correction for multiple tests.

**Table S20:** Pairwise mean values significant difference hypothesis test using Median Test results for COHESION parameter at DAY 2, DAY 4, and DAY 6.

Pairwise Comparisons of Film_group3 DAY 2

| Sample 1-Sample 2 | Test Statistic | Sig. | Adj. Sig.[a] |
|---|---|---|---|
| PLA-PLATEC0.6 | .667 | .414 | 1.000 |
| PLA-CONTROL | .667 | .414 | 1.000 |
| PLATEC0.6-CONTROL | .667 | .414 | 1.000 |

Pairwise Comparisons of Film_group3 DAY 4

| Sample 1-Sample 2 | Test Statistic | Sig. | Adj. Sig.[a] |
|---|---|---|---|
| CONTROL-PLA | .667 | .414 | 1.000 |
| CONTROL-PLATEC0.6 | 6.000 | .014 | .043 |
| PLA-PLATEC0.6 | .667 | .414 | 1.000 |

Pairwise Comparisons of Film_group3 DAY 6

| Sample 1-Sample 2 | Test Statistic | Sig. | Adj. Sig.[a] |
|---|---|---|---|
| CONTROL-PLA | 6.000 | .014 | .043 |
| CONTROL-PLATEC0.6 | 6.000 | .014 | .043 |
| PLA-PLATEC0.6 | 6.000 | .014 | .043 |

Each row tests the null hypothesis that the Sample 1 and Sample 2 distributions are the same.
Asymptotic significances (2-sided tests) are displayed. The significance level is .050.
a. Significance values have been adjusted by the Bonferroni correction for multiple tests.

**Table S21:** Pairwise mean values significant difference hypothesis test using Median Test results for COLOR parameter at DAY 2, DAY 4, and DAY 6.

Pairwise Comparisons of Film_group3 DAY 2

| Sample 1-Sample 2 | Test Statistic | Sig. | Adj. Sig.a |
|---|---|---|---|
| CONTROL-PLA | .667 | .414 | 1.000 |
| CONTROL-PLATEC0.6 | 6.000 | .014 | .043 |
| PLA-PLATEC0.6 | .667 | .414 | 1.000 |

Pairwise Comparisons of Film_group3 DAY 4

| Sample 1-Sample 2 | Test Statistic | Sig. | Adj. Sig.[a] |
|---|---|---|---|
| CONTROL-PLA | .667 | .414 | 1.000 |
| CONTROL-PLATEC0.6 | .667 | .414 | 1.000 |
| PLA-PLATEC0.6 | .667 | .414 | 1.000 |

Pairwise Comparisons of Film_group3 DAY 6

| Sample 1-Sample 2 | Test Statistic | Sig. | Adj. Sig.[a] |
|---|---|---|---|
| CONTROL-PLA | 6.000 | .014 | .043 |
| CONTROL-PLATEC0.6 | 6.000 | .014 | .043 |
| PLA-PLATEC0.6 | 6.000 | .014 | .043 |

Each row tests the null hypothesis that the Sample 1 and Sample 2 distributions are the same.
Asymptotic significances (2-sided tests) are displayed. The significance level is .050.
a. Significance values have been adjusted by the Bonferroni correction for multiple tests.

**Table S22:** Pairwise mean values significant difference hypothesis test using Median Test results for ODOR parameter at DAY 2, DAY 4, and DAY 6.

Pairwise Comparisons of Film_group3 DAY 2

| Sample 1-Sample 2 | Test Statistic | Sig. | Adj. Sig.[a] |
|---|---|---|---|
| CONTROL-PLA | .667 | .414 | 1.000 |
| CONTROL-PLATEC0.6 | 6.000 | .014 | .043 |
| PLA-PLATEC0.6 | .667 | .414 | 1.000 |

Pairwise Comparisons of Film_group3 DAY 4

| Sample 1-Sample 2 | Test Statistic | Sig. | Adj. Sig.[a] |
|---|---|---|---|
| CONTROL-PLA | .667 | .414 | 1.000 |
| CONTROL-PLATEC0.6 | 6.000 | .014 | .043 |
| PLA-PLATEC0.6 | .667 | .414 | 1.000 |

Pairwise Comparisons of Film_group3 DAY 6

| Sample 1-Sample 2 | Test Statistic | Sig. | Adj. Sig.[a] |
|---|---|---|---|
| CONTROL-PLA | .667 | .414 | 1.000 |
| CONTROL-PLATEC0.6 | 6.000 | .014 | .043 |
| PLA-PLATEC0.6 | 6.000 | .014 | .043 |

Each row tests the null hypothesis that the Sample 1 and Sample 2 distributions are the same.
Asymptotic significances (2-sided tests) are displayed. The significance level is .050.
a. Significance values have been adjusted by the Bonferroni correction for multiple tests.
